# Discovery of First-in-Class Inhibitors Targeting a Pathogen-Associated Aminoglycoside-Resistance 16S rRNA Methyltransferase

**DOI:** 10.1021/acsinfecdis.5c00297

**Published:** 2025-07-11

**Authors:** Debayan Dey, Benjamin E. Deprez, Natalia Zelinskaya, Jose M. Castro, William M. Wuest, Graeme L. Conn

**Affiliations:** † Department of Biochemistry, 12239 Emory University School of Medicine, Atlanta, Georgia 30322, United States; ‡ Department of Chemistry, 1371 Emory University, Atlanta, Georgia 30322, United States; § Graduate Program in Biochemistry, Cell and Developmental Biology (BCDB), 1371 Emory University, Atlanta, Georgia 30322, United States

**Keywords:** antibiotic resistance, rRNA, methyltransferase, high-throughput virtual screening, precision docking, chemoinformatics

## Abstract

Among several distinct mechanisms used by bacteria to circumvent antibiotic stress, a predominant form of resistance to ribosome-targeting compounds is the methylation of their ribosomal RNA (rRNA) binding sites. The acquisition of aminoglycoside-resistance methyltransferases that modify 16S rRNA nucleotides in the ribosome decoding center, for example, results in exceptionally high-level aminoglycoside resistance and poses a major threat to their future clinical utility. Here, we report the discovery of a first-in-class panel of small-molecule inhibitors that target a previously unexploited composite “Y-shaped” binding pocket that is unique to the 30S subunit (substrate)-bound form of the 16S rRNA (m^1^A1408) methyltransferase NpmA. This Y-shaped pocket, formed by the conserved *S*-adenosyl-l-methionine binding site and a channel in which A1408 is positioned for modification, was predicted by molecular dynamics simulations to be accessible and potentially druggable in the free enzyme. We therefore conducted high-throughput virtual screening of over 2 million compounds, followed by precision docking and chemoinformatics to select lead scaffolds for initial testing. Iterative experimental analysis and docking of analogs to top hits led to the discovery of three compounds with comparable NpmA inhibitory activity and other similar analogs unable to inhibit the enzyme. Structure–activity relationship analysis highlighted the importance of stereoselectivity, halogen−π interactions, and water-mediated binding. Our strategy provides a new model for methyltransferase inhibitor development, targeting conformationally adaptive and composite binding sites and could be applied to efforts to develop inhibitors of other clinically prevalent resistance determinants such as the aminoglycoside-resistance m^7^G1045 methyltransferases (e.g., RmtB).

## Graphical abstract

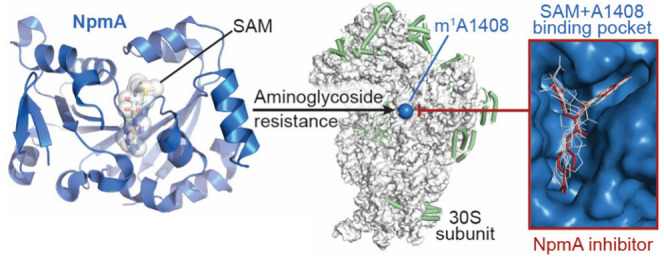

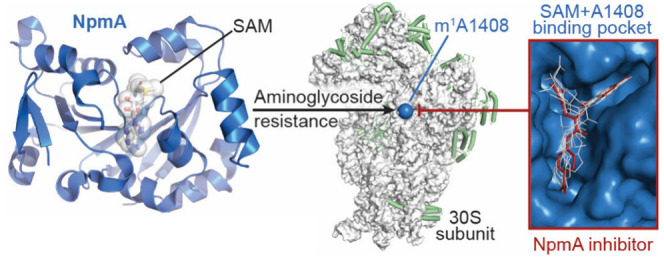

Bacterial resistance to antibiotic treatment is one of the greatest threats to modern healthcare and is projected to cause millions of deaths annually and billions in associated healthcare costs and lost productivity.^1–3^ The impacts of increasing clinical antibiotic resistance have been exacerbated by a sharp decline in approved treatments and new drug development for bacterial infection over the last several decades, leading to speculation that we may enter a “post-antibiotic era” with the loss of many advances of modern medicine.^4^ There is therefore an urgent need to develop a deeper understanding of resistance mechanisms to enable new strategies to counter this problem, including the development of new antibiotics or synergistic adjuvants to restore the activity of approved drugs lost to resistance.

Aminoglycosides are potent ribosome-targeting bactericides that have long been a critical component of our antibacterial drug arsenal.^5^ Despite their replacement over the last several decades by newer generations of other antibiotic classes, several aminoglycosides, including gentamicin, amikacin, netilmicin, tobramycin, and the most recently approved drug plazomicin, remain in use today to treat severe hospital-based infections with Gram-negative pathogens.^6–9^ Aminoglycosides are also effective against Gram-positive bacteria, including mycobacteria.^10^ Increasing resistance to many clinically used antibiotics has led to a resurgence of interest in aminoglycosides, but their wider application is also threatened by increasing resistance.

Historically, clinical failure of aminoglycosides has been ascribed to the action of aminoglycoside-modifying enzymes (AMEs), and significant effort has been expended to develop semisynthetic aminoglycosides that evade their action.^11^ Plazomicin, for example, is recalcitrant to the majority of AMEs with strong activity against multiple bacterial pathogens.^7,8,12^ In sharp contrast, like all other aminoglycosides currently in use in the clinic, plazomicin is rendered completely ineffective by the action of the aminoglycoside-resistance 16S rRNA (rRNA) methyltransferase enzymes.^12^ These enzymes were originally identified as a major mechanism of self-protection in aminoglycoside-producing bacteria but have been acquired by pathogenic bacteria and identified over the past decade as a major new threat to aminoglycoside use.^13–16^

The 16S rRNA aminoglycoside-resistance methyltransferases form two families of *S*-adenosyl-l-methionine (SAM)-dependent enzymes that modify either N1 of A1408 or N7 of G1405 (to form m^1^A1408 and m^7^G1405, respectively) within the aminoglycoside binding site of the bacterial small (30S) ribosomal subunit.^17–20^ The m^7^G1405 modification confers exceptionally high-level resistance to all 4,6-disubstituted 2-deoxystreptamine (4,6-DOS) aminoglycosides, including most clinically useful drugs.^21^ In contrast, the ring configuration of 4,5-DOS aminoglycosides, e.g., apramycin and neomycin, leaves these drugs unaffected by the m^7^G1405 modification, and semisynthetic analogs of these drugs have retained activity.^22^ However, the m^1^A1408 modification remains a threat as it also confers high-level resistance to a distinct set of aminoglycosides, including some members of both the 4,6-DOS and 4,5-DOS aminoglycosides.^14^ Although currently less extensively identified in clinical isolates, the m^1^A1408 methyltransferases (NpmA-C) are globally disseminated and have been identified in , *Klebsiella pneumoniae*, and .^14,23–25^ Further, identification of *Clostridioides difficile* harboring NpmA has led to speculation that hospital-acquired *C. difficile* may serve as a reservoir for future spread of this resistance mechanism.^26^

We selected the m^1^A1408 methyltransferase NpmA for identification and initial development of an “antibiotic resistance breaker” due to its broad aminoglycoside resistance profile and the availability of high-resolution structures of both free and 30S subunit-bound enzyme.^17,27^ Antibiotic resistance breakers are compounds that directly target resistance determinants thereby restoring the efficacy of currently approved treatments.^28,29^ For example, β-lactamase inhibitors have been highly successful in extending β-lactam efficacy and are clinically used worldwide (e.g., the combination of clavulanic acid and amoxicillin),^30^ and this strategy has also been recently applied to inhibition of efflux, e.g., NorA in .^31^ However, to our knowledge, to date, development of resistance breakers has not been applied to aminoglycoside resistance mediated by rRNA modification. Leveraging available structural insights into NpmA, we identified a “Y-shaped” pocket comprising the SAM binding site and a channel that positions A1408 for modification. Notably, the A1408 channel is lined by two Trp residues (W107/W197) that are universally conserved among the m^1^A1408 methyltransferases.^17,27^ Structure-based high-throughput virtual screening (HTVS), ligand docking, *in vitro* methylation assays, and molecular dynamics (MD) simulations were subsequently used to identify, validate, and characterize lead compounds as first-in-class NpmA inhibitors. These studies thus lay a foundation for development of novel antibiotic resistance breakers that can be paired with existing or next-generation aminoglycosides to combat resistant bacterial infections.

## Results and Discussion

### Identification of a Unique Druggable Pocket in NpmA

Targeting only the cosubstrate SAM binding site of NpmA could result in inhibitors with off-target effects, as this site is conserved across many bacterial and human methyltransferases. When bound to its 30S subunit substrate, however, a larger continuous potential ligand binding site is formed by the SAM binding site and a channel in which A1408 is positioned for modification (Figure 1A). The conserved SAM binding site can also be subdivided into two distinct pockets that contain the adenosine nucleoside and methionine moieties. These two regions of SAM form extensive interactions with NpmA: residues P56, E88, L110, and T109 with the nucleobase, G34, D55, and T33 with ribose sugar, and N38, S195, and L196 with the methionine (Figure 1B). NpmA binding to the 30S subunit promotes stabilization of a local conformational reorganization in which A1408 is “flipped” out of helix 44 (h44) and intercalated between W107 and W197, with additional stabilizing contact from F105.

**Figure 1:**
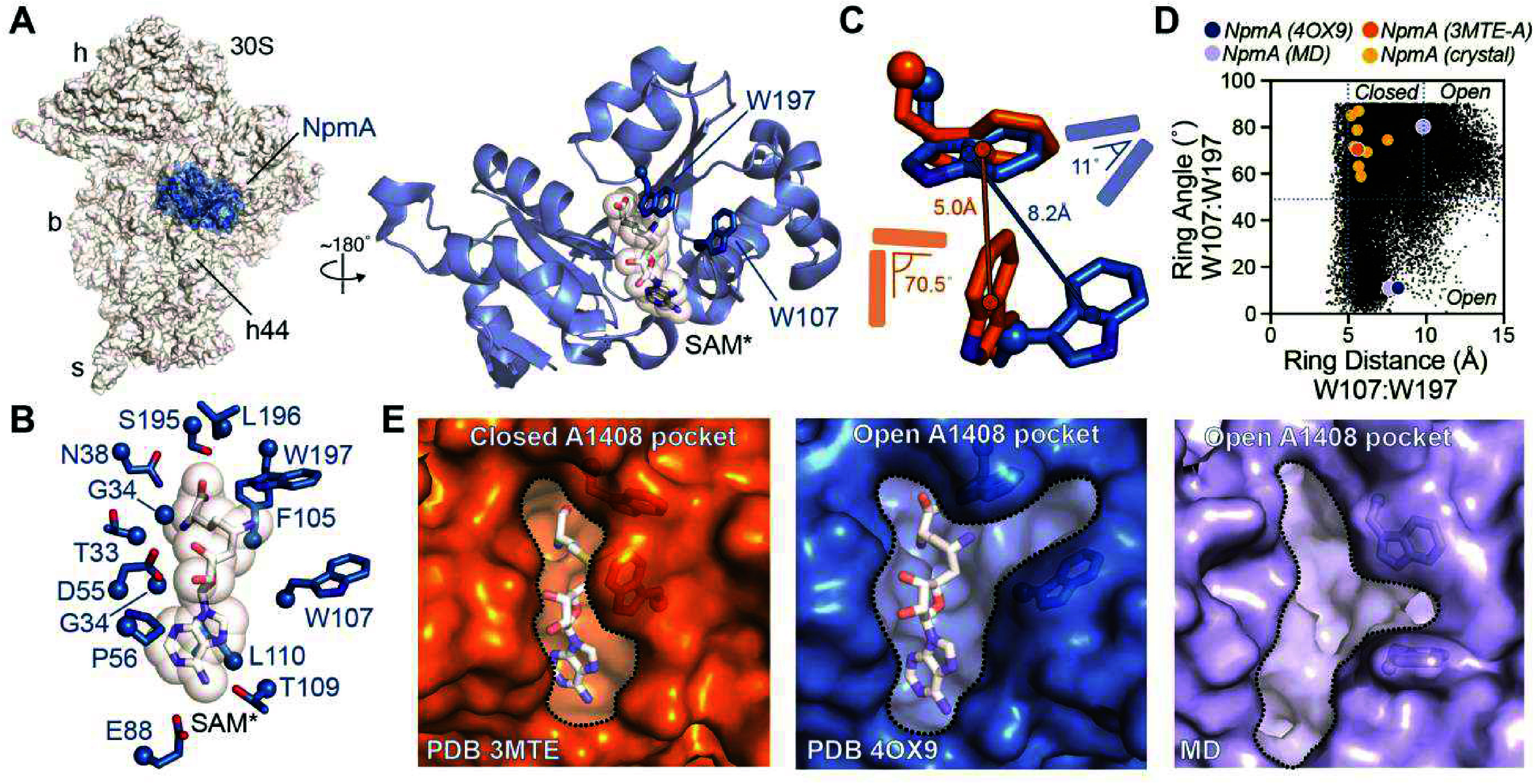
Binding pocket identification in the 30S-bound structure of NpmA. (A) Structure of NpmA bound to the bacterial 30S subunit (PDB 4OX9). The zoomed-in view (right) is rotated by ∼180° to show the subunit binding interface view and highlights the features forming the target binding pocket: the SAM binding site and channel between Trp107 and Trp197 that is occupied by the flipped A1408 nucleobase. 30S subunit features head (h), body (b), and spur (s) are indicated; the asterisk (SAM*) denotes that the SAM binding pocket is occupied by sinefungin in the 30S-NpmA complex crystal structure. (B) NpmA residues forming the SAM binding site. (C) Comparison of Trp107 and Trp197 orientations in 30S-bound NpmA (blue) and an example free NpmA (PDB entry 3MTE, chain A). The distance and angle metrics used to characterize the relative Trp residue orientations are indicated. (D) Plot of Trp107-Trp197 distance and angle for 30S-bound NpmA (blue), free NpmA (PDB 3MTE chain is colored dark orange; all other chains in 3MTE, 3P2I, 3P2A, 3PB3, and 3P2K are colored light orange), and representative structures from MD (clusters 4 and 5; light blue). (E) View of the binding pockets in free (PDB 3MTE, chain A), 30S-bound (PDB 4OX9), and a representative structure from MD simulation (cluster 4).

The presence of the A1408 binding channel between W107 and W197 is dependent on the position and relative orientations of these two residues (Figure 1C). Among available structures, only in the 30S-bound state of NpmA (PDB entry 4OX9) are Trp107/Trp197 positioned to allow formation of the contiguous Y-shaped pocket comprising the SAM-binding site and the A1408-binding channel. In this conformation, the indole rings of W107 and W197 are separated by 8.2 Å with an interplanar angle of 11° (Figure 1C), positioning them nearly coplanar to facilitate π–π interactions with the A1408 base. We refer to this as the “open” state of the Y-shaped pocket. In contrast, in all currently available structures of free NpmA, W107 exhibits significant flexibility (Figure S1A) that results in a number of distinct “closed” states of the A1408-binding channel, primarily characterized by a shorter inter-Trp distance (Figure 1C,D).

We reasoned that the unique Y-shaped binding pocket (i.e., in the NpmA open state) could present a promising strategy to identify specific NpmA inhibitors, as it is distinct from the canonical SAM binding site. To first assess whether this Y-shaped pocket might be present in solution in the absence of 30S binding and SAM, we performed 3 × 500 ns MD simulations of *apo* NpmA and focused on the dynamics of W107 and W197. Consistent with observations from the crystal structures, residue root-mean-square fluctuation (RMSF) reveals W107 to be more dynamic than W197 (Figure S1B). Cluster analysis and selection of representative structures from each of the top five most populated conformational states revealed the presence of both closed and open states of the pocket during the simulation. In three clusters, the Trp107-Trp197 interplanar distances and angles result in a closed binding pocket, whereas the other two clusters possess distinct open states, i.e., with a Y-shaped pocket including the open A1408 channel (Figure 1D,E and Figure S1C–G). The two distinct open states are characterized by an intermediate interplanar distance and narrow angle or longer distance (>∼10Å) and wider angle; in contrast, all structures with interplanar distances <6.5 Å and angles >50° are defined as being in a closed state. Most importantly, the MD analysis reveals that the Y-shaped pocket is accessible in free NpmA (Figure 1E) and exists in approximately 40% of NpmA conformational states in the absence of 30S subunit binding. Thus, targeting this dynamic pocket in the *apo* state is a feasible strategy for selective inhibitor development that leverages a unique structural feature of NpmA that is normally promoted during its interaction with the substrate.

### Structure-Based HTVS Identifies Potential Leads for NpmA Inhibition

We targeted the newly validated Y-shaped pocket as the basis for HTVS to identify potential inhibitors of NpmA (Figure 2A). A ChemBridge CORE library of over 2 million compounds was generated with LigPrep and then screened using the Glide HTVS protocol within the Schrödinger Suite (version 2024-1). The top 10% of compounds (∼130,000) based on HTVS scores progressed to Glide Standard Precision (SP) docking,^32^ and the top 10% of SP-ranked compounds (∼13,000) were then subjected to Glide Extra Precision (XP) docking to enhance accuracy (Figure 2B and Table S1). Compounds with docking scores below −6 kcal/mol (corresponding to the top ∼30% XP-ranked compounds) were selected for analysis to guide the selection of an initial set of commercially available test compounds with diverse physicochemical properties.

**Figure 2:**
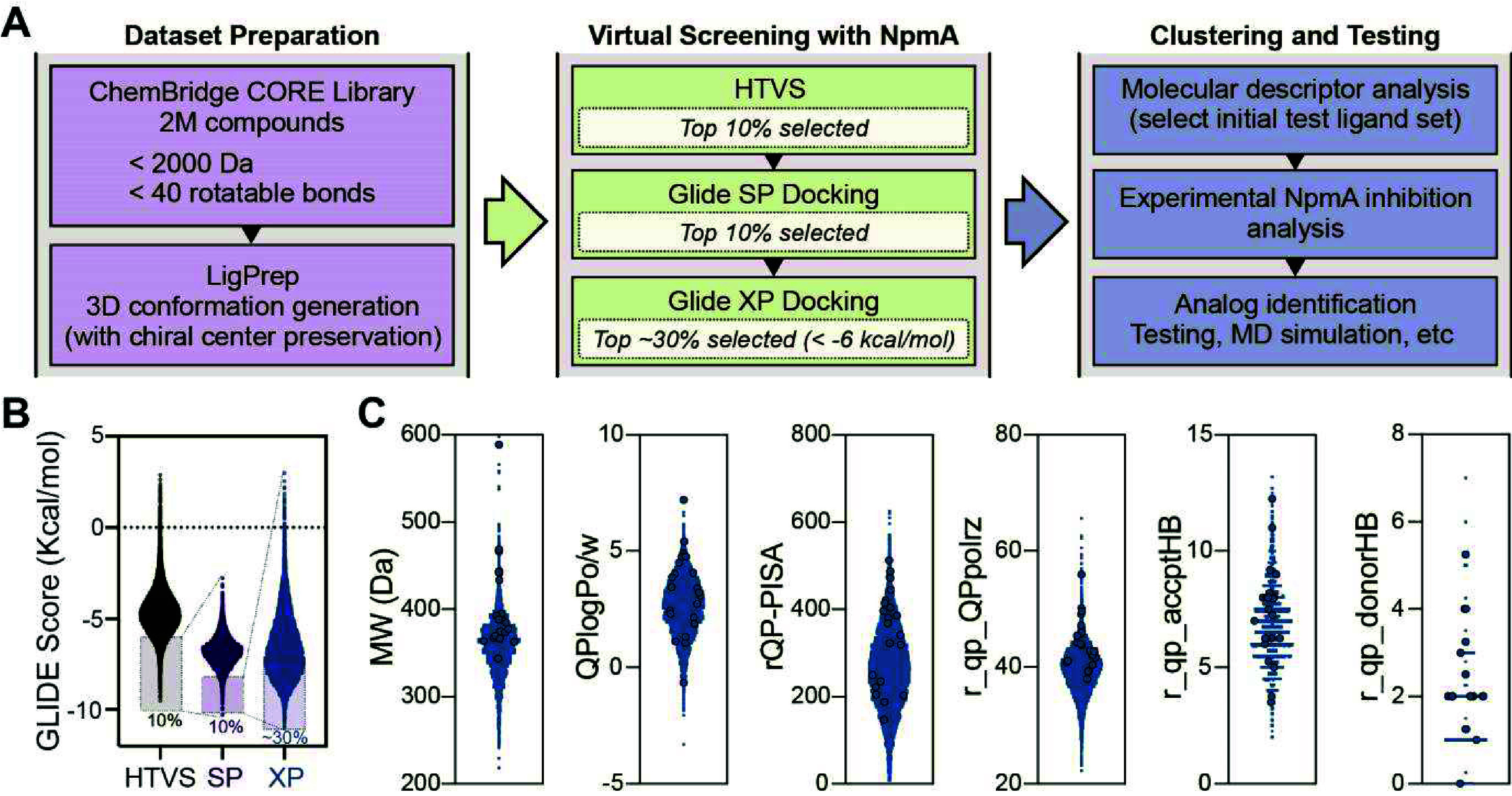
Identification of potential NpmA inhibitors by virtual screening. (A) Overview of the computational and experimental workflow used for NpmA inhibitor identification and characterization. (B) Results of HTVS and subsequent redocking by Glide SP and Glide XP with progression criteria indicated. (C) Cheminformatic analyses of ligands with Glide XP scores <−6 kcal/mol (∼30%) and selection of an initial structurally and chemically diverse set of 22 commercially available ligands for testing (circles). Plots shown are compound molecular weight (MW (Da)), lipophilicity estimate (QPlogPo/w), van der Waals surface area for π-interactions (rQP-PISA), polarizability (QPpolrz), and the number of potential hydrogen bond donors (r_qp_donorHB) and acceptor (r_qp_acceptHB).

Following fingerprint-based similarity analysis and K-means clustering in Schrödinger’s Discovery Informatics Suite, we considered a combination of molecular features and drug-likeness parameters to capture a broad range of chemical diversity (Figure 2C): compound molecular weight (MW), lipophilicity estimate (QPlogPo/w), available van der Waals surface area for π-interactions commonly associated with aromatic rings and important for stacking interactions (rQP-PISA), polarizability, which influences properties such as intermolecular interactions and reactivity (QPpolrz), and the number of potential hydrogen bond donors (r_qp_donorHB) and acceptors (r_qp_acceptHB). Based on this analysis, we selected a set of 22 structurally and chemically diverse compounds, including those with both approximately Y-shaped (**1**–**9**) or linear structures (**10**–**22**) to ensure a comprehensive coverage of distinct chemical scaffolds and binding modes for downstream validation (Figure S2).

### Iterative *in Vitro* Testing and *in Silico* Analysis Discovers First-in-Class Inhibitors of NpmA

To evaluate the inhibitory potential of compounds **1**–**22**, we performed assays of inhibition of 30S subunit *in vitro* methylation by NpmA using purified recombinant NpmA, 30S subunit, [^3^H]-SAM, and each compound at 1 mM. Reduced ^3^H incorporation relative to a no-compound control reflected NpmA inhibition, and three compounds, **3**, **10**, and **20**, were identified that reduced NpmA activity to <25% (Figure 3A). Compound **3** binds to the target pocket in a Y-shaped conformation (as predicted for **1**–**9**), simultaneously engaging both the A1408 channel and both parts of the SAM-binding site, while **10** and **20** adopt a more linear conformation (as predicted for **10**–**22**) that spans the SAM adenine nucleoside pocket and A1408 channel but not the SAM methionine pocket (Figure 3B).

**Figure 3:**
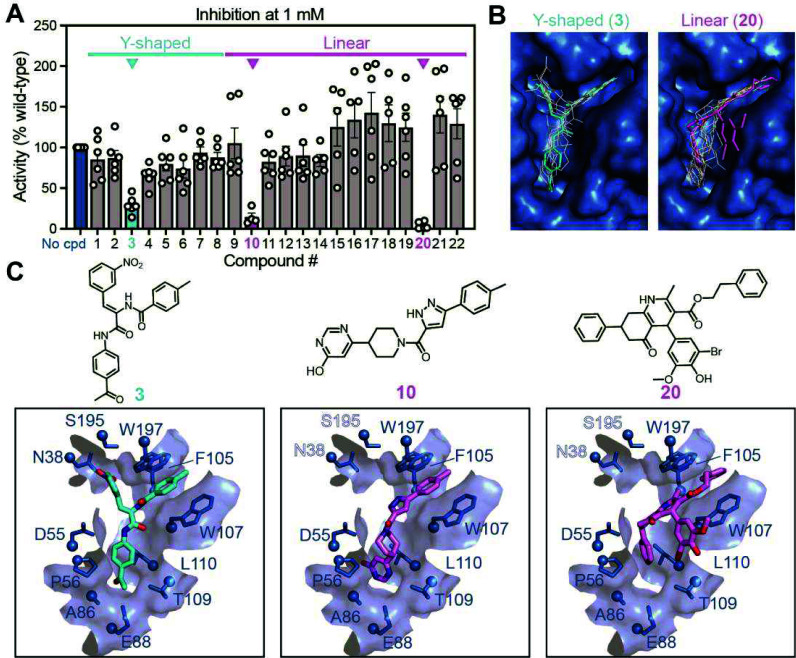
Identification of initial lead inhibitors of NpmA. (A) Inhibition of NpmA methyltransferase activity at a fixed concentration of compounds **1**–**22**, relative to the no-compound control (blue). Compounds resulting in <25% activity are highlighted for the Y-shaped (**3**; cyan) and linear (**10** and **20**; magenta) scaffolds. (B) Docking of all compounds in the SAM/A1408 pocket in NpmA. (C) Chemical structures (top) and zoomed in views of the ligand binding pocket with docked compounds **3**, **10**, and **20**.

Compound **3** is composed of three aryl moieties, connected by a dehydroamino amide core (Figure 3C, left), which results in potential (*Z*)- and (*E*)-configurations (Figure S3). Docking studies revealed that the (*Z*)-stereoisomer binds most favorably with a Glide XP score of −9.9 kcal/mol (Table S1), whereas no favorable binding poses could be identified for the (*E*)-stereoisomer, highlighting the potentially critical role of stereoselectivity in NpmA binding. In the (*Z*)-configuration, the meta-nitrophenyl group is positioned within the SAM methionine-binding pocket of NpmA, forming interactions with residues N38 and S195 (Figure 3C, left). The para-acetophenone fits into the SAM adenosine binding pocket, engaging residues A86 and E88. The third aryl group, bearing a para-methyl substituent, is intercalated between W107 and W197 within the A1408 channel and engages in additional hydrophobic interactions with F105 (Figure 3C, left). Thus, as anticipated, **3** engages with many of the same key residues within the composite ligand pocket as SAM and the target A1408 base.

Compound **10** adopts a linear configuration of its four ring systems (Figure 3C, center). In the Glide XP docked pose (−8.9 kcal/mol; Table S1), 4-hydroxypyrimidine is well-positioned by stabilizing interactions within the SAM adenosine pocket of NpmA. The para-methyl phenyl group, similar to that in **3**, inserts into the A1408 channel, where it is intercalated between W107 and W197, and engages in additional hydrophobic interaction with F105. This conserved favorable interaction between the aromatic group and the A1408 channel is observed in both Y-shaped compound **3** and linear compound **10**, while the latter does not extend into the SAM methionine pocket and therefore does not contact residues N38 and S195 (Figure 3C). Compound **20** also adopts a linear configuration of its four ring systems: two terminal unsubstituted phenyl rings and a highly substituted phenyl ring connected to a central polyhydroquinoline core (Figure 3C, right). Glide XP docking of **20** yielded a score of −7.2 kcal/mol (Table S1) with the docked pose showing one terminal phenyl and the polyhydroquinoline moiety intercalated between W107 and W197 in the A1408 channel. The second phenyl ring is oriented toward the SAM adenosine region, resulting in a partially filled Y-shaped pocket with the SAM methionine region unoccupied and no contacts with residues N38 and S195 (Figure 3C, right).

We selected **3** as the basis for further exploration of chemical space due to its favorable inhibitory activity and binding characteristics, including its distinct chemical scaffold that is aligned well with key features of the full Y-shaped pocket of NpmA. To identify structurally related analogs, we conducted a focused search within the ChemBridge database using cheminformatics tools to mine derivatives. Glide XP docking was repeated for all identified analogs to assess their alignment with the Y-shaped pocket and maintenance of key interactions, and a resulting set of 18 additional analogs (**23**–**40**; Figure S4) were prioritized and purchased for experimental validation. As before, all compounds were tested for their ability to inhibit NpmA methyltransferase activity but this time at a concentration of 500 μM to better discriminate among these structurally more similar compounds. Of this additional set, compounds **23**, **27**, **33**, **39**, and **40** reduced NpmA activity to <25% compared to the no-compound control (Figure 4A). We further assessed **23**, which appeared to show the greatest inhibition in this initial fixed concentration screen, and determined an IC_50_ of 76 μM for NpmA inhibition (Figure 4B).

**Figure 4:**
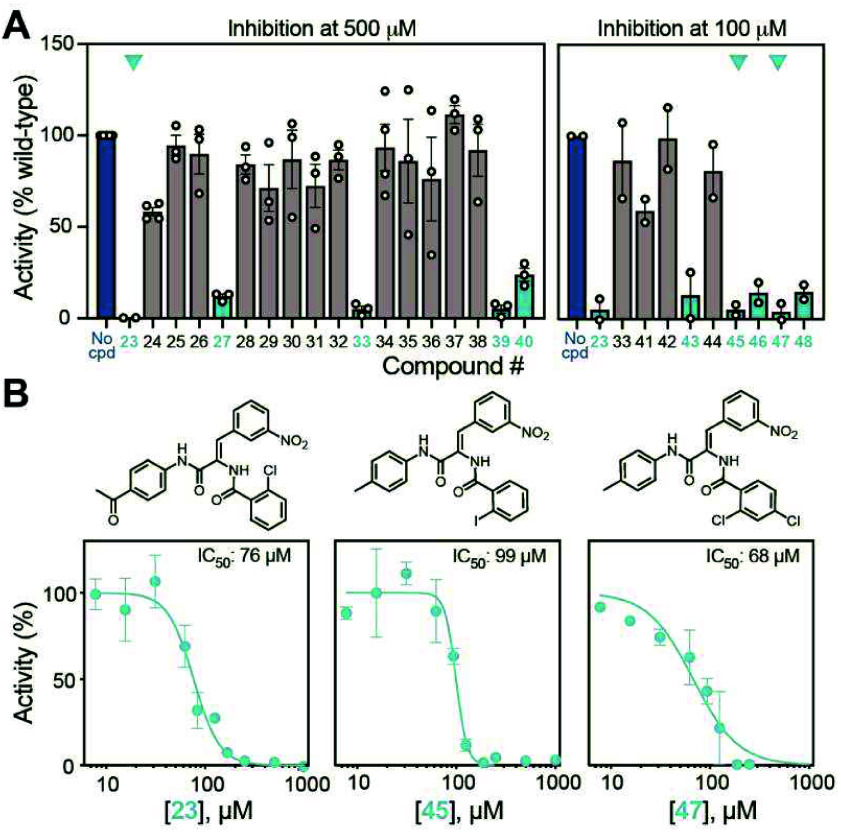
Identification of compounds with increased NpmA inhibition based on similarity to compound **3**. (A) Fixed concentration inhibition with the first set of compounds selected based on similarity to compound **3** (compounds **23**–**40**, at 500 μM) and a subsequent set (compounds **41**–**48**, at 100 μM). Highlighted compounds (cyan) result in <25% activity NpmA relative to the no-compound control (blue). (B) Chemical structures (top) and measurements of IC_50_ values (bottom) for the three most active Y-shaped ligands from panel (A): **23**, **45**, and **47**.

To confirm the results of the initial inhibition screen, we synthesized and retested the top hit, compound **23** (Figure S5A). Commercially available phosphonate **49** was hydrolyzed to the free carboxylic acid, which underwent amide coupling with 4′-aminoacetophenone in moderate yield. Carbamate deprotection of **51** and subsequent amide coupling of unstable free amine intermediate **52** gave **53**. The Horner–Wadsworth–Emmons reaction mediated by Et_3_N/LiCl afforded **23** without detectable formation of the (*E*)-diastereomer. The (*Z*)-olefin geometry of **23** was determined by NMR using 1D-selective NOESY experiments, and the ^1^H and ^13^C spectra of the synthesized compound were identical with those of the purchased sample. Purchased samples of **3** were also evaluated using the same NMR workflow to confirm the expected (*Z*)-olefin geometry (Figure S5B). Both resynthesized and commercial samples of compound **23** were shown to possess the (*Z*)-olefin geometry by NMR, and they inhibited NpmA to the same extent (Figure S5C). This result validates that the active (*Z*)-isomer is consistent with the modeled binding pose.

In a final iteration, we focused on a set of refined structural analogs of compound **23**, preserving the core scaffold and maintaining the Y-shaped binding profile, to begin exploring structure–activity relationships (SARs) across different regions of the scaffold. We selected compounds **41**–**48** (Figure S4) for testing, along with a re-evaluation of **23** and **33**, at a fixed concentration of 100 μM to further the stringency of the first activity screen. As expected, **23** continued to show strong inhibitory activity, whereas compound **33** showed poor inhibition, suggesting that solubility issues had impacted the apparent activity at higher concentration. Among the new analogs, **43**, **45**, **46**, **47**, and **48** inhibited NpmA activity with <25% activity observed compared to the no-compound control (Figure 4A). Based on their apparent inhibition activity, comparable to **23**, IC_50_ values of 99 μM and 68 μM were determined for **45** and **47**, respectively, making **47** the most potent analog in this series identified (Figure 4B).

### Structure–Activity Relationship of NpmA Inhibitors

Using the docking models, we next attempted to understand the possible binding poses, their energetics, key residue contributions, and the basis of stereoselection. Our docking studies revealed that for Y-shaped compounds the (*Z*) isomer is generally favored for binding to NpmA, while the (*E*)-isomer is disfavored in most cases (Table S1). The extent of this stereochemical preference varies across the Y-shaped compound series (**3**, **23**–**48**), for which we docked both (*Z*)- and (*E*)-isomers into the NpmA binding pocket. Notably, for **3**, **23**, **45**, and **47**, the (*Z*)-isomer consistently demonstrated more favorable binding conformations, supporting its role in driving optimal pocket engagement.

To understand the stereochemical basis of binding, we compared docking poses of **23** in both (*Z*)- and (*E*)-configurations (Figure S6A,B). In both cases, the nitroarene group inserts into the SAM methionine pocket, forming interactions with N38 and S195. In the (*Z*)-isomer (Glide XP, −9.9 kcal/mol), the ortho-chlorophenyl ring intercalates between W107 and W197, while the phenyl ring with a para-acetone group forms stabilizing contacts with T109 and E88. In contrast, the (*E*)-isomer (Glide XP, −6.6 kcal/mol) adopts a flipped orientation: the acetophenone intercalates between the Trp residues, while the chlorophenyl ring instead engages the SAM adenosine-binding pocket, forming a less favorable interaction. These observations highlight the critical role of the olefin geometry of this scaffold in determining the binding conformation and affinity of these Y-shaped inhibitors within the NpmA pocket.

Docking studies of **45** and **47** indicate a preferred binding conformation in the (*Z*)-isomer (Table S1), where the halogenated phenyl ring intercalates between W107 and W197, forming stabilizing aromatic interactions (Figure S6B,C). Meanwhile, the methyl group on the toluene moiety engages in hydrophobic interactions with the main chain of A87 and the side chain of T109, contributing further to binding affinity within the Y-shaped pocket of NpmA. When a phenyl ring is positioned between two tryptophan (Trp) residues, it can participate in π–π stacking or edge-to-face interactions with the indole rings; however, these interactions are typically moderate due to the limited polarizability and electron distribution of an unsubstituted phenyl ring. In contrast, halogenated phenyl rings such as those substituted with chlorine or iodine, as in compounds **23**, **47**, and **45**, tend to form stronger and more directional interactions. Halogenation enhances the overall polarizability of the aromatic ring, increasing van der Waals and dispersive interactions with the surrounding indole environment. In contrast, (*Z*)-olefins **42** and **44** bind poorly, as reflected by both unfavorable Glide XP docking scores and weak inhibition in the *in vitro* assay. This reduced activity may be attributed to the presence of a benzoic acid (Figure S6E,F), which occupies the SAM adenosine pocket and likely clashes with key residues, such as E88 or T109, thereby disrupting stable binding.

We performed Molecular Mechanics Generalized Born Surface Area (MMGBSA) analysis after XP docking to refine binding affinity estimates by incorporating solvation and entropic effects not fully captured in docking scores. Energy component breakdown from MMGBSA also provides insight into several key trends in the factors driving NpmA-compound interaction for the four (*Z*)-isomer compounds with good NpmA inhibition activity (**3**, **23**, **45**, and **47**) and the two close analogs with weaker activity (**46** and **48**; Table S2). Notably, **23** exhibits the most favorable Coulombic contribution (−27.8 kcal/mol), which describes its electrostatic binding strength. Hydrogen bonding contributions remain modest across all compounds (−0.6 to −3.1 kcal/mol), with **23** again showing the strongest value (−3.1 kcal/mol). Solvation penalties (Solv GB), which oppose binding, range from +28.6 to +33.5 kcal/mol and are relatively consistent across the set. Interestingly, compound **47** stands out with the most favorable van der Waals (−64.5 kcal/mol) and lipophilic (−36.0 kcal/mol) contributions, although these are partially offset by its weaker Coulombic (−16.0 kcal/mol) and solvation (+29.2 kcal/mol) terms. Despite this, **47** achieves a strong overall binding free energy (−96.5 kcal/mol), suggesting a robust interaction profile. For **46**, the reduced binding, consistent with weaker NpmA inhibition, may be attributable to the higher predicted solvation penalty (+33.5 kcal/mol) and less favorable van der Waals interactions (−57.7 kcal/mol) despite a relatively strong Coulombic term (−26.2 kcal/mol). Overall, **48** shows the least favorable hydrogen bonding (−0.6 kcal/mol) and weakest van der Waals contribution (−54.6 kcal/mol) among the group, indicating suboptimal packing. The combination of higher solvation costs and weaker nonpolar interactions likely underlies the reduced inhibitory ability of both **46** and **48**, and overall, the MMGBSA analyses reveal that van der Waals and lipophilic interactions are the dominant driving forces of binding across these compounds, while Coulombic and solvation terms modulate the net affinity.

Overall, the structure–activity relationship (SAR) emerging from this compound series suggests that modifications to the ring interacting with the SAM adenosine pocket could enhance binding by introducing amine or positively charged groups or by converting the ring to a heterocycle, thereby improving interactions within the pocket, particularly with residue E88. For the ring engaging with the A1408 channel, the presence of a halogenated phenyl group appears favorable; however, further exploration of halogenated phenols or heterocyclic variants could potentially strengthen interactions with Trp residues W107 and W197. Additionally, for the ring interacting with the SAM methionine pocket, the nitro group may be replaced with alternative electron-withdrawing or polar substituents to fine-tune the binding affinity and optimize contacts within the methionine-binding region.

### Dynamics of NpmA with Inhibitors to Probe Water-Mediated Interactions

To explore the role of water molecules in ligand binding dynamics, ligand binding stability, and the conformational dynamics of Trp107 and Trp197 in the presence of a bound ligand, we performed three independent 100 ns replicate MD simulations for **23**, **45**, and **47**. Comparison of RMSF profiles between *apo* NpmA and the NpmA–**23** and NpmA–**47** complexes revealed reduced flexibility upon ligand binding, particularly near E88, W107, and W197, indicating localized stabilization (Figure 5A). The overall decrease in RMSF also suggests that **23** and **47** contribute to a generally more stable complex. In contrast, the RMSF profile of NpmA–**45** shows a reduction near W107, but RMSF remains comparable to *apo* NpmA in other ligand-binding regions. This may indicate higher dynamicity for **45** compared to **23** and **47**, potentially due to the substitution of iodine on the phenyl ring, which intercalates between W107 and W197. A potential for steric clash might slightly adjust the binding pose compared to that predicted in XP Glide docking.

**Figure 5:**
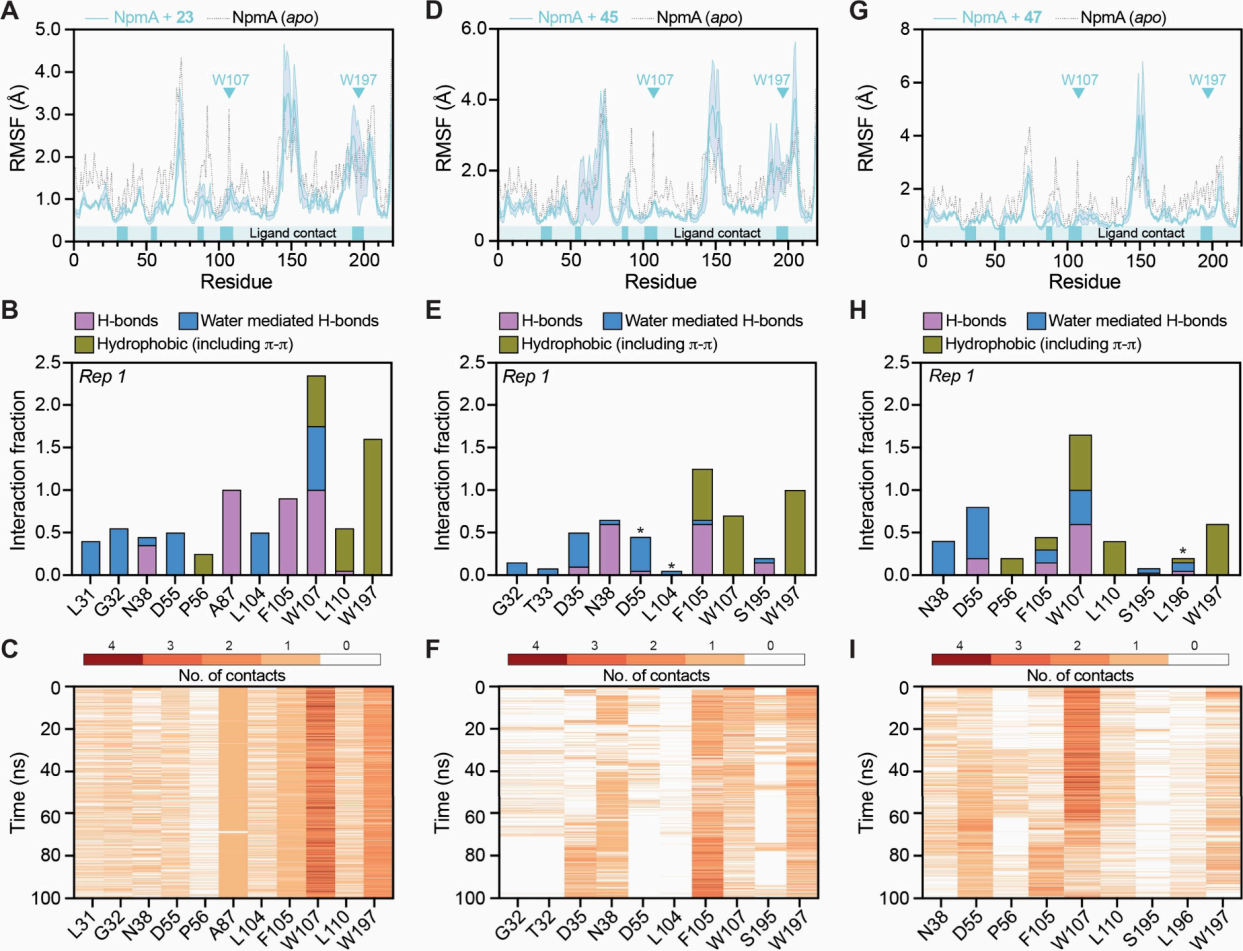
NpmA-inhibitor interactions during MD simulation. (A) Comparison of residue root-mean-square fluctuation (RSMF) for *apo* (black) and **23**-bound NpmA (cyan) with locations of Trp107/Trp197 highlighted and all NpmA-**23** contact sites indicated in the bar below the plot. (B) Summary of interaction types made with 11 key contact residues in the SAM/A1408 binding pocket. (C) Stability of contacts at the same 11 residues during the 100 ns MD simulation (data shown are for replicate 1 of 3). (D–F; G–I) As for panels A–C but for compounds **45** and **47**, respectively.

Residue-level interaction analyses of compounds **23** and **47** revealed similarities in the residue contributions to their respective interactions. Water-mediated hydrogen bonds form with L31, G32, D55, and L104. Hydrophobic and π-mediated interactions are mainly contributed by P56, L110, and W197, with W197 showing the strongest π-stacking interaction with the chlorophenyl ring of **23** and the dichlorophenyl group of **47** (Figure 5B). Among all NpmA residues identified, W107 emerged as the most critical, consistently forming more than two contacts per frame on average across replicates and involving a combination of direct hydrophobic, π-mediated, and water-bridged interactions (Figure 5C). Both **23** and **47** also form hydrogen bonds with N38, A87, F105, and W107. Only minor replicate-to-replicate variations were observed in the NpmA–**23** simulations (Figure S7A,B), suggesting a robust and reproducible binding pose. In simulations of NpmA–**45**, consistent with the higher RMSF values observed compared to NpmA–**23** and NpmA–**47**, we found that the binding pose is slightly shifted, showing additional interactions with D35 and S195, while contributions from P56, G31, and L110 are reduced. While some variation was also noted across replicates, compound **45** continues to occupy the Y-shaped pocket. The presence of the larger halide group likely drives these local readjustments. Overall, MD simulations confirm consistent binding of all compounds to the Y-shaped pocket, with key residue contributions and significant water-mediated interactions supporting stable complex formation.

## Conclusion

Through the use of a virtual high-throughput screen, we were able to target a unique Y-shaped pocket derived from the substrate-bound state of the NpmA substrate. This enabled the discovery and detailed characterization of first-in-class small molecule inhibitors of this pathogen-associated aminoglycoside-resistant 16S rRNA methyltransferase. Docking and MD simulations highlighted the critical role of stereoselectivity, π–π interactions, and water-mediated hydrogen bonding in stabilizing ligand binding. Notably, halogenated phenyl groups, especially those bearing chlorine or iodine, significantly enhanced the interaction with W107 and W197 via halogen−π interactions. Our strategy, and the first NpmA inhibitors it provided, opens a new front in efforts to counter resistance mediated by the aminoglycoside-resistance 16S rRNA methyltransferases, which have emerged as a major threat to aminoglycoside efficacy. By targeting a dynamic, substrate-induced Y-shaped pocket unique to NpmA, this work offers a new framework for the rational design of selective inhibitors that exploit conformational plasticity rather than conserved cofactor sites. Given the structural and mechanistic similarities among 16S rRNA methyltransferases, including clinically prevalent enzymes that modify G1405 (e.g., RmtB), these inhibitors could serve as valuable starting points for developing broadly effective antibiotic resistance breakers. Furthermore, their combination with next-generation aminoglycosides such as plazomicin, which is already optimized against the clinically prevalent AMEs, could result in potent synergistic therapies capable of bypassing multiple layers of bacterial resistance, including those coharbored with genes like NDM1. As such, this work can serve as an important starting point for the development of new molecules that can ultimately restore the clinical utility of aminoglycosides against multidrug-resistant pathogens.

## Materials and Methods

### HTVS and Precision Docking

Ligands for HTVS were sourced from the ChemBridge CORE library which was selected for its extensive size (∼2 million compounds) and commercial availability. Retrieved structures were processed using the LigPrep module in the Schrödinger software (version 2024-1) to generate 3D conformers and optimize protonation states with the OPLS4 force field.^33^ A Y-shaped target ligand binding pocket in the open state of NpmA (extracted from PDB entry 4OX9) was prepared as the receptor using the Protein Preparation Wizard in the Schrödinger software (version 2024-1). Hydrogen atoms were added, and the structure was energy-minimized. The docking grid was centered on the SAM binding site, defined by the centroid of key binding residues, with a cubic grid of a 15 Å edge, which included the A1408 channel residues W107 and W197. Virtual screening was then carried out using the Glide module^32^ in the Schrödinger software (version 2024-1) in a hierarchical manner. An initial virtual screening of approximately 2 million prepared ligands was first conducted in HTVS mode. The top 10% of compounds (∼130,000) based on HTVS scores was selected for further evaluation using SP docking, and from this set, the top 10% (∼13,000 ligands) was further refined by XP docking to obtain more accurate binding poses. In the XP docking step, up to five binding poses were generated for each ligand to explore alternative interactions. All resulting poses with XP docking scores lower than −6.0 kcal/mol were then retained for subsequent analysis (3,956 ligands, ∼30%). Note that, for clarity, the plot in Figure 2B shows only a representative sample at each stage, corresponding to 230,947 ligands, 14,923 ligands, and 26,738 binding poses for HTVS, SP docking, and XP docking, respectively, but these plotted score distributions closely reflect trends across the full data set. The selected ligands were further analyzed for chemical similarity and K-means clustering, and then, cluster representatives were further evaluated for physicochemical diversity, using the Discovery Informatics suite in Schrödinger, to prioritize compounds for purchase and experimental testing.

MMGBSA binding calculations^34,35^ were performed using the Prime module with the OPLS4 force field, allowing receptor side-chain flexibility within 6 Å of the ligand. All structural figures were generated using PyMOL, which was used to visualize ligand–protein interactions, residue conformations, and binding pocket architecture across NpmA crystal structures and docked poses.

### MD Simulations

The open state of NpmA (extracted from PDB 4OX9) was prepared for MD simulation using the Protein Preparation Wizard, and simulations were carried out using Desmond with the OPLS4 force field^33^ in the Schrödinger software (version 2024-4). *Apo* NpmA and each ligand-bound system (compounds **23**, **45**, and **47**) were simulated in triplicate, with each replicate initiated by using a different set of random initial velocities. Systems were neutralized using sodium ions via the System Builder module and then solvated in a TIP3P water box. To achieve a physiological ionic strength of 150 mM, random water molecules were replaced with Na^+^ and Cl^–^ ions. The system was relaxed by using the Desmond standard relaxation protocol, which consists of five sequential steps. First, energy minimization was performed for 1000 steps with 50 kcal/mol/Å^2^ restraints on all solute heavy atoms. This was followed by a 12 ps NVT simulation with the temperature gradually increasing to 310 K, with 50 kcal/mol/Å^2^ restraints applied on all solute atoms to allow solvent relaxation. Next, a 12 ps NPT simulation was conducted at 1.01325 bar with the same restraints to allow volume equilibration. In the fourth step, a 24 ps NPT simulation was run with restraints applied only to solute heavy atoms. Finally, a 24 ps unrestrained NPT simulation was performed to fully equilibrate the system prior to production dynamics. Subsequently, unrestrained MD simulations were conducted under NPT ensemble conditions and comprised a 10 ns equilibration phase, followed by either a 500 ns (*apo* NpmA) or 100 ns (ligand-bound NpmA) production run. Coordinates were saved every 50 ps for trajectory analysis. Temperature and pressure were maintained using the Nosé-Hoover chain thermostat and Martyna–Tobias–Klein barostat, with relaxation times of 1 and 2 ps, respectively. The equations of motion were integrated using multiple time steps: 2 fs for short-range and 6 fs for long-range interactions, applying a 10 Å cutoff for nonbonded interactions. RMSF values were extracted from production trajectories and averaged across replicates. For analysis of W107-W197 dynamics, the six-membered rings of the indole side chains were used to calculate inter-ring distances and angles using Maestro. Ligand–protein interaction frequencies and time-dependent contacts with surrounding residues were analyzed using the built-in trajectory analysis tools in Desmond.

### *In Vitro* NpmA Methyltransferase Inhibition Assay

Recombinant NpmA was expressed and purified, and 30S subunits were isolated using established protocols.^36–38^ Compounds were dissolved in DMSO to generate 10 mM stocks. NpmA methyltransferase assays (30 μL total volume) included NpmA (0.3 μM), 30S subunit (0.3 μM), [^3^H]-SAM (0.302 μM), and test compounds at final concentrations as noted in the Results and Discussion (1.0, 0.5, or 0.1 mM) in a buffer containing 5 mM HEPES-KOH (pH 7.5), 50 mM KCl, 10 mM NH_4_Cl, 10 mM magnesium acetate, 6 mM β-mercaptoethanol, and 10% (v/v) DMSO. Reaction mixtures were incubated at 37 °C for 10 min before quantification of methylation activity (i.e., ^3^H incorporation into 30S subunits) using a filter binding assay. Reaction mixtures were transferred to the wells of a glass fiber filter 96-well plate, which were then washed thoroughly to remove unincorporated [^3^H]-SAM and dried, and retained radioactivity was measured by liquid scintillation counting.

Dose–response curves (IC_50_ measurement) for select compounds were generated using the same assay but with a half-log dilution series of test compound (1000 to 7.81 μM). Each assay was performed in at least duplicate. IC_50_ values were calculated by fitting the inhibition data to a four-parameter logistic regression model using GraphPad Prism 10.

### Chemical Synthesis of **23**

The complete synthetic route to **23** is summarized in Figure S5.

#### **50**, 2-(((Benzyloxy)­carbonyl)­amino)-2-(dimethoxyphosphoryl)­acetic Acid

An aqueous solution of NaOH (0.12 g, 1.51 mL, 2.00 molar, 1.0 equiv, 3.02 mmol) was added to a solution of **49** (methyl 2-(((benzyloxy)­carbonyl)­amino)-2-(dimethoxyphosphoryl)­acetate) (1.00 g, 1 equiv, 3.0 mmol) in 1,4-dioxane (3.0 mL). The mixture was stirred at room temperature for 1.5 h and then acidified with 5 N HCl and extracted with ethyl acetate (EtOAc). The combined organic layers were dried over Na_2_SO_4_, and solvent was removed *in vacuo*. The residue was taken up in hot EtOAc (∼5 mL) and allowed to stand for 2 d, and the resulting crystals were removed by filtration and washed 3×x with cold EtOAc. The filtrate was concentrated to afford the title compound (926 mg, 2.92 mmol, 97%) as a viscous oil. Product characterization by ^1^H and ^13^C NMR and high-resolution mass spectrometry (HRMS) is provided in the Supplementary Methods.

#### **51**, Benzyl (2-((4-Acetylphenyl)­amino)-1-(dimethoxyphosphoryl)-2-oxoethyl)­carbamate

DIPEA (3.43 mL, 2.5 equiv, 19.7 mmol) and HATU (4.50 g, 1.5 equiv, 11.8 mmol) were added to a stirring solution of **50** (2.50 g, 1 equiv, 7.88 mmol) and 4′-aminoacetophenone (1.28 g, 1.2 equiv, 9.46 mmol) in MeCN (20 mL). The mixture was stirred at room temperature overnight and then concentrated. The residue was partitioned between EtOAc and saturated aqueous NH_4_Cl, and the aqueous layer was extracted with EtOAc. The combined organic layers were washed with 1 N HCl and brine, dried over Na_2_SO_4_, and concentrated. The crude residue was purified by flash chromatography (0–10% MeOH/DCM) to afford the title compound (1.86 g, 4.28 mmol, 54%) as a tan solid. Product characterization by ^1^H and ^13^C NMR and HRMS is provided in the Supplementary Methods.

#### **53**, Dimethyl (2-((4-Acetylphenyl)­amino)-1-amino-2-oxoethyl)­phosphonate

A solution of **51** (1.00 g, 1 equiv, 2.30 mmol) in 2:1 MeOH/EtOAc was thoroughly degassed by sparging with Ar. Pd/C (100 mg, 5 wt % Pd loading) was added, and the reaction vessel was evacuated and purged 3× with H_2_. The mixture was stirred for 3 h at room temperature; then, the atmosphere was exchanged for Ar, the vessel was opened to air, and the mixture was diluted with EtOAc. The crude product **52** was isolated by careful filtration through Celite and removal of volatiles under reduced pressure and used directly without purification.

A solution of the deprotected intermediate and 2-chlorobenzoic acid (432 mg, 1.2 equiv, 2.76 mmol) in MeCN (8.0 mL) was treated with HATU (1.31 g, 1.5 equiv, 3.45 mmol) and DIPEA (1.00 mL, 2.5 equiv, 5.75 mmol). The reaction mixture was stirred at room temperature overnight. Saturated aqueous NH_4_Cl was added, and the mixture was partitioned with EtOAc. The aqueous layer was extracted with EtOAc, and the combined organic layers were washed with 1 N HCl and brine, dried over Na_2_SO_4_, and concentrated. The residue was purified by flash column chromatography (50 g, 0–10% *i*PrOH/DCM) to afford the title compound (620 mg, 1.41 mmol, 61%) as a tan solid. Product characterization by ^1^H and ^13^C NMR and HRMS is provided in the Supplementary Methods.

#### **23**, (*Z*)-*N*-(3-((4-Acetylphenyl)­amino)-1-(3-nitrophenyl)-3-oxoprop-1-en-2-yl)-2-chlorobenz-amide

A reaction tube was charged with LiCl (29 mg, 4 equiv, 680 μmol), flame-dried under vacuum, and then flushed with Ar. A solution of **53** (75 mg, 1 Eq, 170 μmol) in DMF (460 μL) was added, and the mixture was cooled to 0 °C. A solution of triethylamine (140 μL, 2 M, 1.2 equiv, 270 μmol) in DMF was then added dropwise. *N*-Boc 3-formylazetidine-1-carboxylate (140 μL, 2 M, 1.2 equiv, 270 μmol) was added as a solution in DCM, and the reaction was stirred at 0 °C for 3 h. Saturated aqueous NH_4_Cl was added, and the mixture was allowed to warm to room temperature; then, the aqueous layer was extracted with EtOAc. The combined organic layers were washed with brine, dried in Na_2_SO_4_, and concentrated. The crude product was purified by flash chromatography (25 g, 0–50% acetone/hexanes) to afford the title compound (20 mg, 43 μmol, 25%) as a white solid. Product characterization by ^1^H and ^13^C NMR and HRMS is provided in the Supplementary Methods.

## Supplementary Material



## Notes

Figures S1–S7: MD simulation, chemical structures, synthesis and characterization of compounds, SAM/A1408 binding pocket and compatibility with ligand (*E*)/(*Z*) stereoisomers of compound **3**, initial SAR insight from ligand docking, ligand contacts in replicate MD runs for compound **23**; Tables S1–S2: ligand docking scores with NpmA and MMGBSA binding energy components for selected compounds; NMR and HRMS analysis (PDF)
