# Supplementary material for: Discovery of First-in-Class Inhibitors Targeting a Pathogen-Associated Aminoglycoside-Resistance 16S rRNA Methyltransferase
Source: ACS Infect Dis. 2025 Jul 11;11(8):2276–86. doi: 10.1021/acsinfecdis.5c00297 (PMC12340973; doi:10.1021/acsinfecdis.5c00297)
Supplement: Supplementary file 1 [file id5c00297_si_001.pdf]

Supporting Information

Discovery of First-in-Class Inhibitors Targeting a Pathogen-Associated Aminoglycoside-Resistance 16S rRNA Methyltransferase

Debayan Dey[1*], Benjamin E. Deprez[2], Natalia Zelinskaya[1], Jose M Castro[1,3], William M. Wuest[2]*, Graeme L Conn[1]*

[1]Department of Biochemistry, Emory University School of Medicine, Atlanta, GA 30322, USA.

[2]Department of Chemistry, Emory University, Atlanta, GA 30322, USA.

[3]Graduate Program in Biochemistry, Cell and Developmental Biology (BCDB), Emory University, Atlanta, GA 30322, USA

*To whom correspondence should be addressed: Graeme L Conn, email: gconn@emory.edu; William M. Wuest, email: wwuest@emory.edu

Supplemental Figures

Figure S1. Trp107/Trp197 adopt orientations corresponding to both open and closed SAM/A1408 binding pockets in MD simulation.

Figure S2. Chemical structures of the initial diverse set of compounds **1-22**.

Figure S3. The SAM/A1408 binding pocket and compatibility with ligand (*E*)/(*Z*) stereoisomers of compound **3**.

Figure S4. Chemical structures of compounds **23-48**.

Figure S5. Synthesis and characterization of compound **23**.

Figure S6. Initial SAR insight from ligand docking.

Figure S7. Ligand contacts in replicate MD runs for compound **23** (*Z*).

Supplemental Tables

Table S1. Table S1. Ligand docking scores with NpmA.

Table S2. MMGBSA binding energy components for selected compounds.

Supplemental Methods

^{1}H and ^{13}C nuclear magnetic resonance (NMR) and high-resolution mass spectrometry (HRMS) characterization of 23 and its synthesis intermediates.

^{1}H and ^{13}C NMR Characterization of select compounds purchased from ChemBridge.

NMR Spectra.

Supplemental Figures

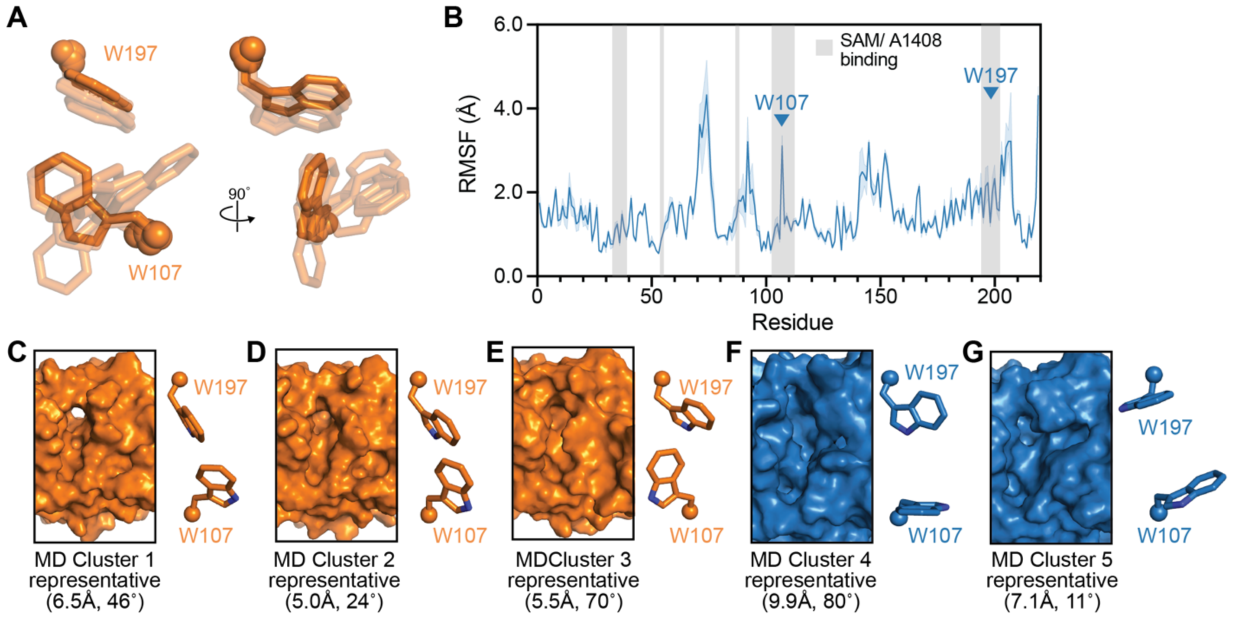

Figure S1. Trp107/Trp197 adopt orientations corresponding to both open and closed SAM/A1408 binding pockets in MD simulation. A, Variability in Trp107/Trp197 relative orientation in crystal structures of free NpmA (taken from all chains in PDBs 3MTE, 3P2I, 3P2A, 3PB3, and 3P2K). The solid sticks are PDB 3MTE chain A which is highlighted in main **Figure 1**. **B**, NpmA residue root mean square fluctuation (RSMF) during the 500 ns MD simulations. The locations of Trp107/Trp197 and sites of NpmA interaction with SAM or A1408 are highlighted. **C**-**G**, Representative structures from the MD simulation after clustering, showing the presence of both closed (orange) and open (blue) SAM/A1408 binding pockets. The Trp107/Trp197 distance and angle values used to designate the open or closed state are shown below (also see **Figure 1C,D**).

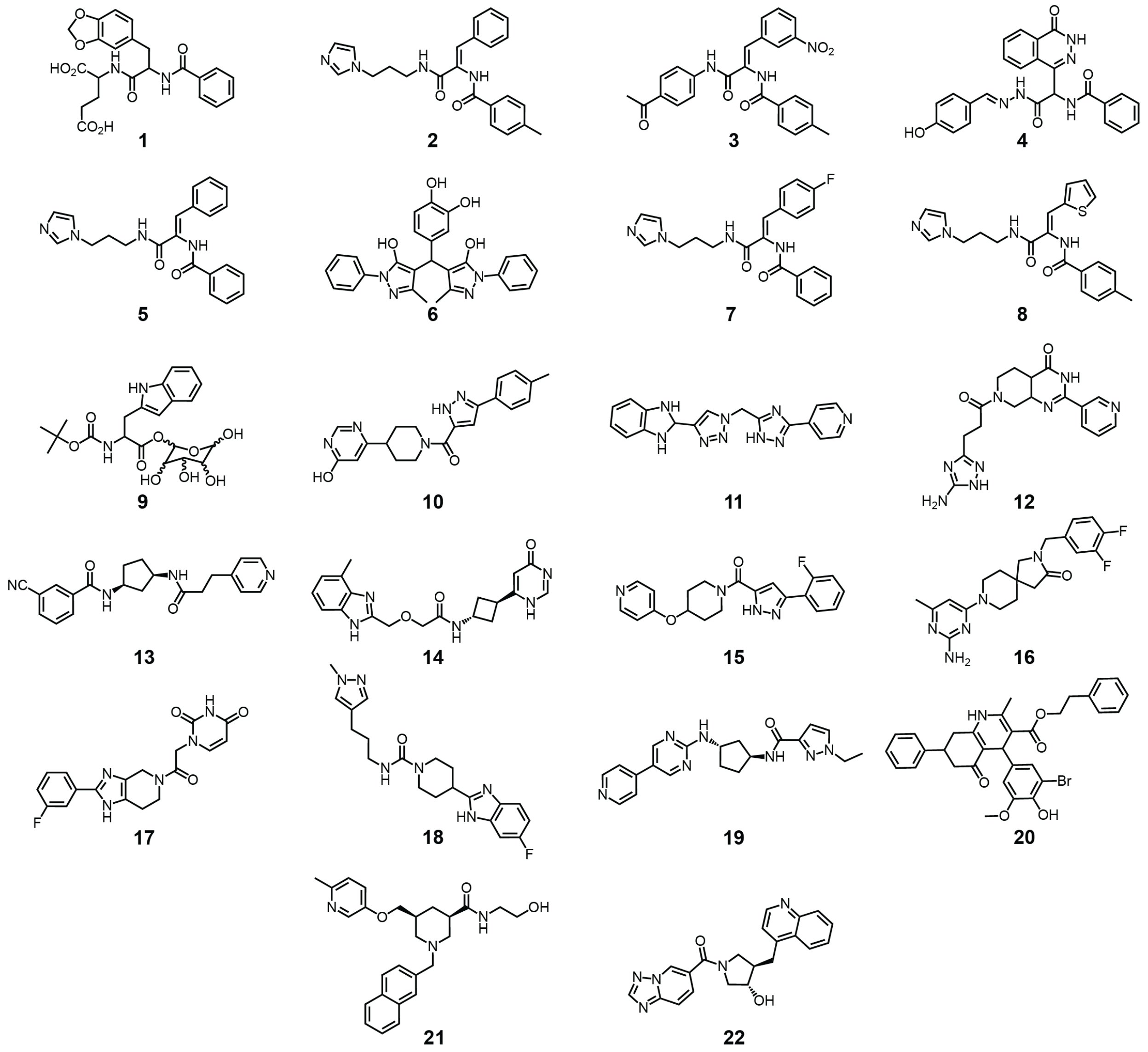

Figure S2. Chemical structures of the initial diverse set of ChemBridge compounds (1-22).

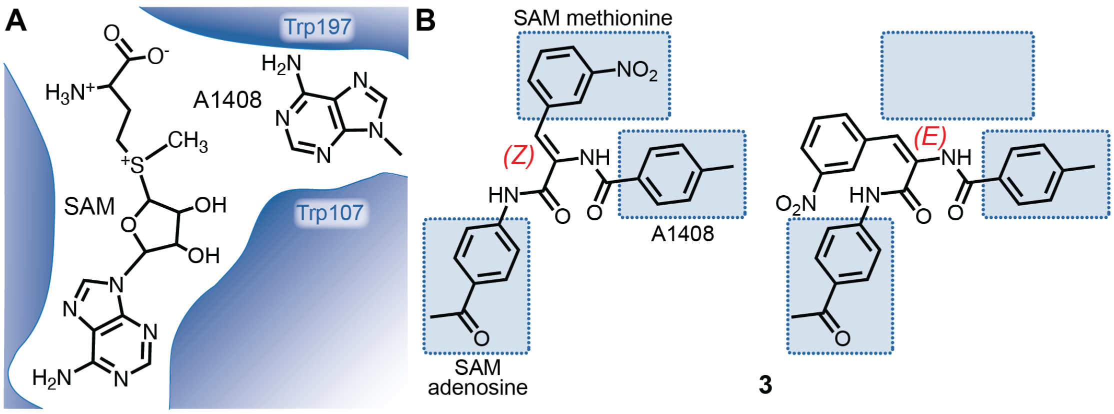

Figure S3. The SAM/A1408 binding pocket and compatibility with ligand (*E*)/(*Z*) stereoisomers of compound 3. A, SAM and A1408 shown within the Y-shaped binding pocket of the 30S-bound confirmation of NpmA. **B**, Comparison of **3** (*Z*) and **3** (*E*) with three identified "zones" for ligand modification indicated with their corresponding location in the SAM/A1408 binding pocket.

A

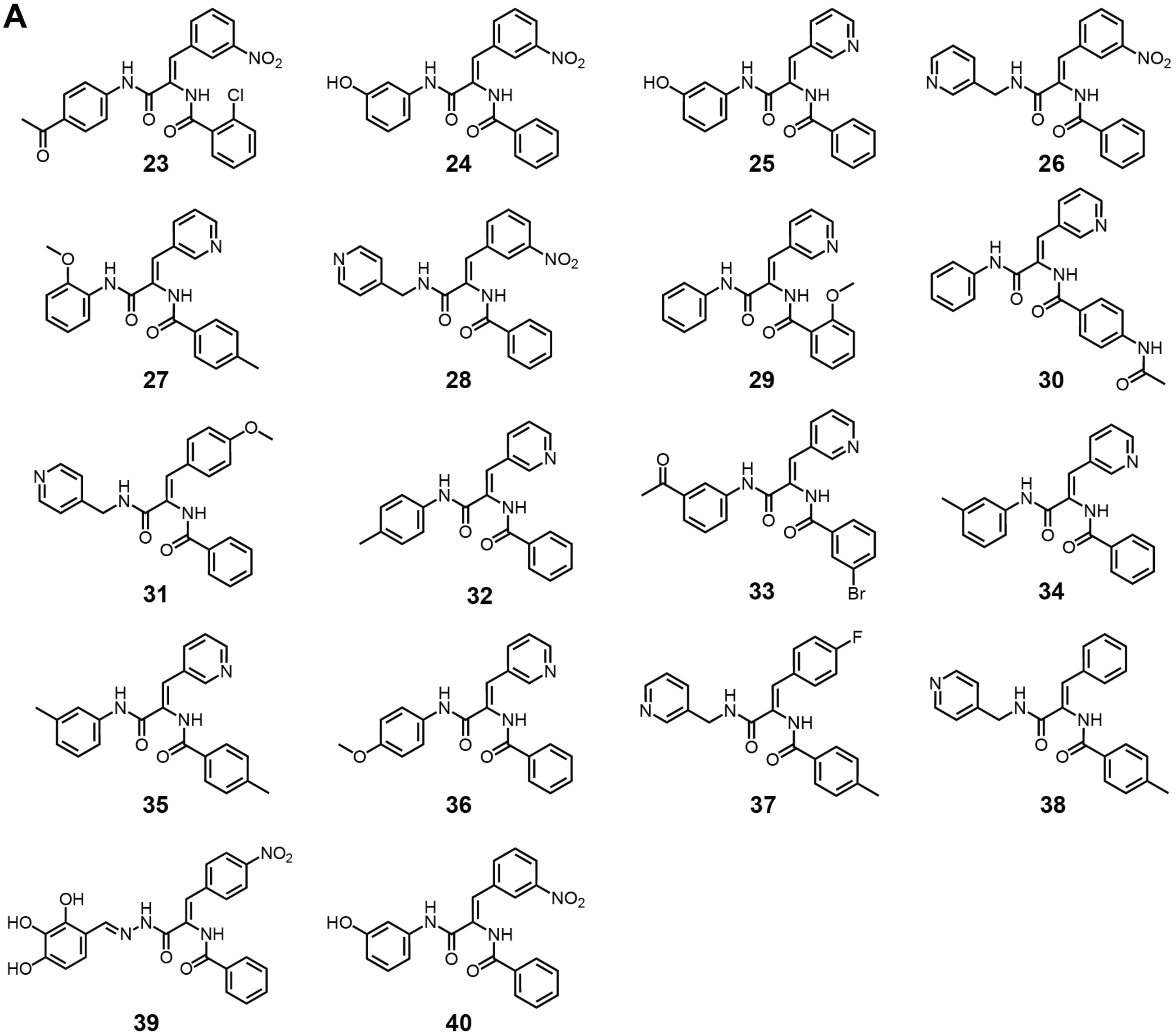

B

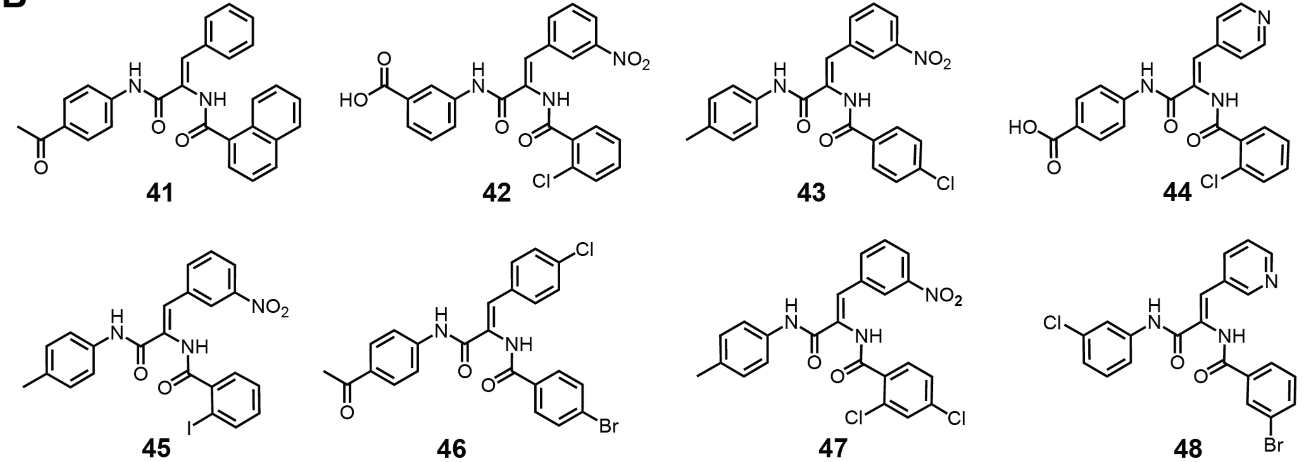

Figure S4. Chemical structures of analogs of 3. A, Compounds 23-40 and **B**, compounds 41-48 (most similar to 23) from ChemBridge.

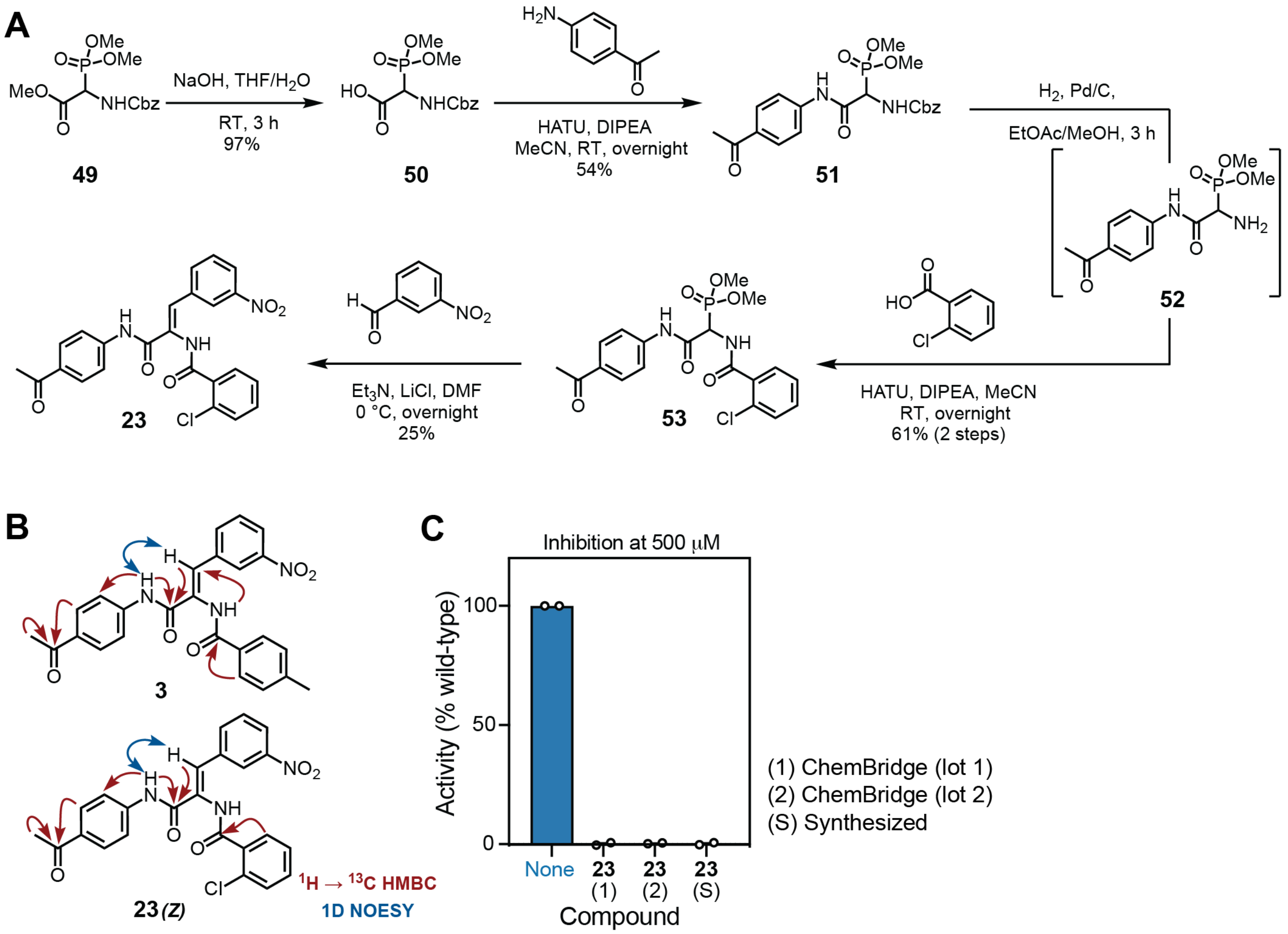

Figure S5. Synthesis and characterization of compound 23 (Z). A, Synthetic route to **23** (see main text for details). **B**, $^{1}H \rightarrow ^{13}C$ Heteronuclear Multiple Bond Correlation (HMBC; red) and 1D selective Nuclear Overhauser Effect Spectroscopy (NOESY; blue) NMR correlations used for unambiguous assignment of the central olefin geometry in **3** and **23**. **C**, Fixed concentration (500 μM **23**) inhibition of NpmA methyltransferase activity comparing **23** purchased from ChemBridge (two different lots) and synthesized via the scheme shown in *panel A*.

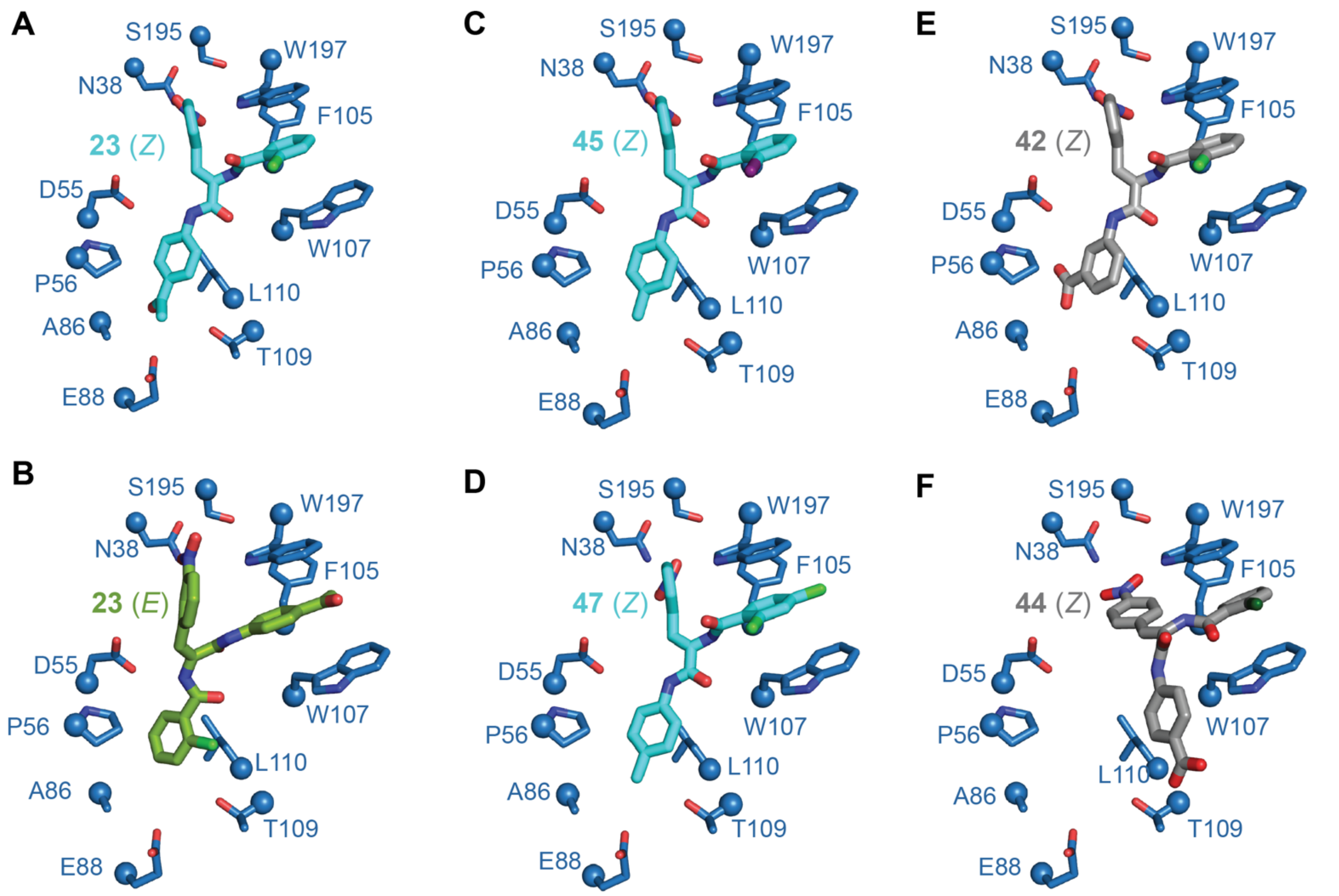

Figure S6. Initial SAR insight from ligand docking. Views of the ligand binding pocket with select docked compounds. **A**, **23** (*Z*) and **B**, **23** (*E*), highlighting the impact of stereochemistry on the positioning of the arene rings in the SAM adenine (*bottom center*) and A1408 channel (*top right*), while that in the SAM methionine region (*top center*) is fixed. Two close analogs of **23** with similar NpmA inhibition activity, **C**, **45** and **D**, **47**. Two close analogs of **23** with lower NpmA inhibition activity, **E**, **42** and **F**, **44**.

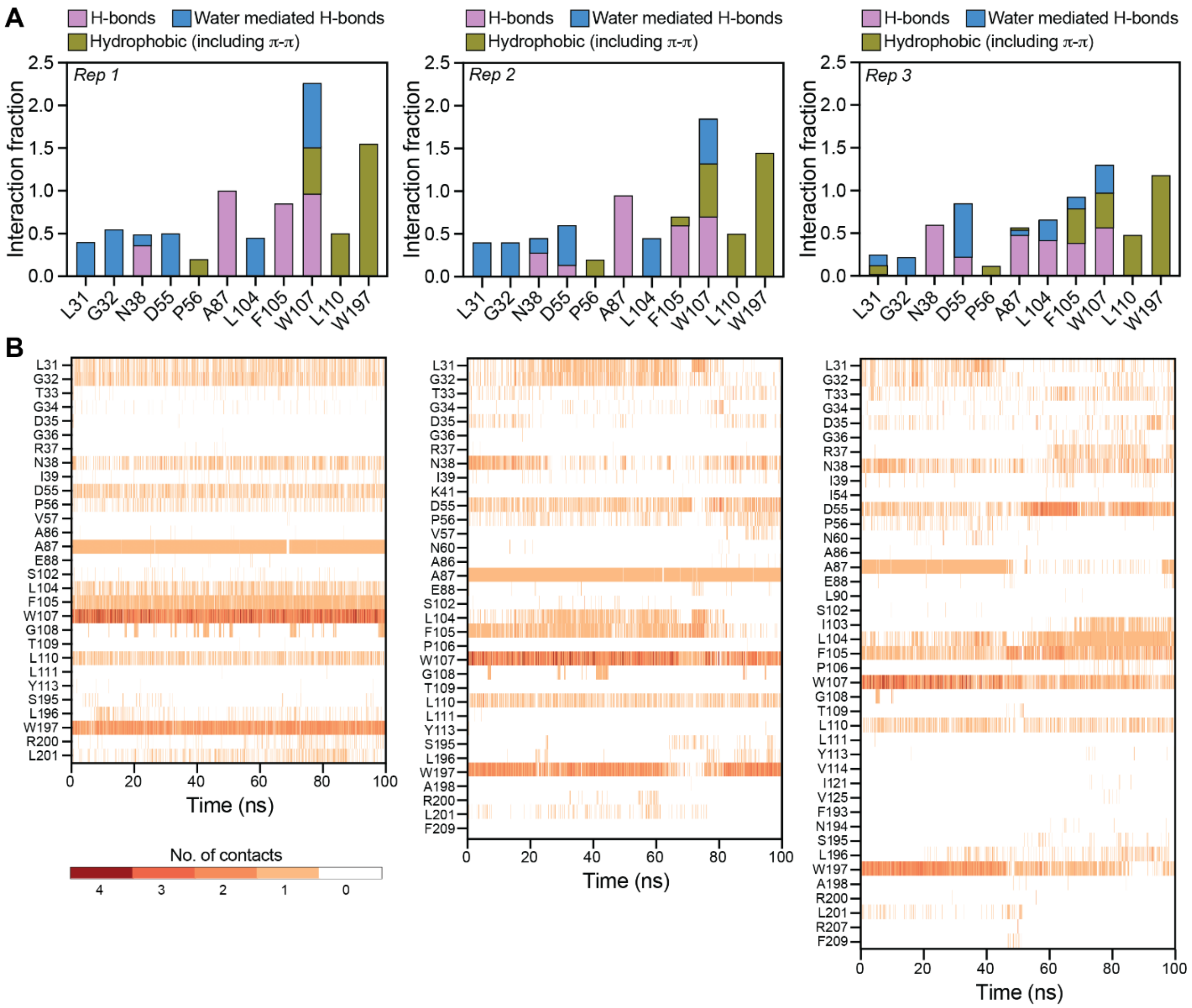

Figure S7. Ligand contacts in replicate MD runs for compound 23 (*Z*). A, Summary of interaction types made with key contact residues in the SAM/A1408 binding pocket in the three replicate simulations of NpmA-**23**. Data for replicate 1 (Rep 1) are the same as those shown in **Figure 5B**. **B**, Stability of contacts during the replicate 100 ns MD simulations.

Supplemental Tables

Table S1. Ligand docking scores with NpmA

Compound #	Glide XP Docking Score (kcal/mol)	
1	-7.9	
2	-8.6	
**3**	**-9.9 (*Z*)**	
4	-7.8	
5	-10.3	
6	-6.8	
7	-10.4	
8	-10.6	
9	-8.9	
**10**	**-8.9**	
11	-9.5	
12	-7.6	
13	-11.6	
14	-8.9	
15	-9.8	
16	-9.8	
17	-8.0	
18	-8.3	
19	-8.9	
**20**	**-7.2**	
21	-9.9	
22	-9.3	
	(*Z*) stereoisomer	(*E*) stereoisomer
**23**	**-9.9**	**-6.1**
24	-8.3	-6.5
25	-9.1	-6.1
26	-5.6	-6.5
27	-6.9	ND
28	-7.4	ND
29	ND	-7.4
30	ND	-4.9
31	-7.0	ND
32	ND	-6.6
33	-10.2	ND
34	-7.3	ND
35	ND	-6.8
36	ND	-7.1
37	-7.7	ND
38	-7.4	ND
39	-9.5	ND
40	-8.3	ND
41	-4.8	-10.3
42	-5.6	0.2
43	-8.0	-5.6
44	-5.5	-6.3
**45**	**-7.9**	**-4.0**
46	-7.6	ND
**47**	**-8.1**	**-4.7**
48	-7.7	-5.5

Table S2. MMGBSA binding energy components for selected compounds

Compound	XP Score	MMGBSA ΔG Bind	MMGBSA ΔG Bind Coulomb	MMGBSA ΔG Bind Hbond	MMGBSA ΔG Bind Lipo	MMGBSA ΔG Bind Packing	MMGBSA ΔG Bind Solv GB	MMGBSA ΔG Bind vdW
3 (*Z*)	-9.9	-99.7	-22.4	-2.1	-32.8	-8.3	28.6	-62.8
23 (*Z*)	-9.9	-104.8	-27.8	-3.1	-32.3	-8.2	29.0	-62.4
45 (*Z*)	-7.9	-95.6	-21.4	-2.4	-31.4	-7.8	28.6	-61.3
46 (*Z*)	-7.6	-91.6	-26.2	-1.7	-30.2	-9.3	33.5	-57.7
47 (*Z*)	-8.1	-96.5	-16.0	-1.7	-36.0	-7.5	29.2	-64.5
48 (*Z*)	-7.7	-88.5	-23.6	-0.6	-31.7	-8.1	30.2	-54.6

Supplemental Methods

^{1}H and ^{13}C nuclear magnetic resonance (NMR) and high-resolution mass spectrometry (HRMS) characterization of 23 and its synthesis intermediates

[**50**] *2-(((benzyloxy)carbonyl)amino)-2-(dimethoxyphosphoryl)acetic acid*–^{1}H NMR (400 MHz, DMSO) δ 13.37 (s, 1H), 8.15 (dd, *J* = 9.6, 2.5 Hz, 1H), 7.42 – 7.22 (m, 5H), 5.08 (d, *J* = 1.9 Hz, 2H), 4.70 (dd, *J* = 24.1, 9.5 Hz, 1H), 3.70 (d, *J* = 8.7 Hz, 3H), 3.67 (d, *J* = 8.7 Hz, 3H). ^{13}C NMR (101 MHz, DMSO) δ 167.97, 167.93, 156.29, 156.20, 136.81, 128.42, 127.95, 127.77, 66.41, 66.03, 53.65, 53.58, 52.84, 51.37. HRMS (ESI-): calculated for $C_{12}H_{15}O_7NP$ [M-H]$^-$ 316.0592, found 316.0587.

[**51**] *Benzyl (2-((4-acetylphenyl)amino)-1-(dimethoxyphosphoryl)-2-oxoethyl)carbamate*–^{1}H NMR (400 MHz, DMSO) δ 10.61 (s, 1H), 8.11 – 8.04 (m, 1H), 7.96 (d, *J* = 8.4 Hz, 2H), 7.72 (d, *J* = 8.3 Hz, 2H), 7.43 – 7.25 (m, 5H), 5.15 – 4.97 (m, 3H), 3.72 (s, 3H), 3.70 (s, 3H), 2.53 (s, 3H). ^{13}C NMR (101 MHz, DMSO) δ 196.58, 164.83, 164.80, 156.09, 156.00, 142.63, 136.64, 132.27, 129.61, 128.38, 127.93, 127.76, 118.64, 66.17, 53.84, 53.78, 53.60, 53.54, 52.68, 26.49. HRMS (APCI–): calculated for $C_{20}H_{22}O_7N_2P$ [M–H]$^-$ 433.11701, found 433.1160.

[**53**] *Dimethyl (2-((4-acetylphenyl)amino)-1-amino-2-oxoethyl)phosphonate*–^{1}H NMR (400 MHz, DMSO) δ 10.71 (s, 1H), 9.28 (dd, *J* = 9.1, 2.6 Hz, 1H), 8.01 – 7.94 (m, 2H), 7.82 – 7.67 (m, 2H), 7.59 – 7.33 (m, 4H), 5.58 (dd, *J* = 21.2, 9.1 Hz, 1H), 3.77 (d, *J* = 5.0 Hz, 3H), 3.75 (d, *J* = 4.8 Hz, 3H), 2.54 (s, 3H). ^{13}C NMR (101 MHz, DMSO) δ 196.56, 166.40, 166.33, 164.24, 164.22, 142.67, 135.59, 132.28, 131.12, 130.10, 129.63, 129.53, 129.34, 126.92, 118.63, 53.95, 53.89, 53.66, 53.60, 52.25, 50.83, 26.49. HRMS (APCI+): calculated for $C_{20}H_{17}O_2N_6{}^{35}ClP$ [M+H]$^+$ 439.08203, found 439.08235.

[**23**] *(Z)-N-(3-((4-acetylphenyl)amino)-1-(3-nitrophenyl)-3-oxoprop-1-en-2-yl)-2-chlorobenz-amide*–^{1}H NMR (400 MHz, DMSO) δ 10.71 (s, 1H), 10.48 (s, 1H), 8.58 (t, *J* = 2.0 Hz, 1H), 8.21 (ddd, *J* = 8.2, 2.6, 1.0 Hz, 1H), 8.08 (d, *J* = 7.8 Hz, 1H), 8.00 (d, *J* = 8.8 Hz, 2H), 7.91 (d, *J* = 8.8 Hz, 2H), 7.74 (t, *J* = 8.0 Hz, 1H), 7.59 (ddd, *J* = 11.1, 7.5, 1.7 Hz, 2H), 7.56 – 7.43 (m, 2H), 7.19 (s, 1H), 2.57 (s, 3H). ^{13}C NMR (151 MHz, DMSO) δ 196.60, 166.19, 164.09, 147.89, 143.59, 136.01, 135.75, 135.54, 132.52, 131.93, 131.42, 130.06, 130.05 129.79, 129.40, 129.01, 127.13, 124.97, 123.53, 123.11, 118.99, 26.48. NMR data match that of the purchased sample. HRMS (APCI–): calculated for $C_{24}H_{17}O_5N_3{}^{35}Cl$ [M–H]$^-$ 462.08622, found 462.08617.

^{1}H and ^{13}C NMR Characterization of select compounds purchased from ChemBridge

[3] (Z)-N-(3-((4-acetylphenyl)amino)-1-(3-nitrophenyl)-3-oxoprop-1-en-2-yl)-4-methylbenz-amide–^{1}H NMR (800 MHz, DMSO) δ 10.65 (d, *J* = 1.7 Hz, 1H), 10.26 (s, 1H), 8.54 (t, *J* = 2.0 Hz, 1H), 8.18 (ddd, *J* = 8.2, 2.4, 1.0 Hz, 1H), 8.04 (dt, *J* = 7.9, 1.3 Hz, 1H), 7.98 – 7.95 (m, 2H), 7.91 (d, *J* = 8.0 Hz, 2H), 7.89 (dd, *J* = 8.7, 3.1 Hz, 2H), 7.71 (t, *J* = 8.0 Hz, 1H), 7.34 (d, *J* = 7.9 Hz, 2H), 7.23 (s, 1H), 2.55 (s, 3H), 2.39 (s, 3H). ^{13}C NMR (201 MHz, DMSO) δ 196.59, 165.98, 164.52, 147.81, 143.62, 142.14, 136.09, 135.82, 133.23, 131.90, 130.34, 130.10, 129.30, 128.98, 127.94, 125.23, 123.51, 122.98, 119.13, 26.46, 21.04.

[**33**] *(Z)-N-(3-((3-acetylphenyl)amino)-3-oxo-1-(pyridin-3-yl)prop-1-en-2-yl)-3-bromobenz-amide*–^{1}H NMR (800 MHz, DMSO) δ 10.43 (d, *J* = 1.9 Hz, 1H), 10.35 (s, 1H), 8.77 (d, *J* = 2.2 Hz, 1H), 8.50 (dd, *J* = 4.8, 1.6 Hz, 1H), 8.30 (dt, *J* = 5.2, 2.0 Hz, 1H), 8.22 (t, *J* = 1.8 Hz, 1H), 8.02 – 8.00 (m, 2H), 7.99 (dd, *J* = 3.9, 1.9 Hz, 1H), 7.83 (ddd, *J* = 8.0, 2.0, 1.0 Hz, 1H), 7.71 (ddd, *J* = 7.7, 1.7, 1.0 Hz, 1H), 7.52 (t, *J* = 7.9 Hz, 1H), 7.50 (t, *J* = 7.9 Hz, 1H), 7.44 (ddd, *J* = 8.0, 4.8, 0.9 Hz, 1H), 7.22 (s, 1H), 2.57 (s, 3H). ^{13}C NMR (201 MHz, DMSO) δ 197.66, 164.65, 164.65, 163.95, 150.22, 150.22, 149.24, 149.24, 139.47, 137.20, 136.10, 135.38, 135.38, 134.69, 134.69, 132.24, 132.24, 130.73, 130.63, 130.23, 130.23, 129.02, 129.02, 127.08, 127.08, 125.24, 125.24, 124.63, 124.63, 124.53, 123.70, 123.70, 123.69, 123.69, 121.73, 119.38, 119.38, 119.28, 26.76, 26.76.

[**34**] (Z)-N-(3-oxo-1-(pyridin-3-yl)-3-(m-tolylamino)prop-1-en-2-yl)benzamide–^{1}H NMR (800 MHz, DMSO) δ 10.20 (s, 1H), 10.17 (d, *J* = 2.1 Hz, 1H), 8.78 (d, *J* = 2.2 Hz, 1H), 8.49 (dd, *J* = 4.8, 1.7 Hz, 1H), 8.01 (ddd, *J* = 10.1, 6.0, 1.8 Hz, 3H), 7.64 – 7.60 (m, 1H), 7.57 (q, *J* = 2.4 Hz, 1H), 7.56 – 7.52 (m, 3H), 7.43 (ddd, *J* = 8.0, 4.8, 0.9 Hz, 1H), 7.22 (t, *J* = 7.8 Hz, 1H), 7.14 (s, 1H), 6.91 (ddt, *J* = 7.5, 1.7, 0.9 Hz, 1H), 2.30 (s, 3H). ^{13}C NMR (201 MHz, DMSO) δ 166.44, 164.38, 150.70, 149.51, 139.57, 138.15, 136.48, 133.77, 133.39, 132.40, 130.92, 128.89, 128.86, 128.39, 125.00, 124.68, 124.10, 121.08, 117.76, 21.68.

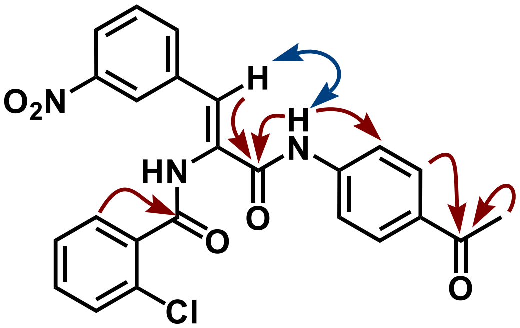

23 *(Z)*

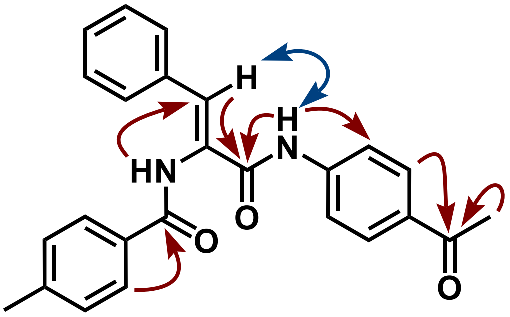

3

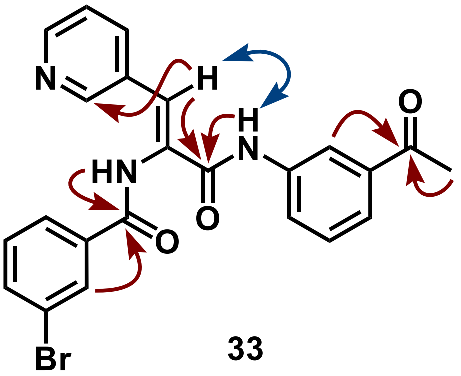

33

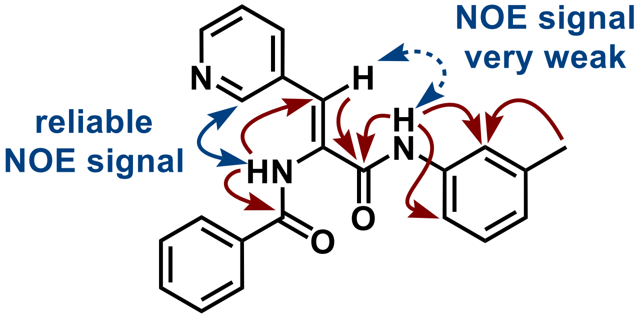

34

$^{1}H \rightarrow {}^{13}C$ HMBC

1D NOESY

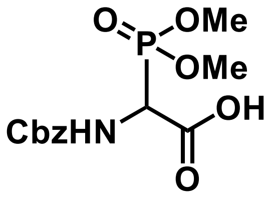
O
P
OMe
OMe
CbzHN
OH
O

50

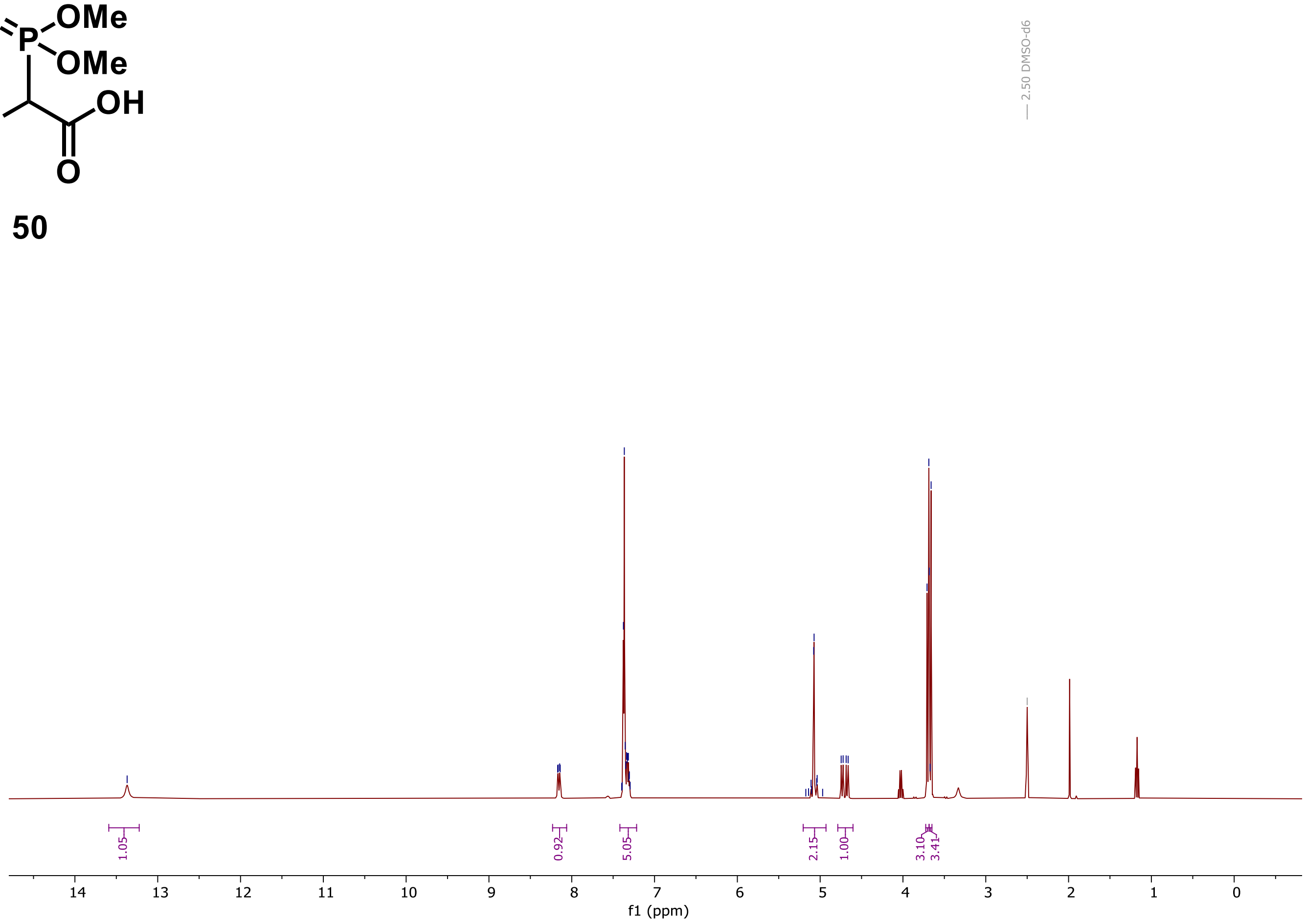
2.50 DMSO-d6
1.05
0.92
5.05
2.15
1.00
3.10
3.41
14 13 12 11 10 9 8 7 6 5 4 3 2 1 0
f1 (ppm)

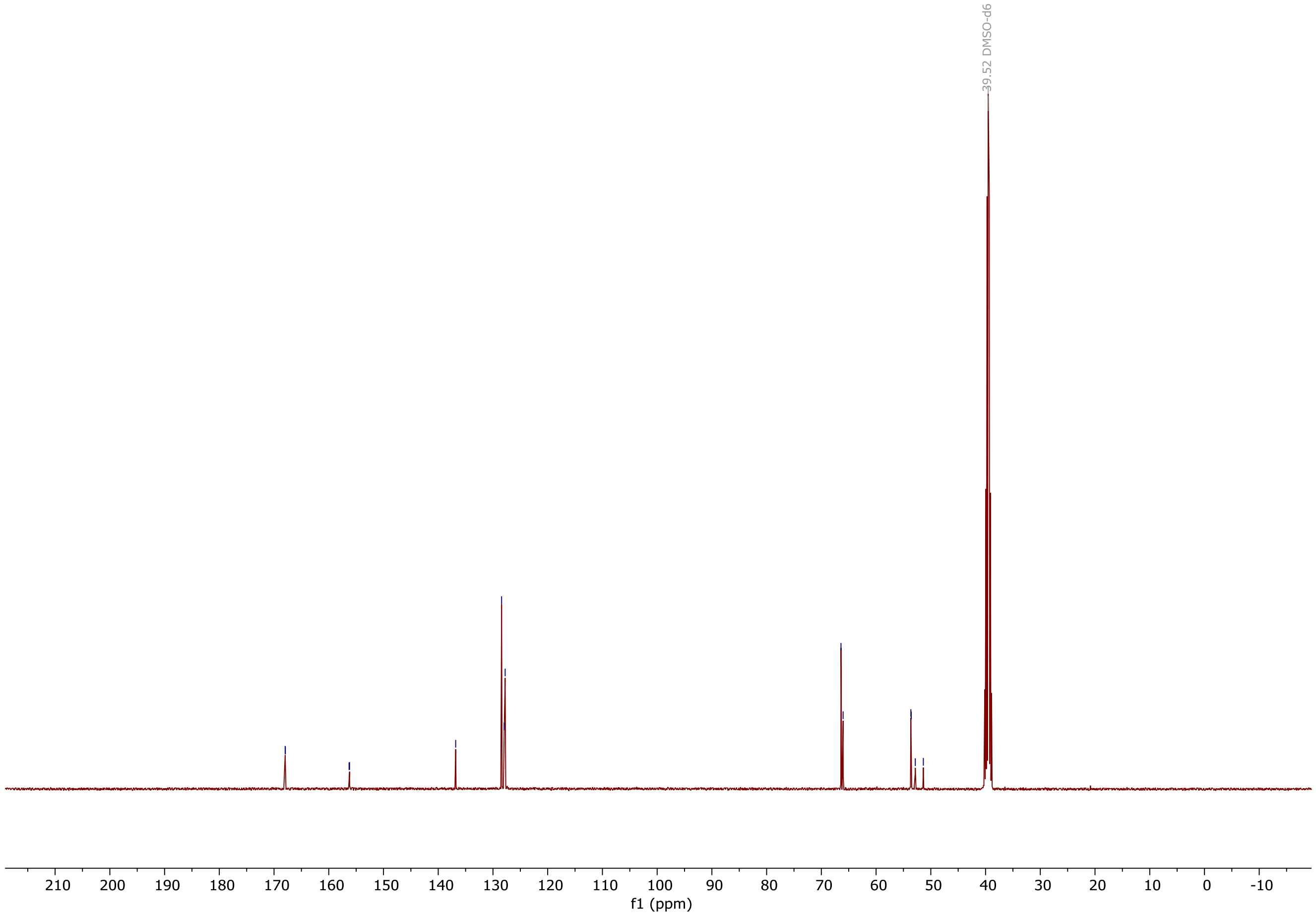
39.52 DMSO-d6
210 200 190 180 170 160 150 140 130 120 110 100 90 80 70 60 50 40 30 20 10 0 -10
f1 (ppm)

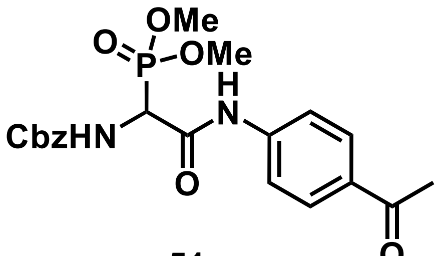
OMe
O
P
OMe
H
N
CbzHN
O
O

51

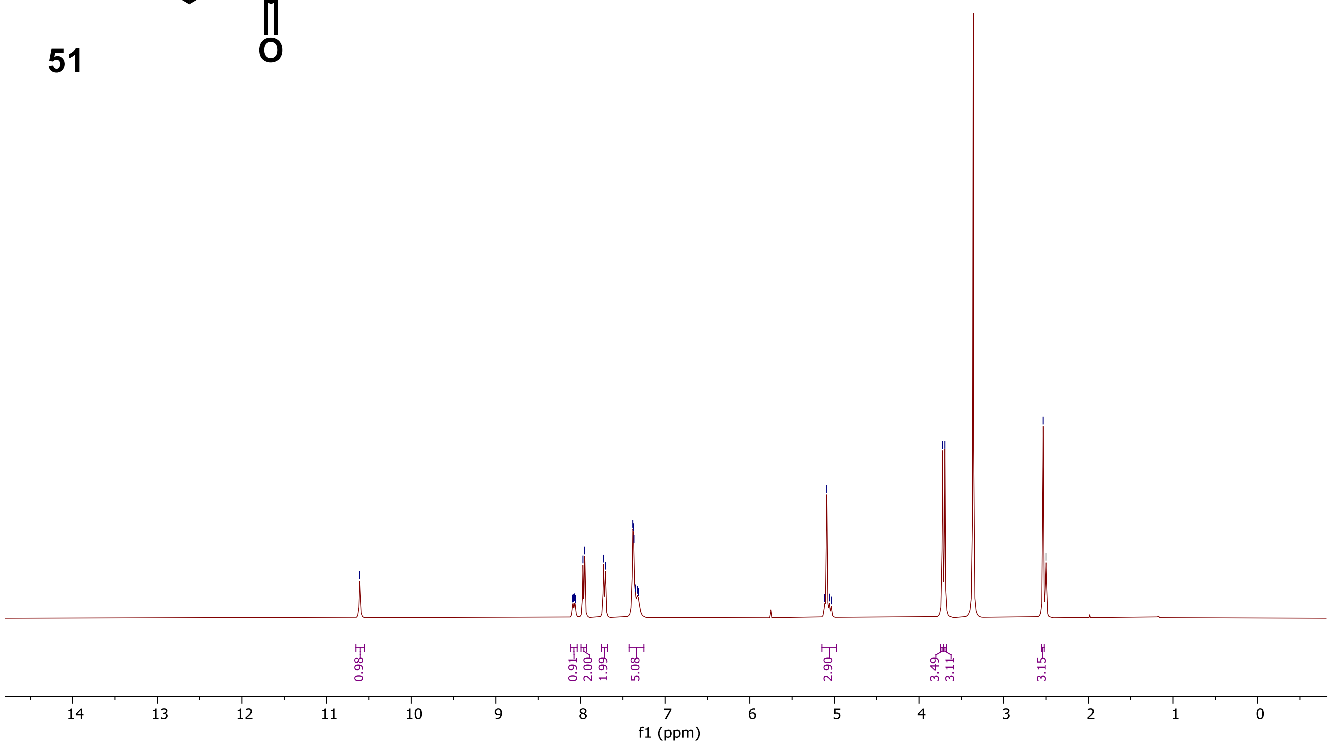
2.50 DMSO-d6
0.98
0.91
2.00
1.99
5.08
2.90
3.49
3.11
3.15
14 13 12 11 10 9 8 7 6 5 4 3 2 1 0
f1 (ppm)

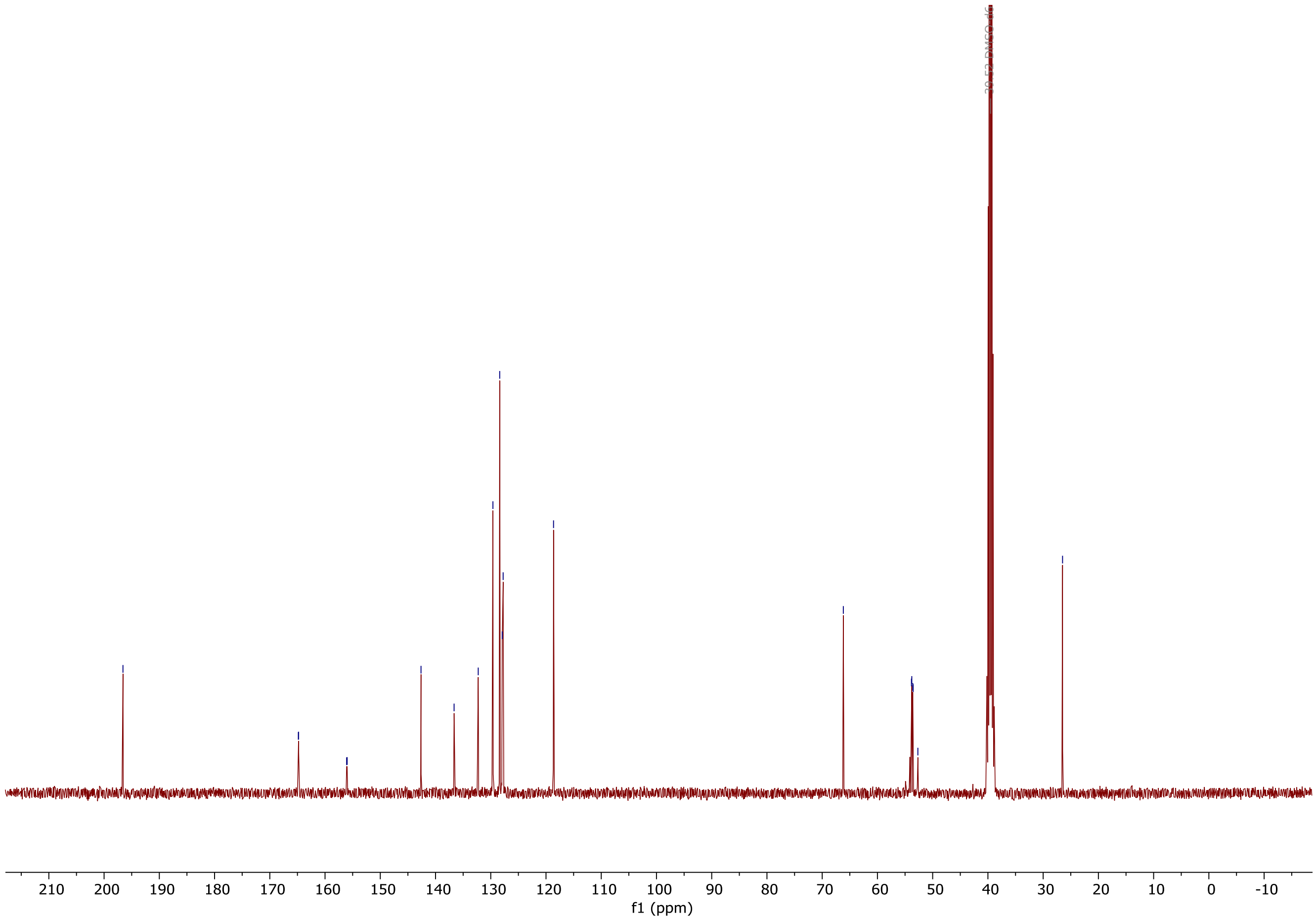
210 200 190 180 170 160 150 140 130 120 110 100 90 80 70 60 50 40 30 20 10 0 -10
f1 (ppm)

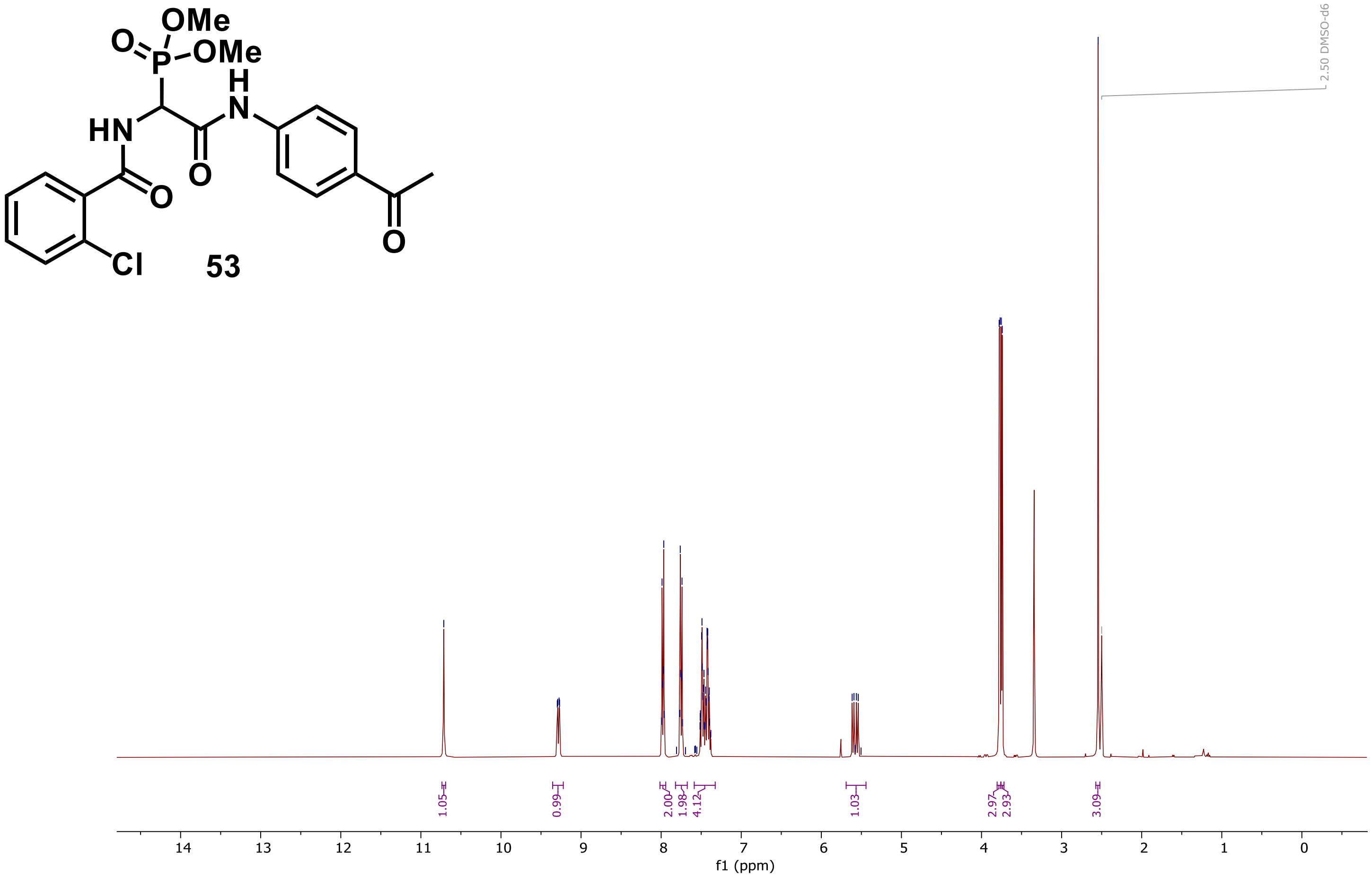
OMe
O
P
OMe
H
N
HN
O
O
Cl
O
53
2.50 DMSO-d6
1.05
0.99
2.00
1.98
4.12
1.03
2.97
2.93
3.09
14 13 12 11 10 9 8 7 6 5 4 3 2 1 0
f1 (ppm)

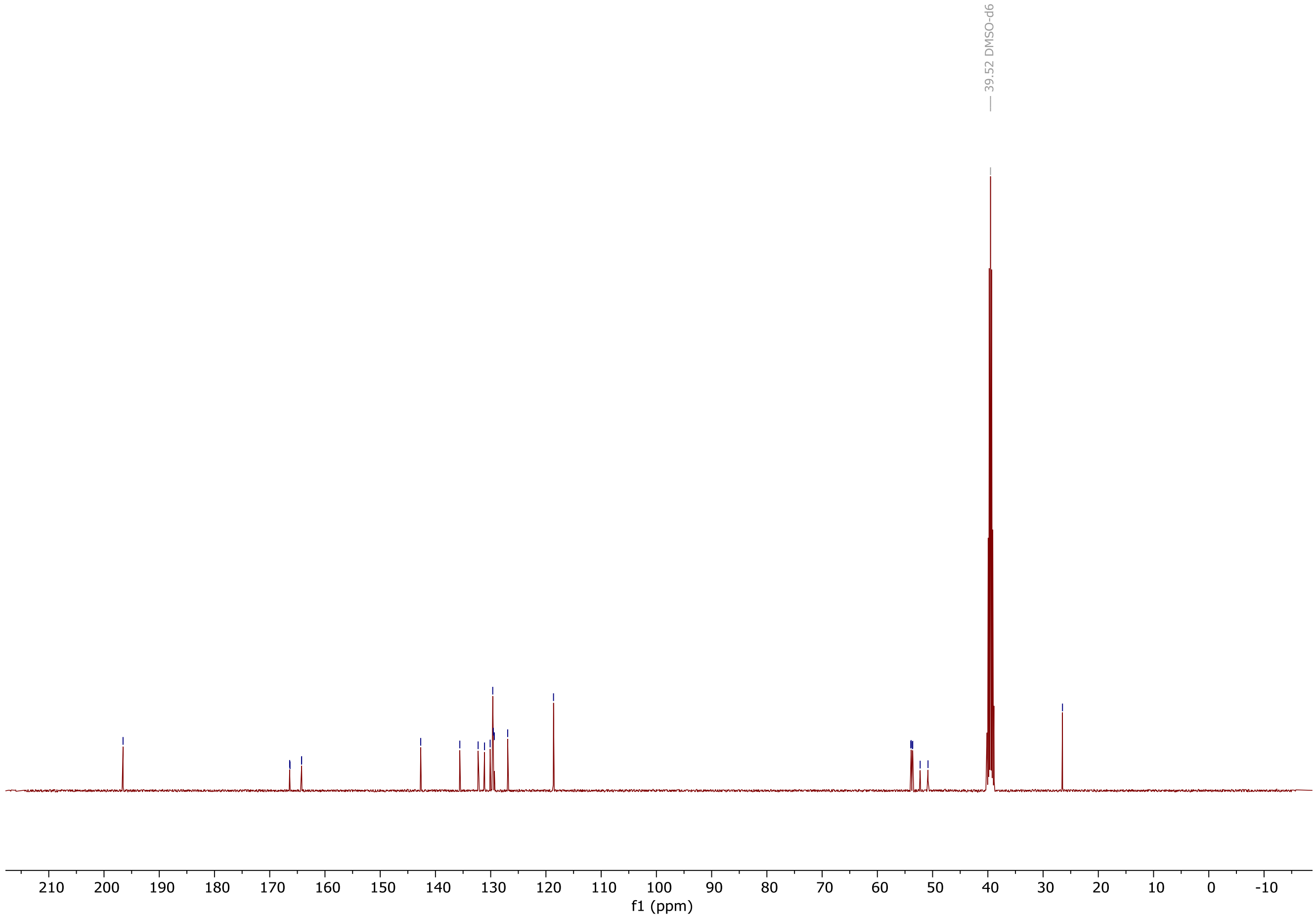
39.52 DMSO-d6
210 200 190 180 170 160 150 140 130 120 110 100 90 80 70 60 50 40 30 20 10 0 -10
f1 (ppm)

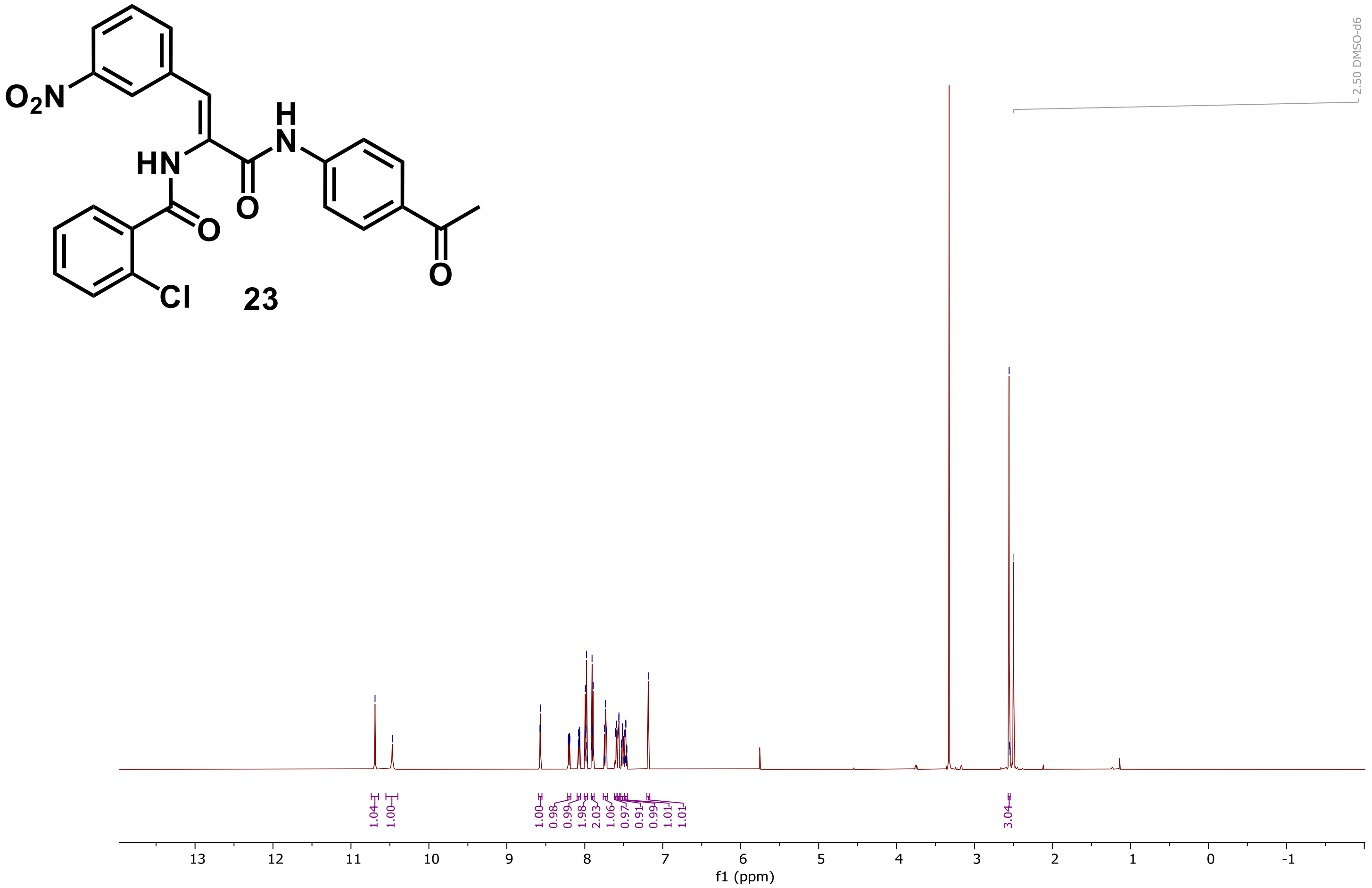
O2N
HN
H
N
O
O
Cl
O
23
2.50 DMSO-d6
f1 (ppm)

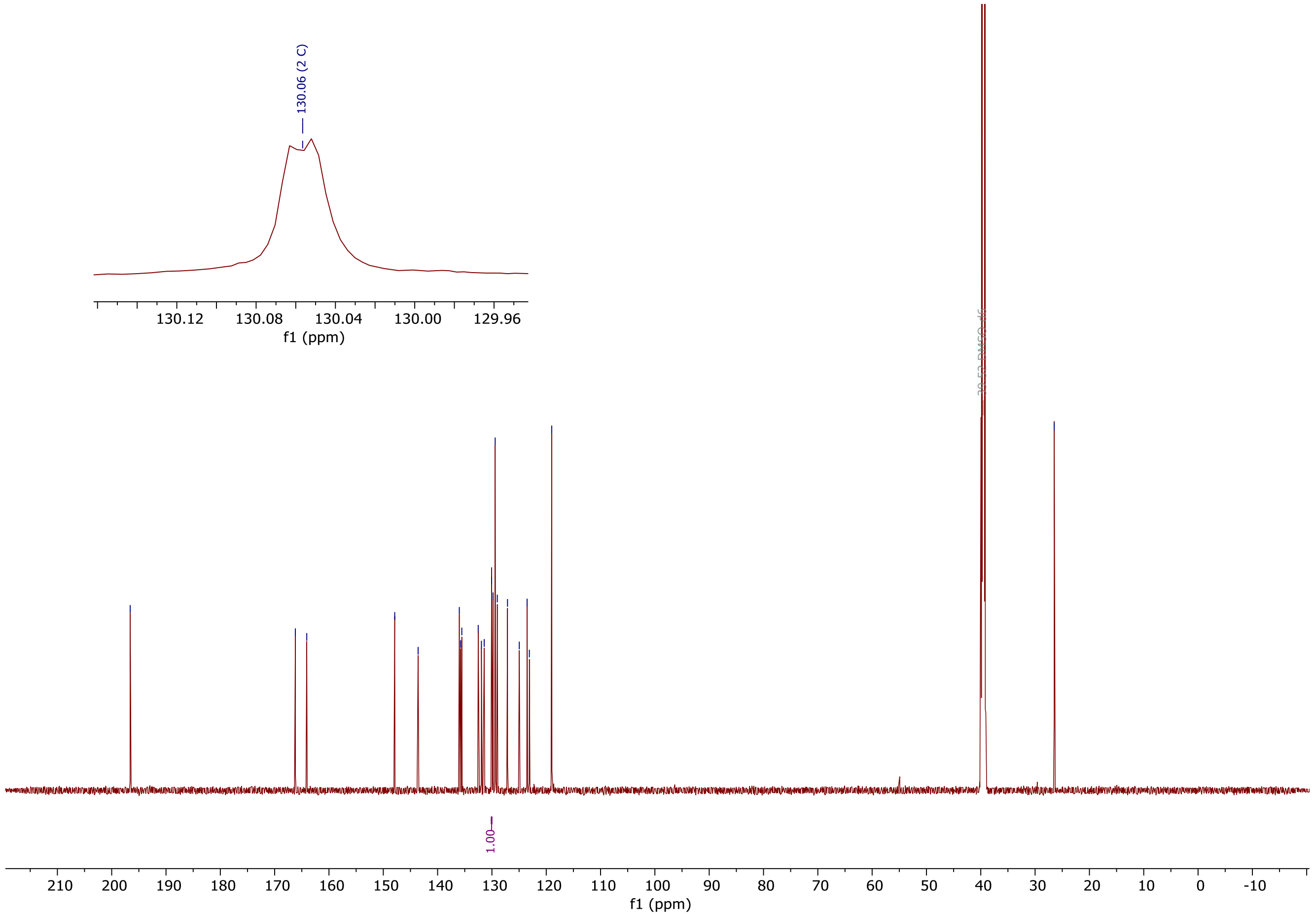
130.06 (2 C)
f1 (ppm)

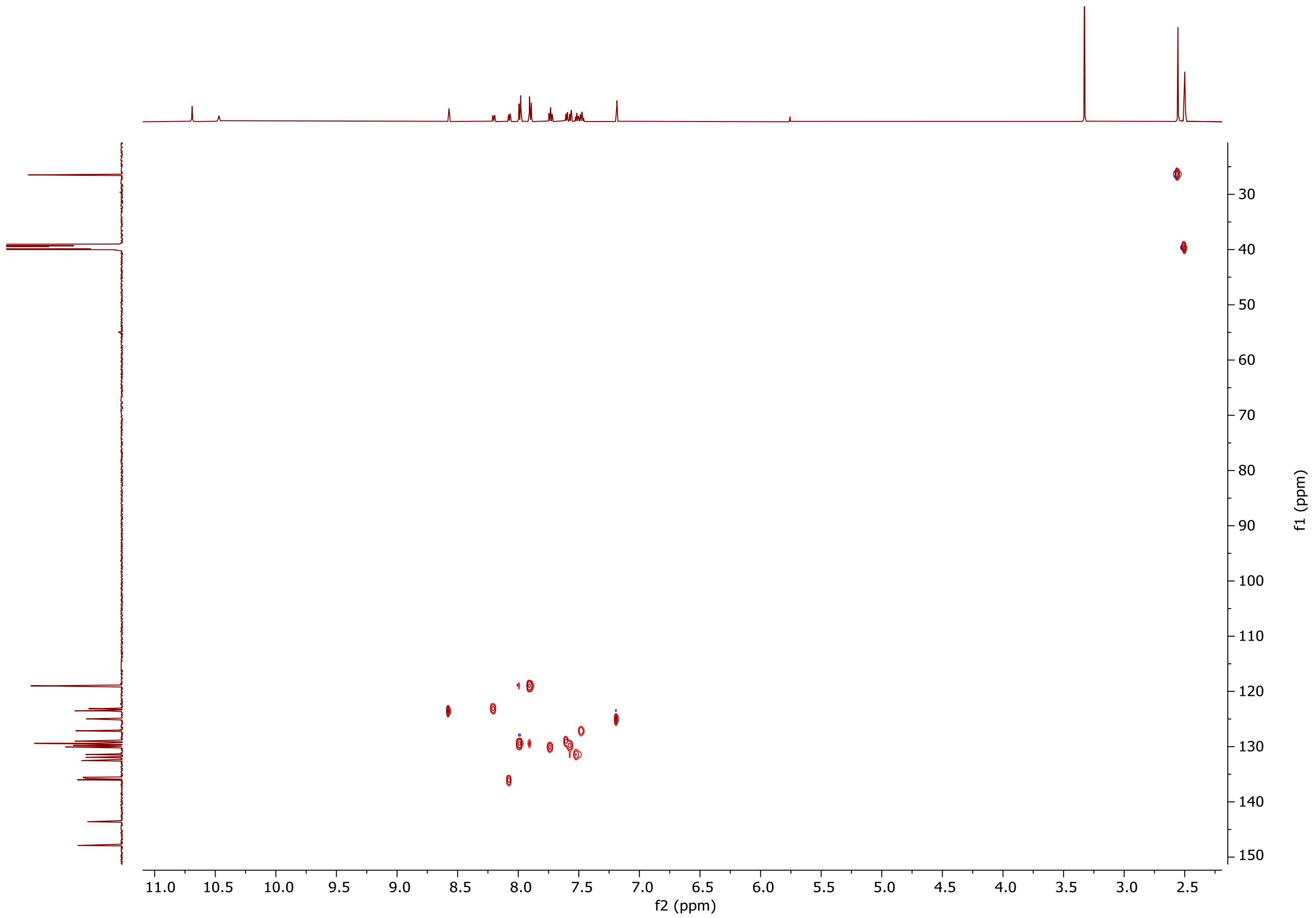
11.0 10.5 10.0 9.5 9.0 8.5 8.0 7.5 7.0 6.5 6.0 5.5 5.0 4.5 4.0 3.5 3.0 2.5
f2 (ppm)
30 40 50 60 70 80 90 100 110 120 130 140 150
f1 (ppm)

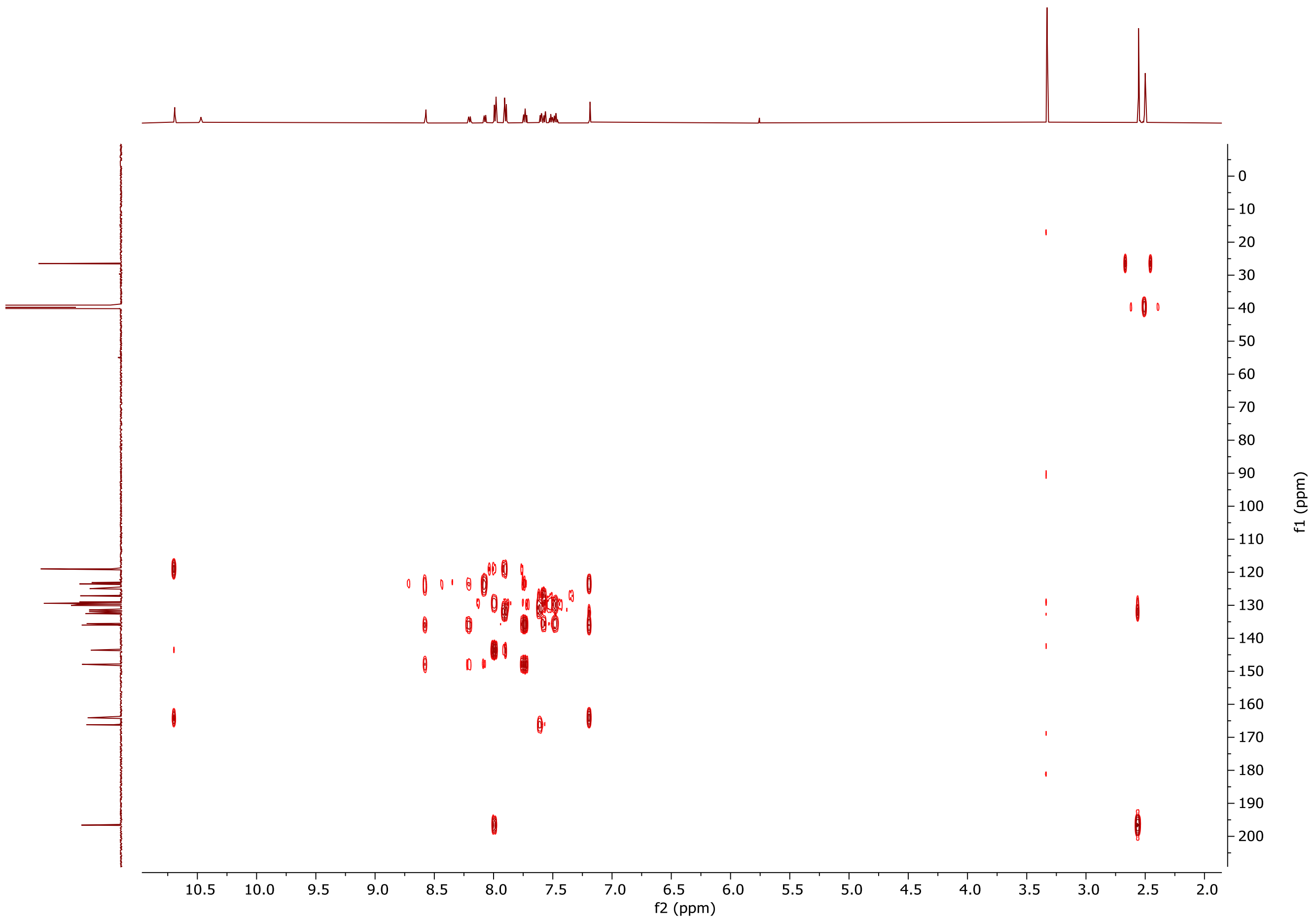
10.5 10.0 9.5 9.0 8.5 8.0 7.5 7.0 6.5 6.0 5.5 5.0 4.5 4.0 3.5 3.0 2.5 2.0
f2 (ppm)
0 10 20 30 40 50 60 70 80 90 100 110 120 130 140 150 160 170 180 190 200
f1 (ppm)

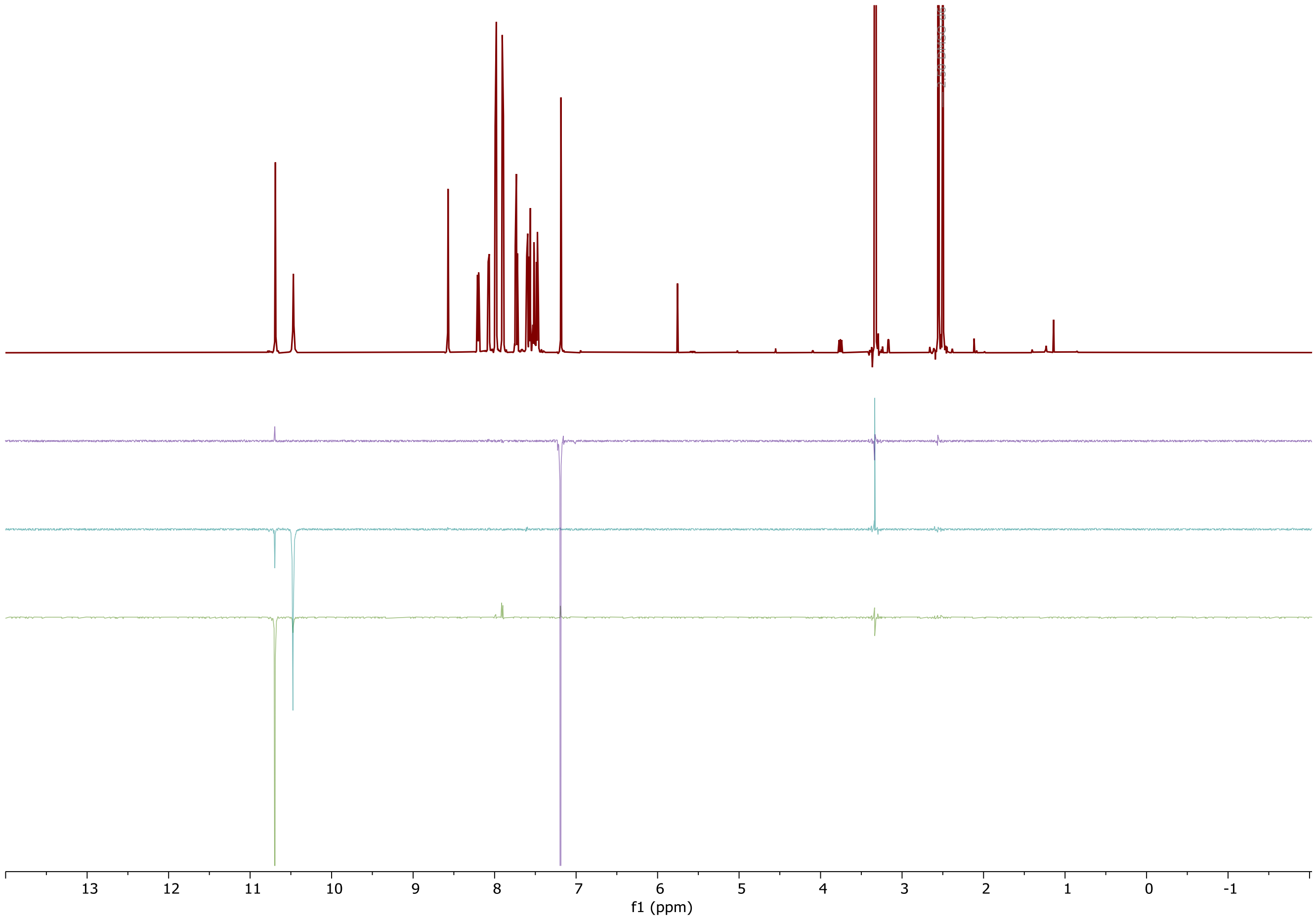

13
12
11
10
9
8
7
6
5
4
3
2
1
0
-1
f1 (ppm)

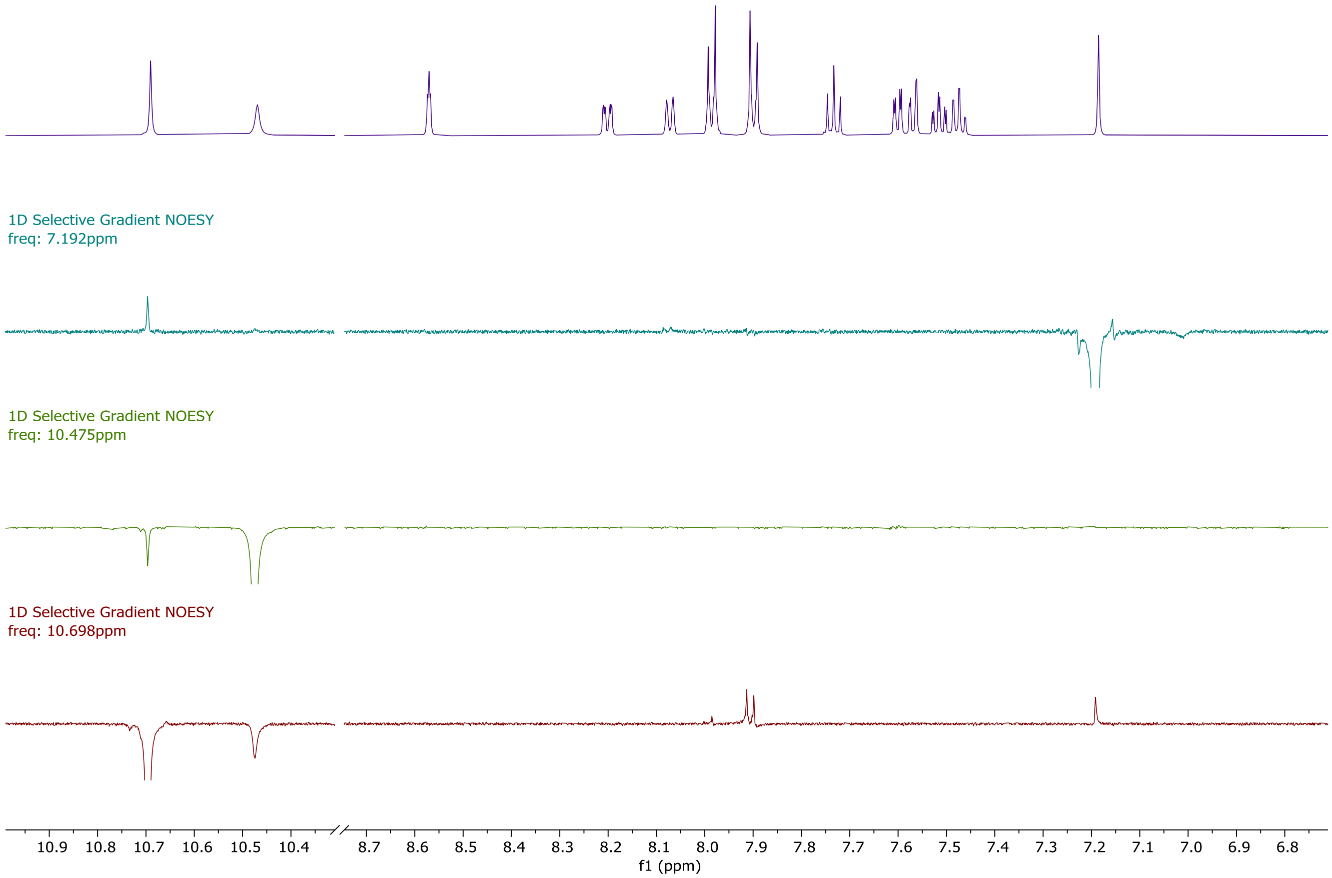

1D Selective Gradient NOESY
freq: 7.192ppm
1D Selective Gradient NOESY
freq: 10.475ppm
1D Selective Gradient NOESY
freq: 10.698ppm
10.9
10.8
10.7
10.6
10.5
10.4
8.7
8.6
8.5
8.4
8.3
8.2
8.1
8.0
7.9
7.8
7.7
7.6
7.5
7.4
7.3
7.2
7.1
7.0
6.9
6.8
f1 (ppm)

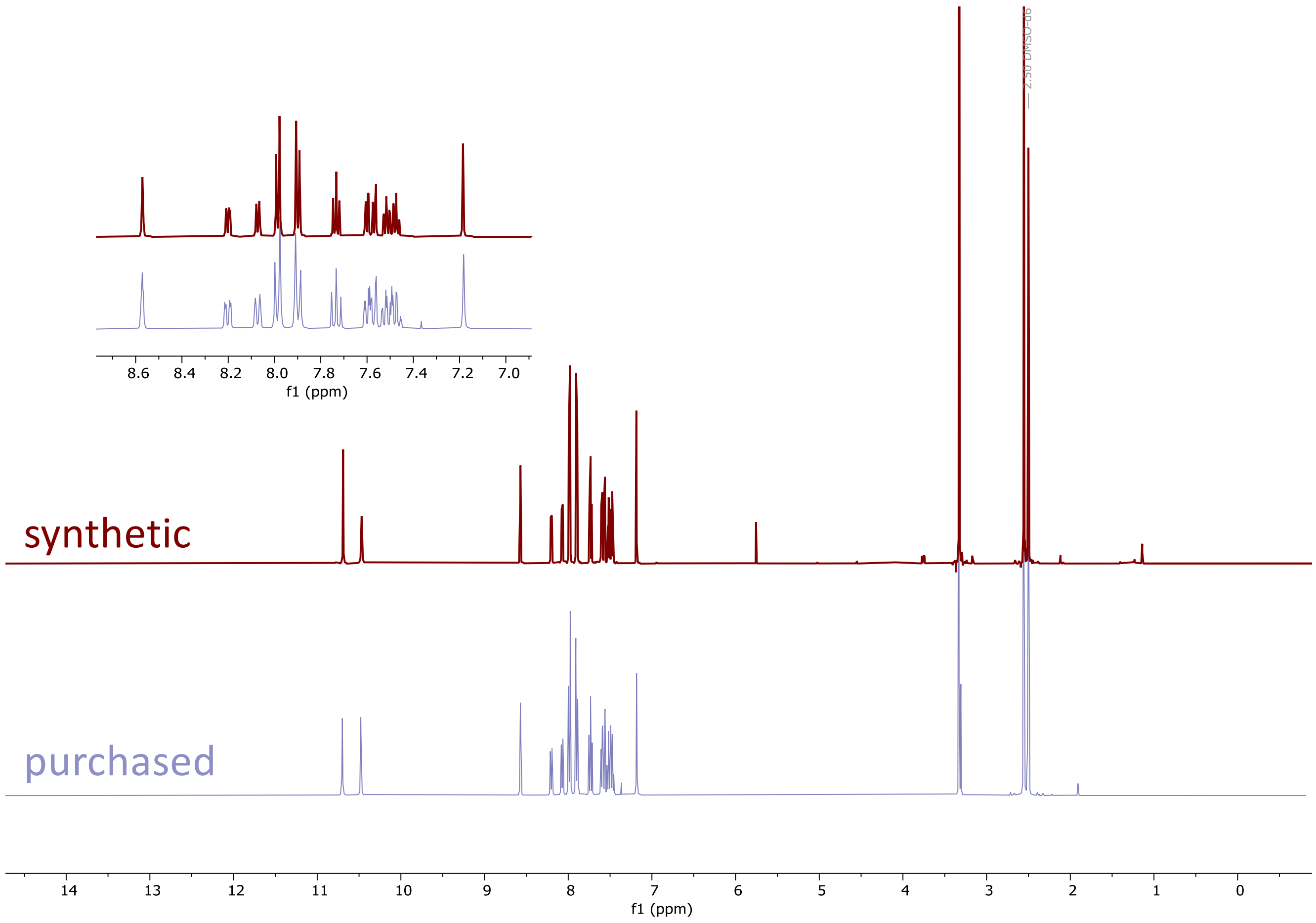
2.50 DMSO-d6
synthetic
purchased
8.6 8.4 8.2 8.0 7.8 7.6 7.4 7.2 7.0
f1 (ppm)
14 13 12 11 10 9 8 7 6 5 4 3 2 1 0
f1 (ppm)

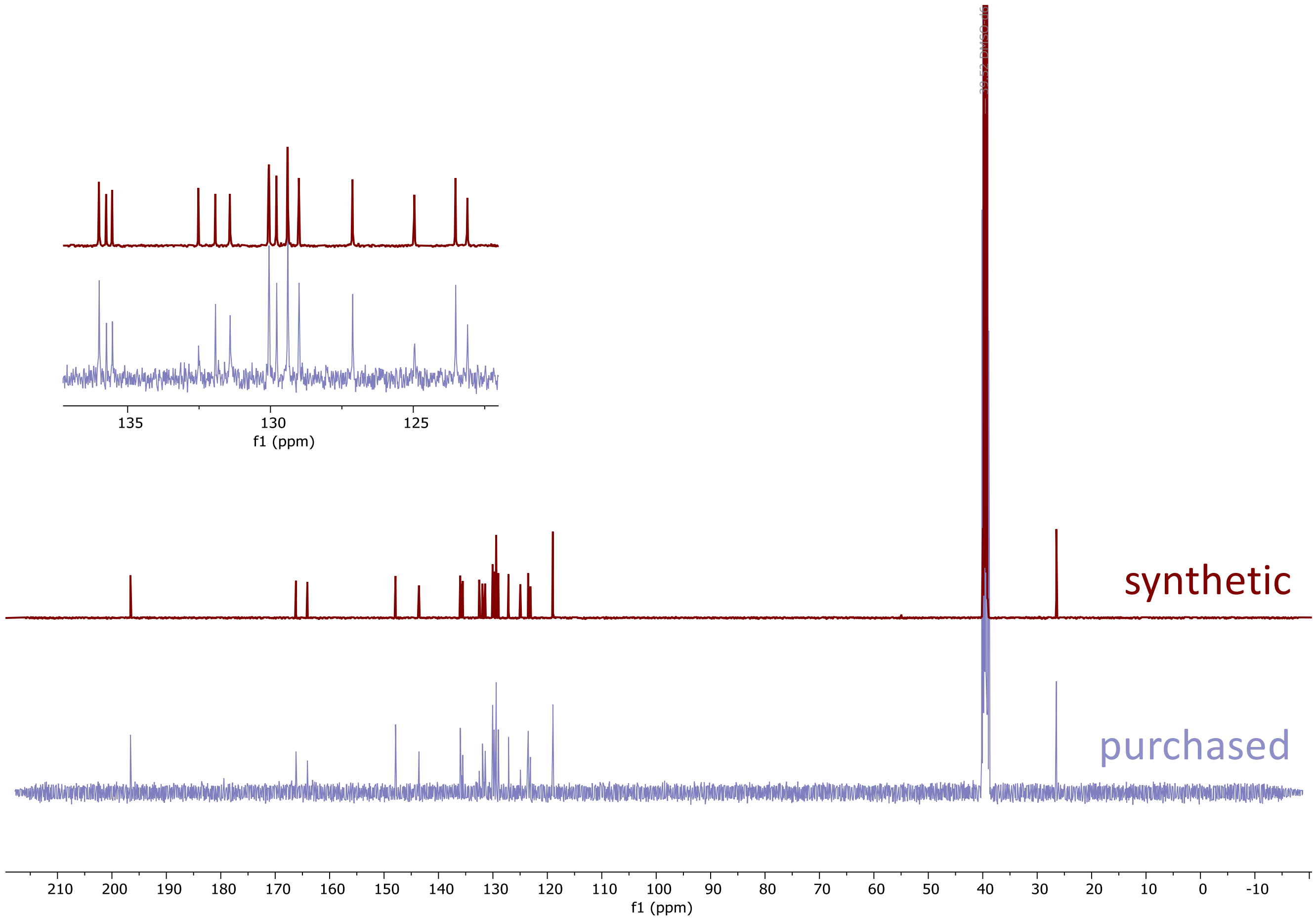
135 130 125
f1 (ppm)
synthetic
purchased
210 200 190 180 170 160 150 140 130 120 110 100 90 80 70 60 50 40 30 20 10 0 -10
f1 (ppm)

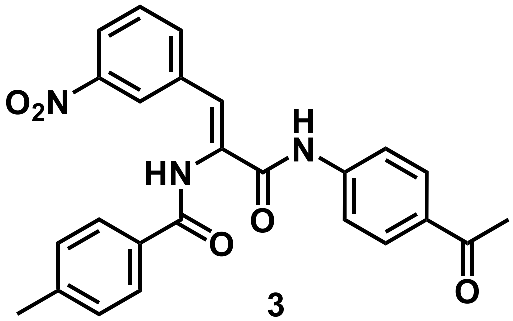
O2N
HN
H
N
O
O
O

3

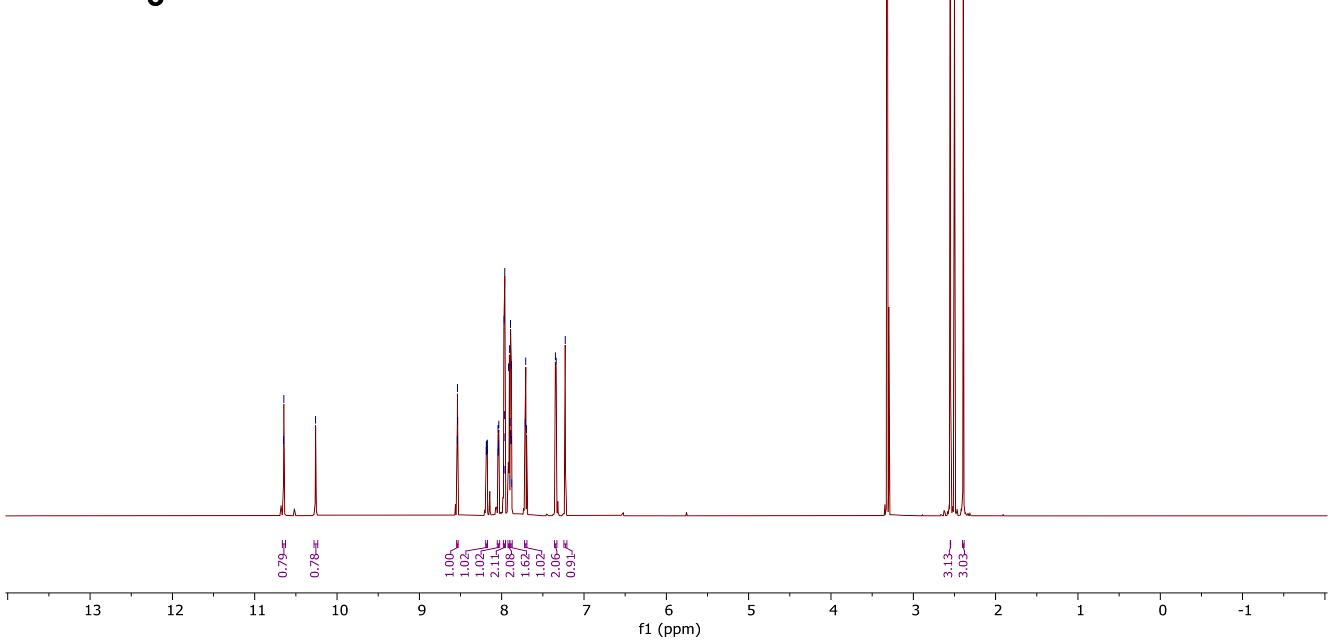
2.50 DMSO-d6
0.79
0.78
1.00
1.02
1.02
2.11
2.08
1.62
1.02
2.06
0.91
3.13
3.03
13 12 11 10 9 8 7 6 5 4 3 2 1 0 -1
f1 (ppm)

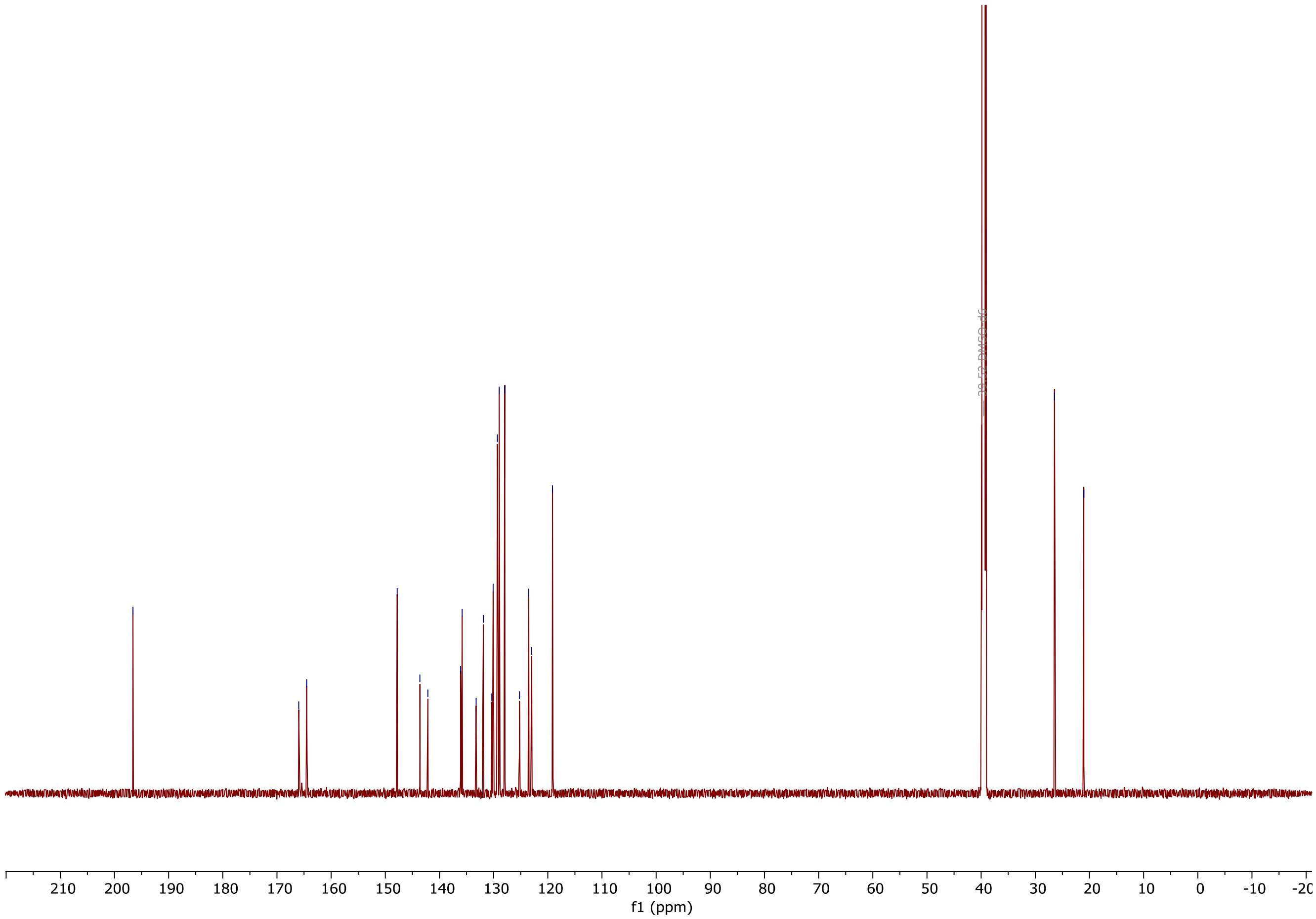
210 200 190 180 170 160 150 140 130 120 110 100 90 80 70 60 50 40 30 20 10 0 -10
f1 (ppm)

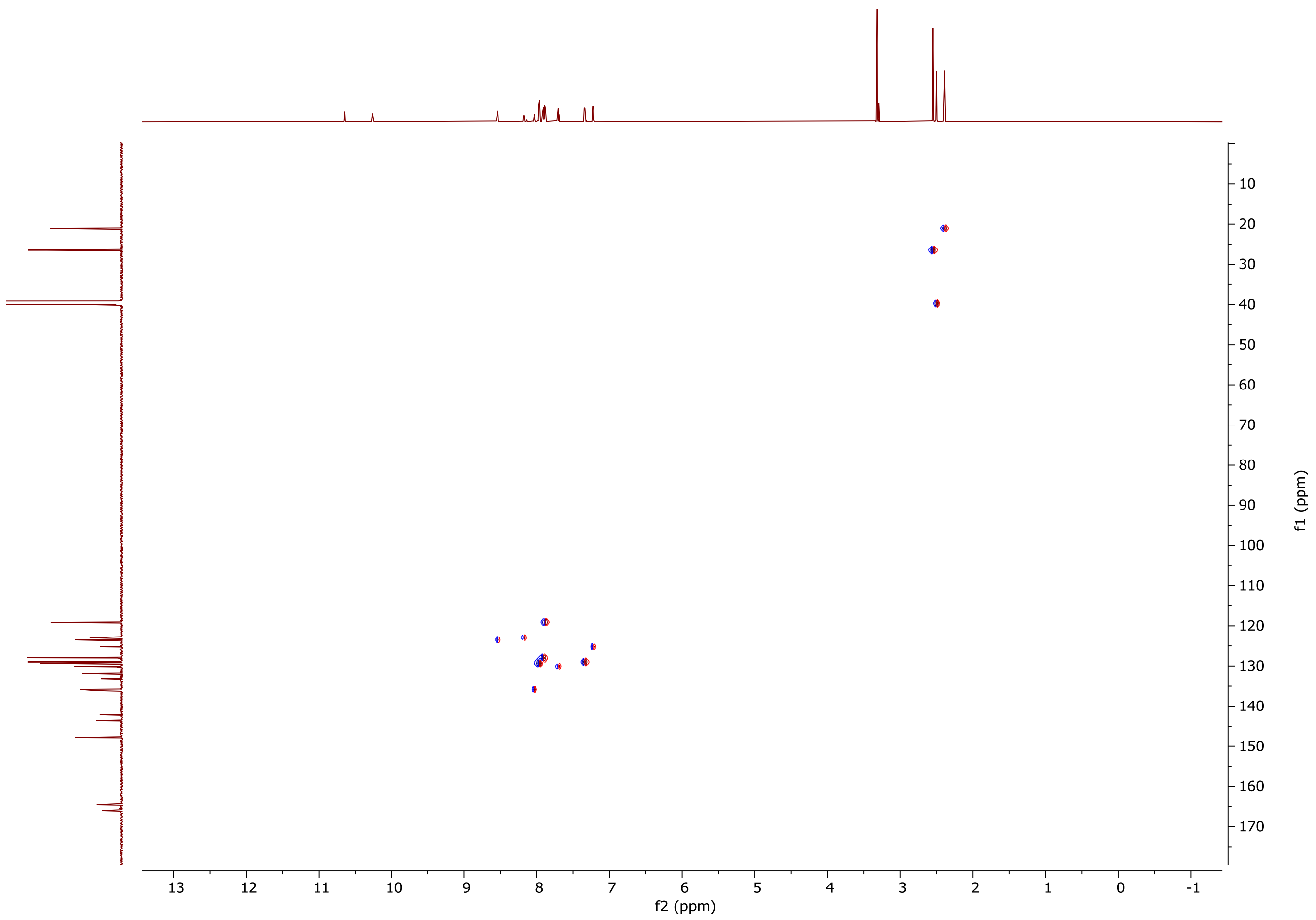
f1 (ppm)
f2 (ppm)

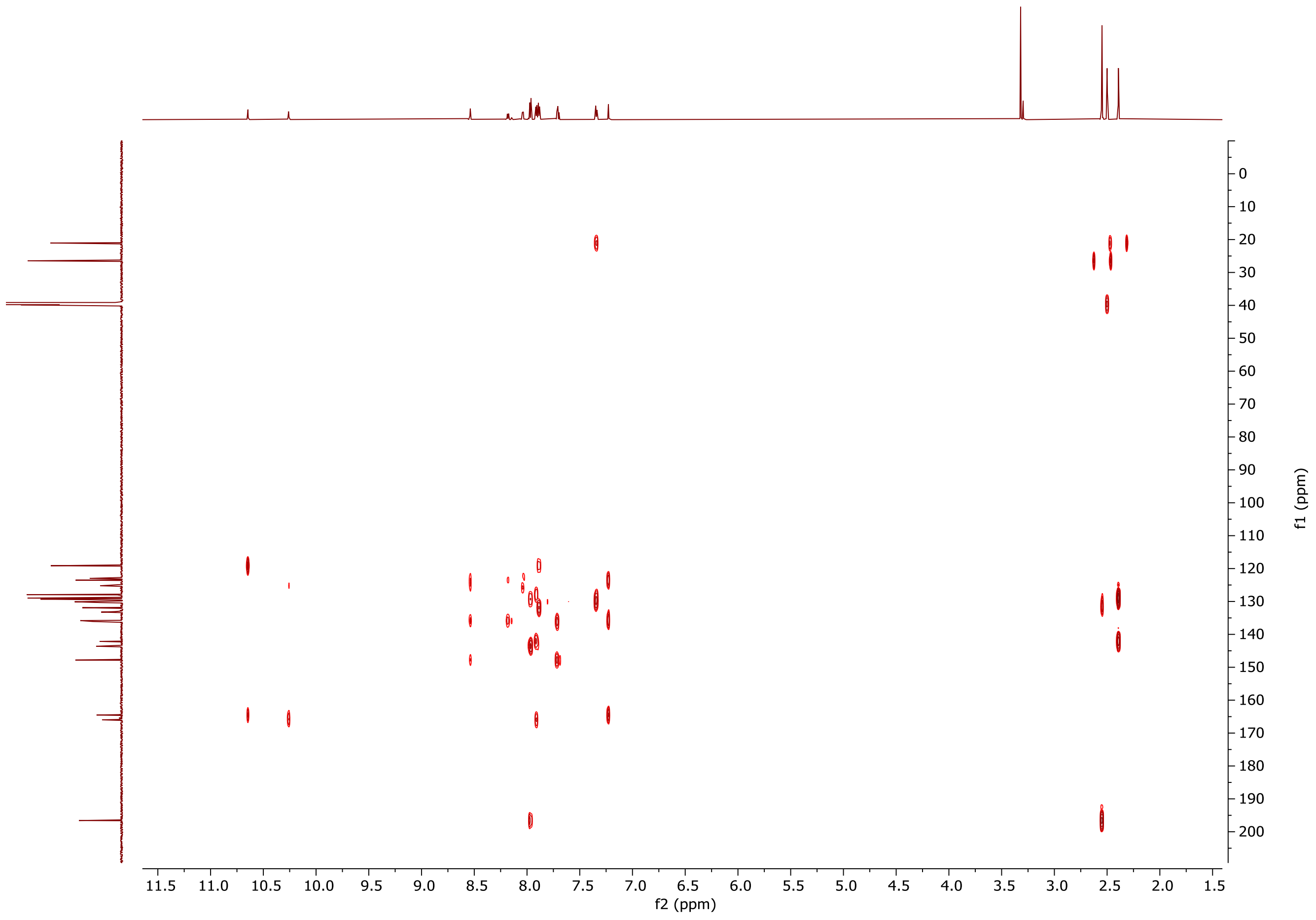
f1 (ppm)
f2 (ppm)

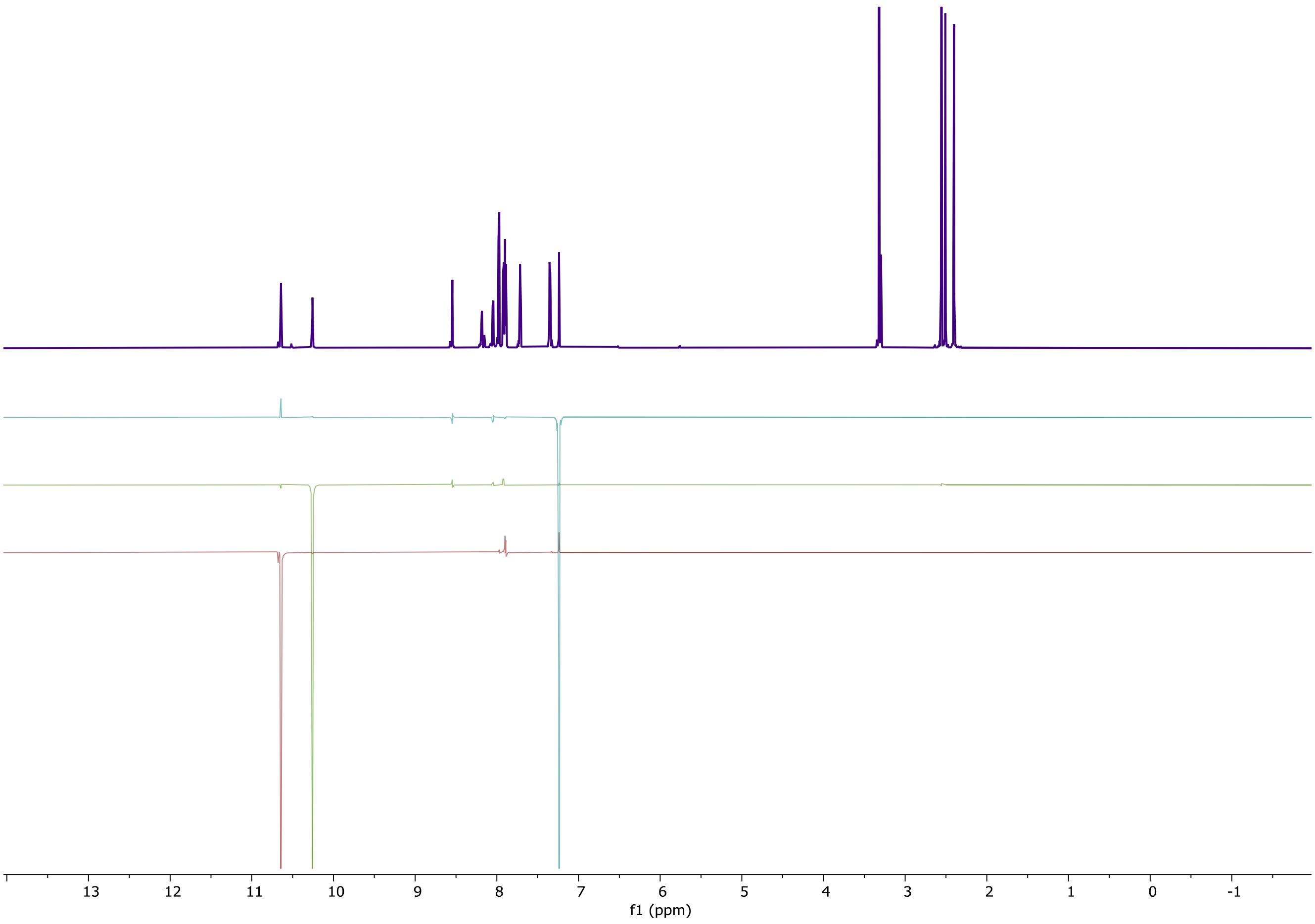

13
12
11
10
9
8
7
6
5
4
3
2
1
0
-1
f1 (ppm)

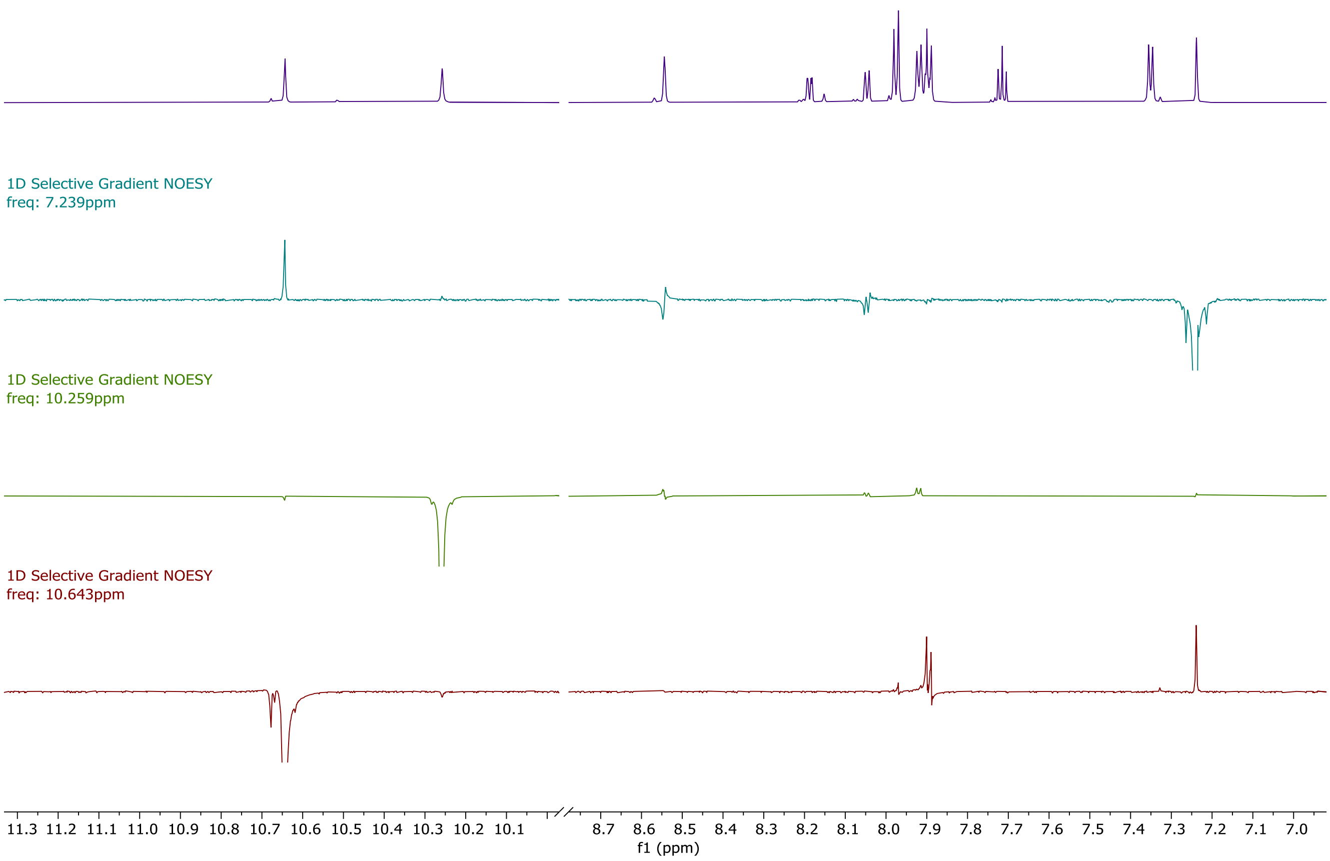

1D Selective Gradient NOESY
freq: 7.239ppm
1D Selective Gradient NOESY
freq: 10.259ppm
1D Selective Gradient NOESY
freq: 10.643ppm
11.3 11.2 11.1 11.0 10.9 10.8 10.7 10.6 10.5 10.4 10.3 10.2 10.1
8.7 8.6 8.5 8.4 8.3 8.2 8.1 8.0 7.9 7.8 7.7 7.6 7.5 7.4 7.3 7.2 7.1 7.0
f1 (ppm)

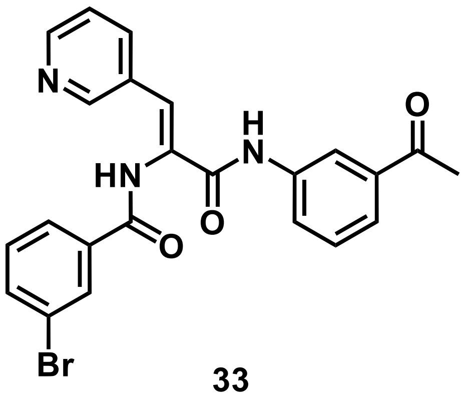
N
H
N
O
HN
O
O
Br

33

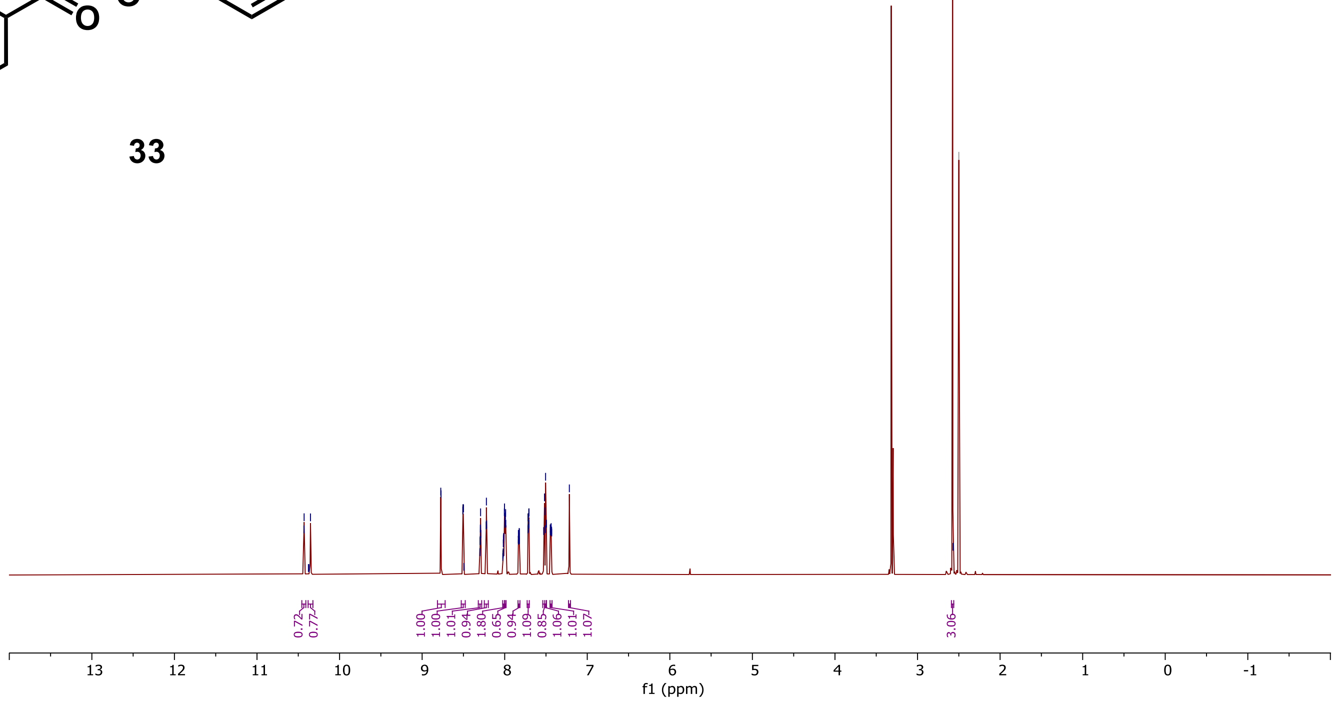
2.50 DMSO-d6
0.72
0.77
1.00
1.00
1.01
0.94
1.80
0.65
0.94
1.09
0.85
1.06
1.01
1.07
3.06
13 12 11 10 9 8 7 6 5 4 3 2 1 0 -1
f1 (ppm)

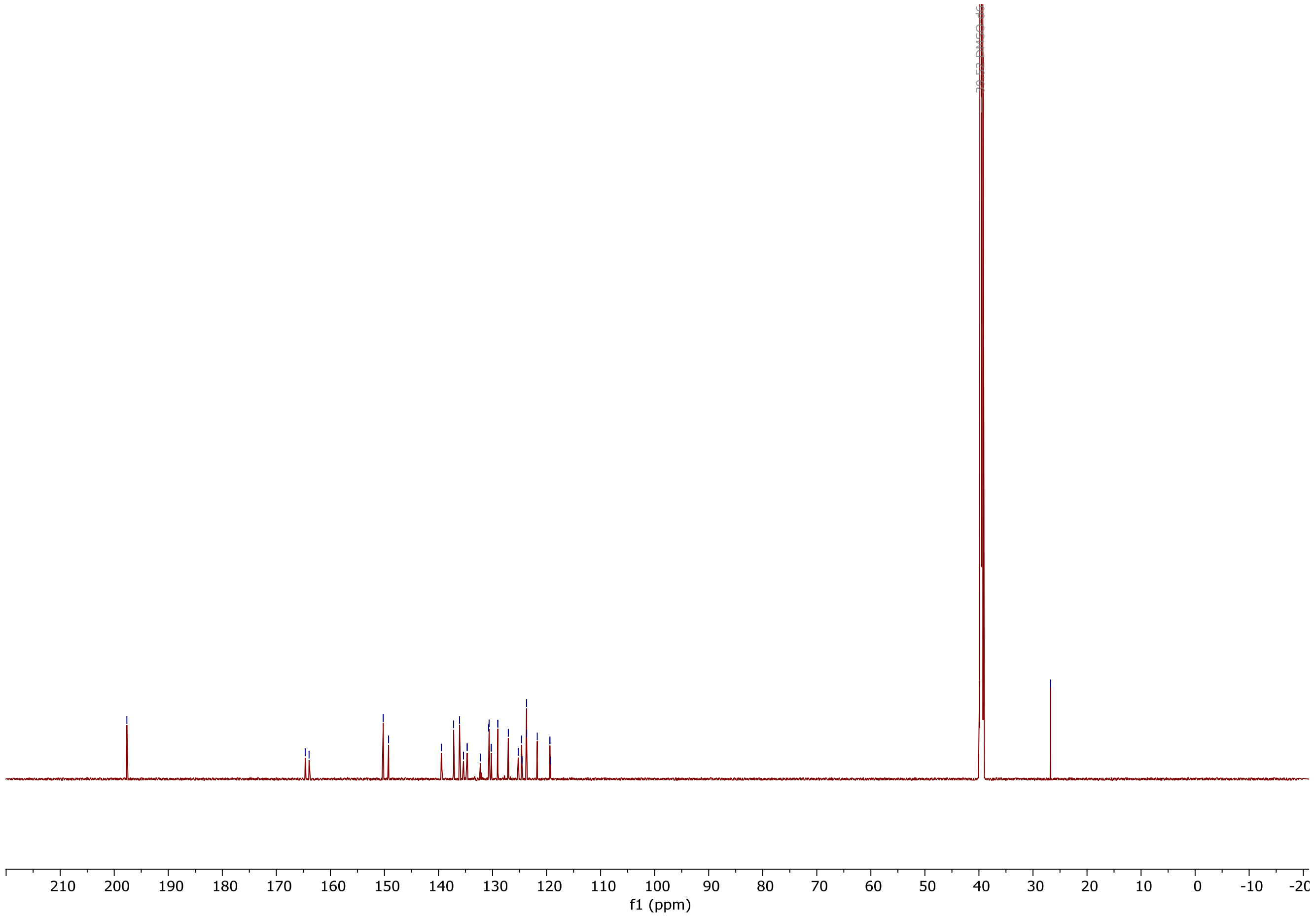
210 200 190 180 170 160 150 140 130 120 110 100 90 80 70 60 50 40 30 20 10 0 -10 -20
f1 (ppm)

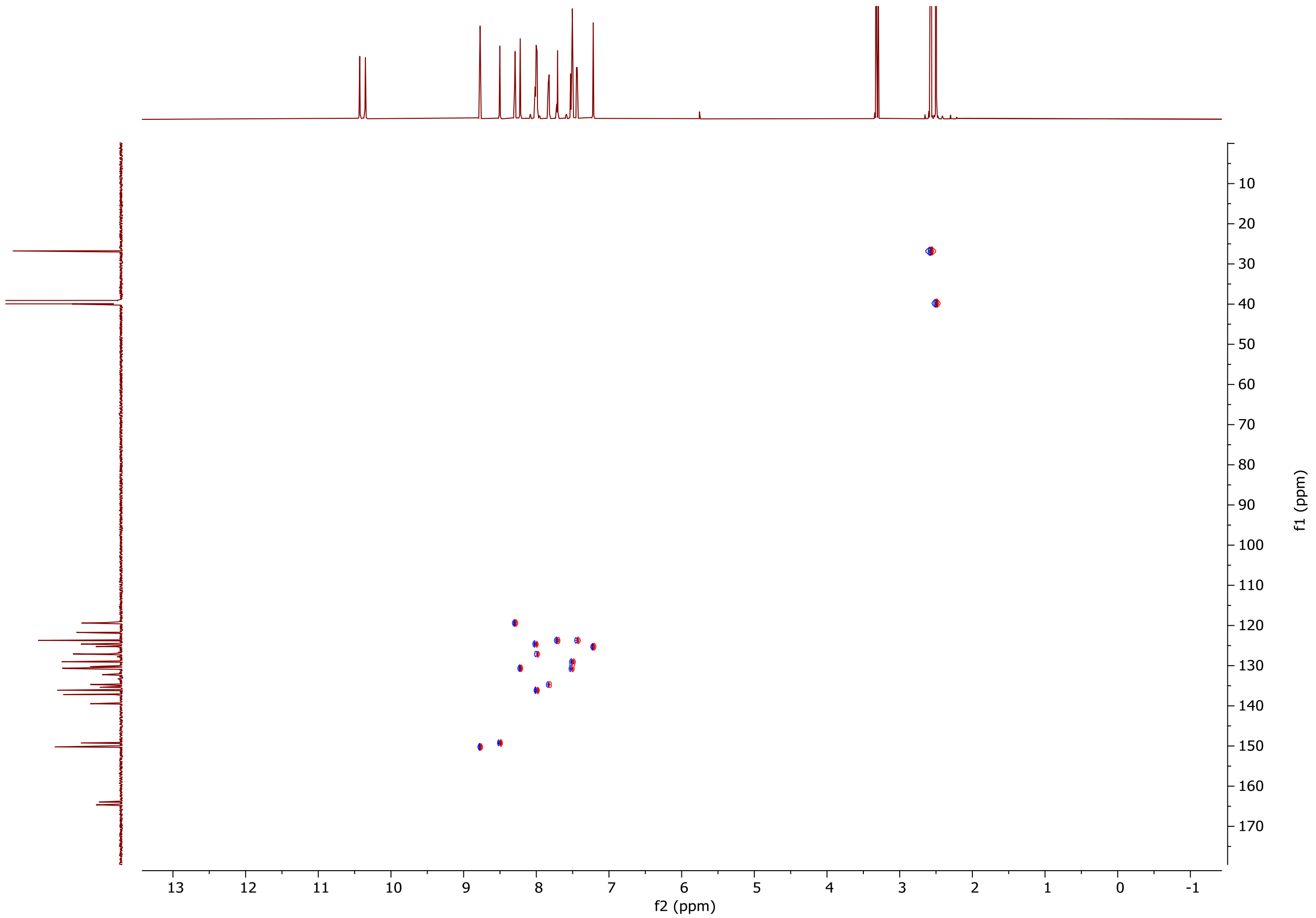

f1 (ppm)
f2 (ppm)

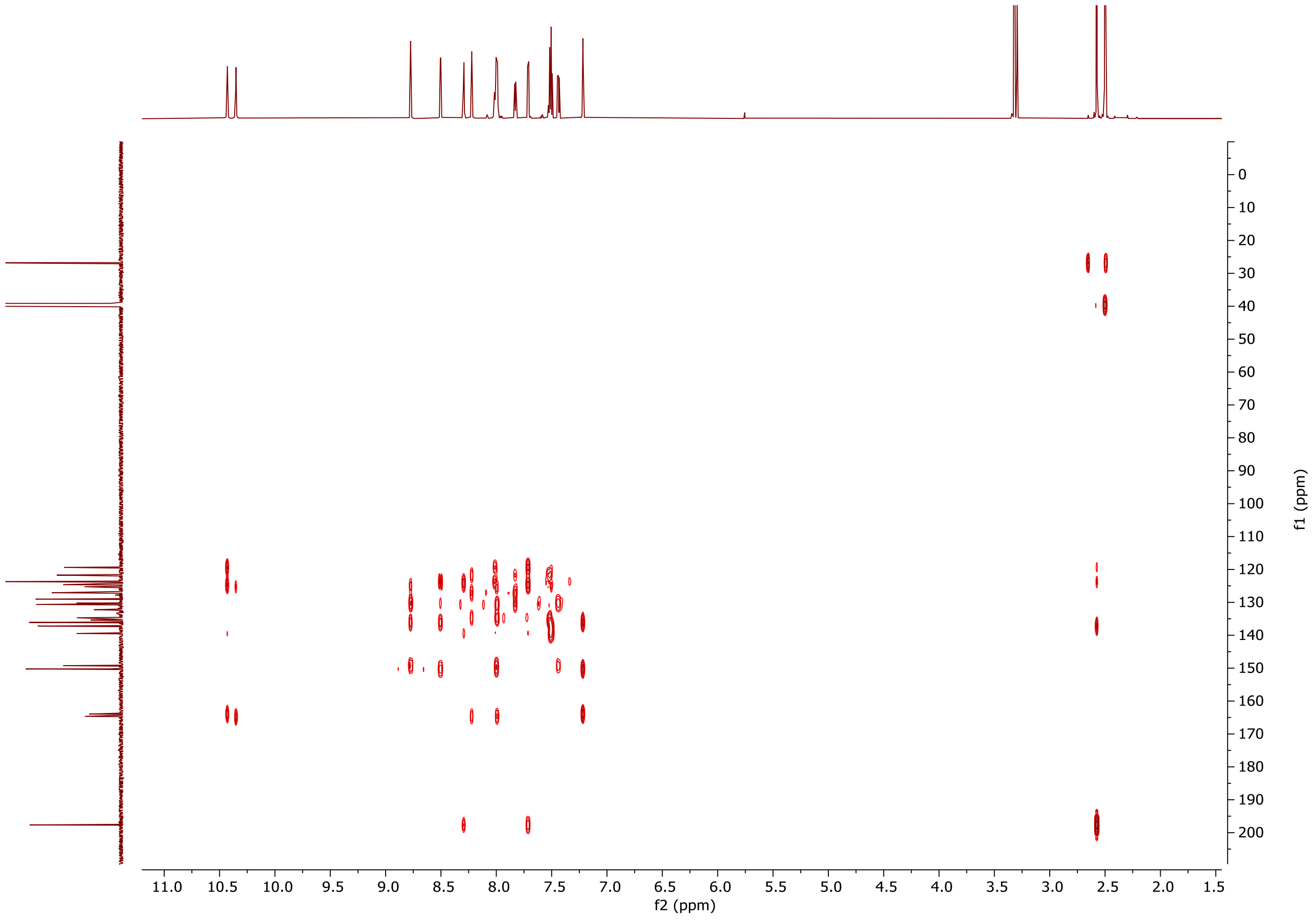

f1 (ppm)
f2 (ppm)

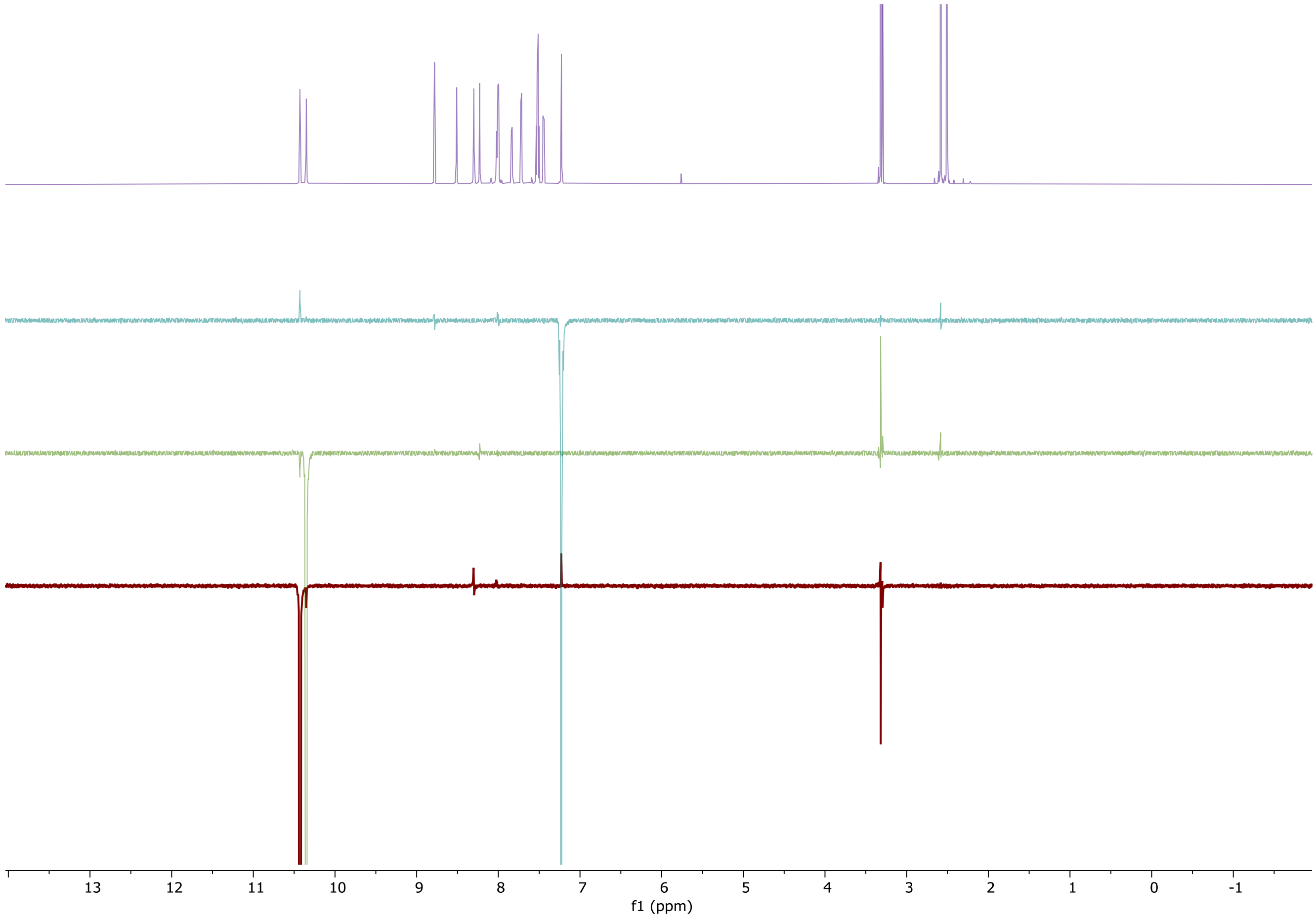
13
12
11
10
9
8
7
6
5
4
3
2
1
0
-1
f1 (ppm)

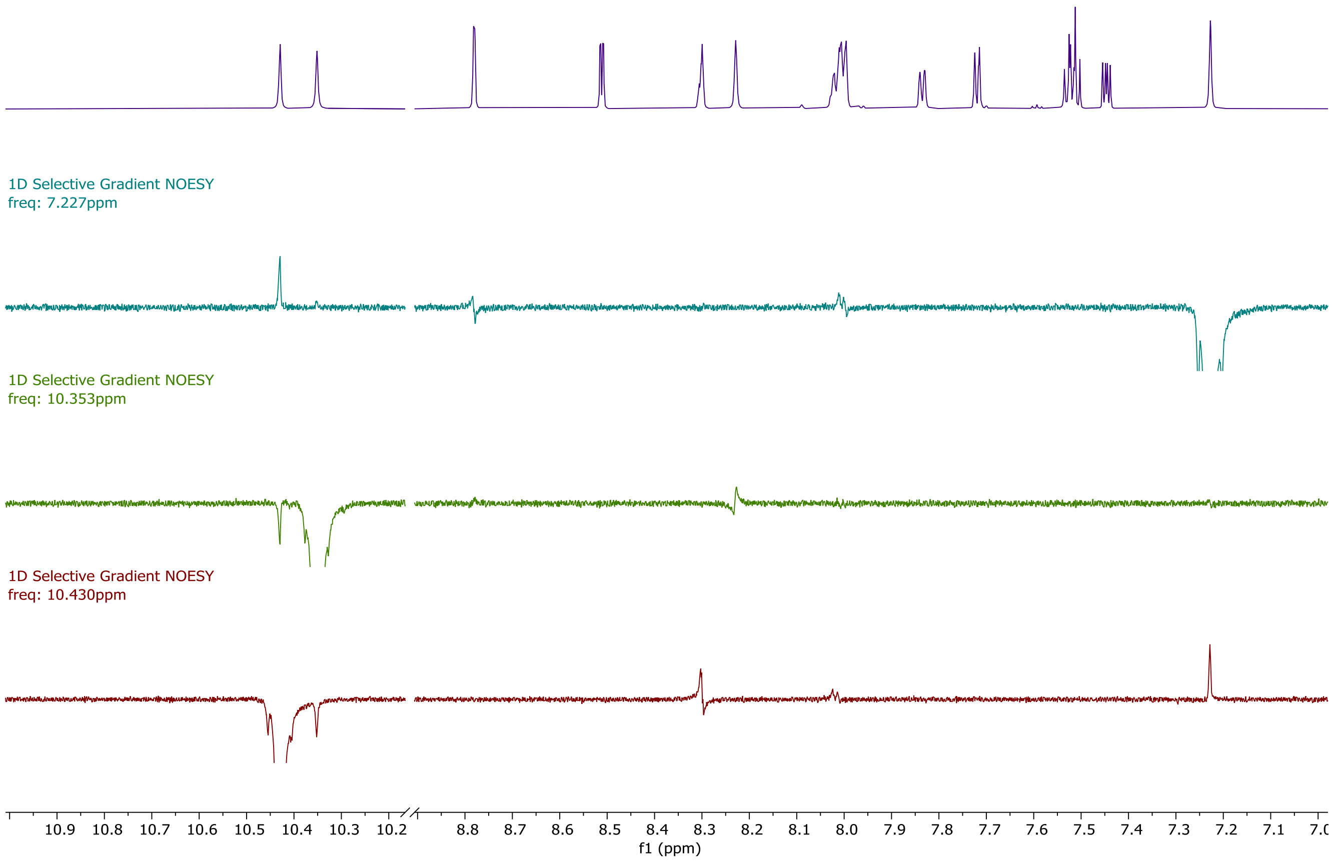
1D Selective Gradient NOESY
freq: 7.227ppm
1D Selective Gradient NOESY
freq: 10.353ppm
1D Selective Gradient NOESY
freq: 10.430ppm
10.9
10.8
10.7
10.6
10.5
10.4
10.3
10.2
8.8
8.7
8.6
8.5
8.4
8.3
8.2
8.1
8.0
7.9
7.8
7.7
7.6
7.5
7.4
7.3
7.2
7.1
f1 (ppm)

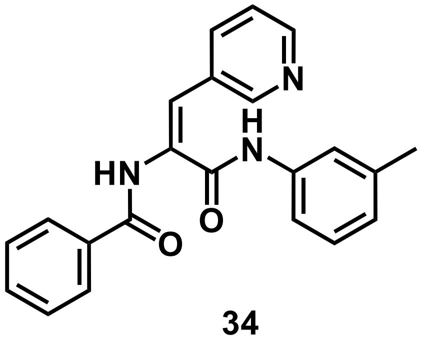

34

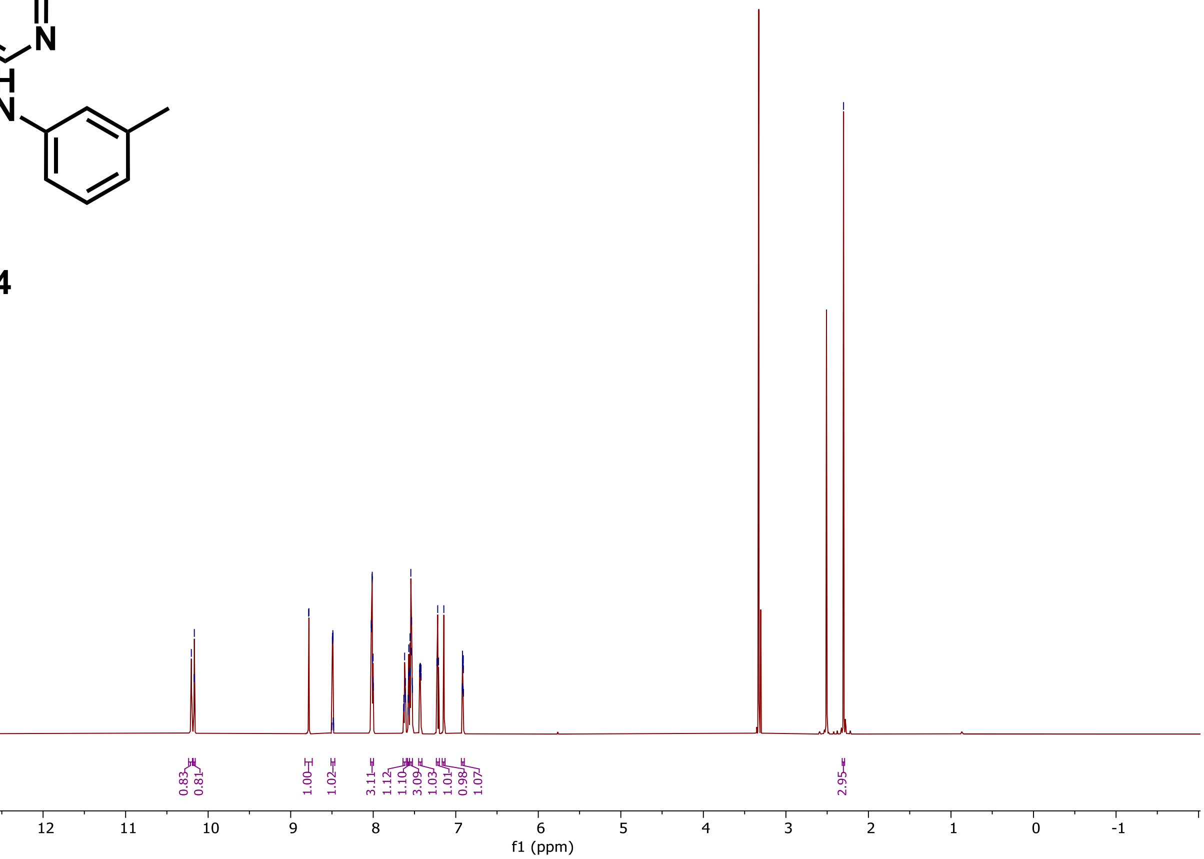
13 12 11 10 9 8 7 6 5 4 3 2 1 0 -1
f1 (ppm)

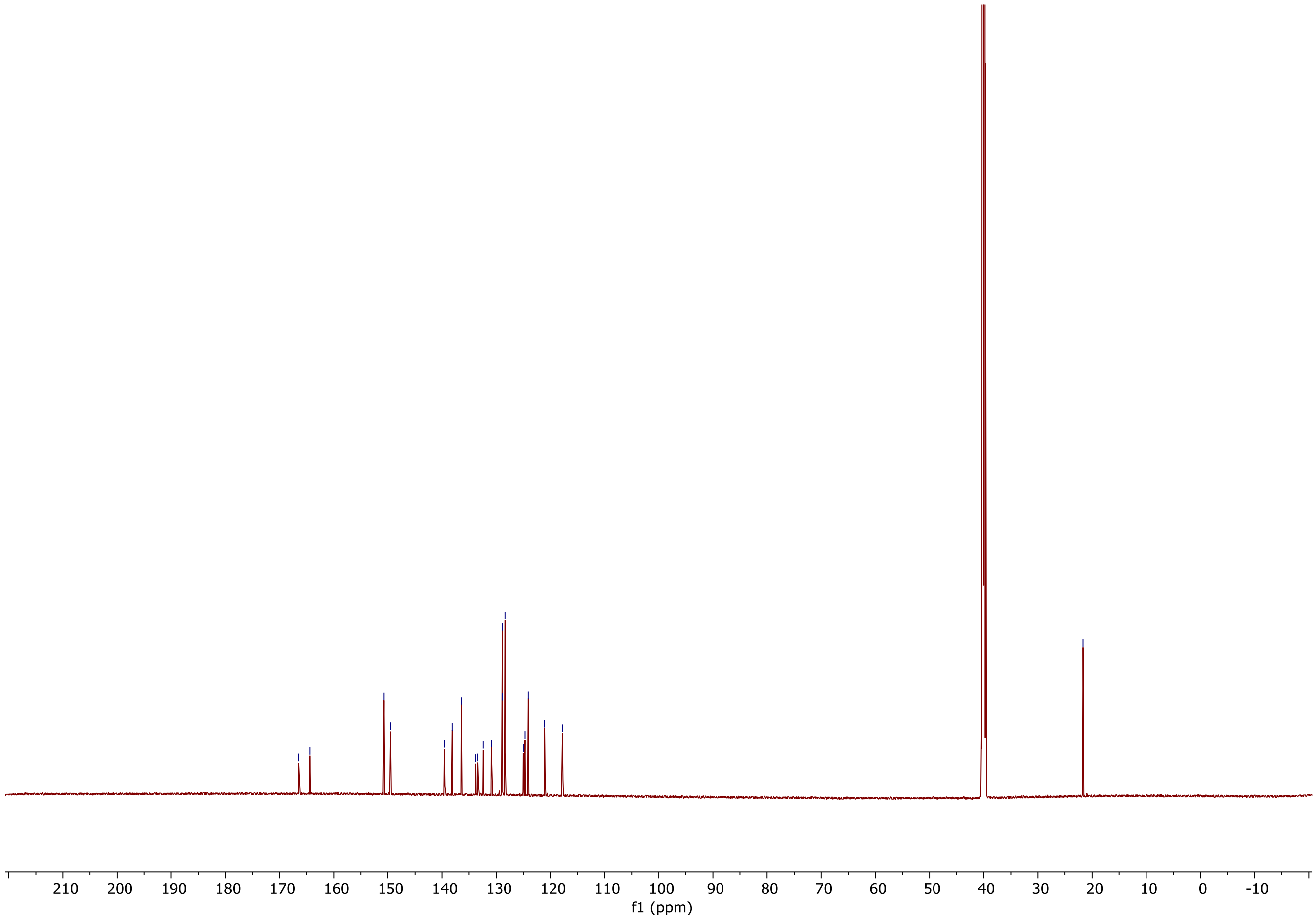
210 200 190 180 170 160 150 140 130 120 110 100 90 80 70 60 50 40 30 20 10 0 -10
f1 (ppm)

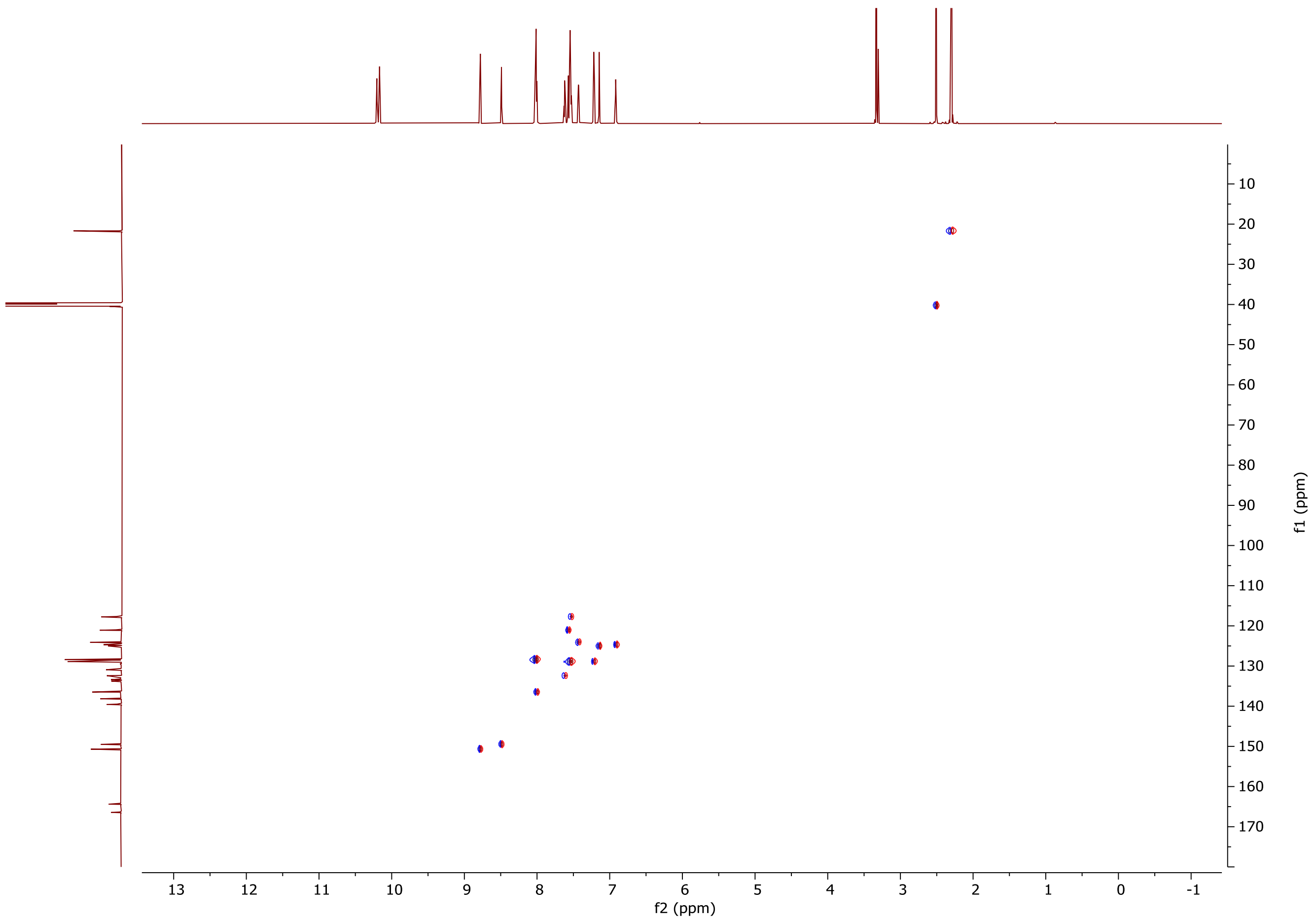
f2 (ppm)
f1 (ppm)

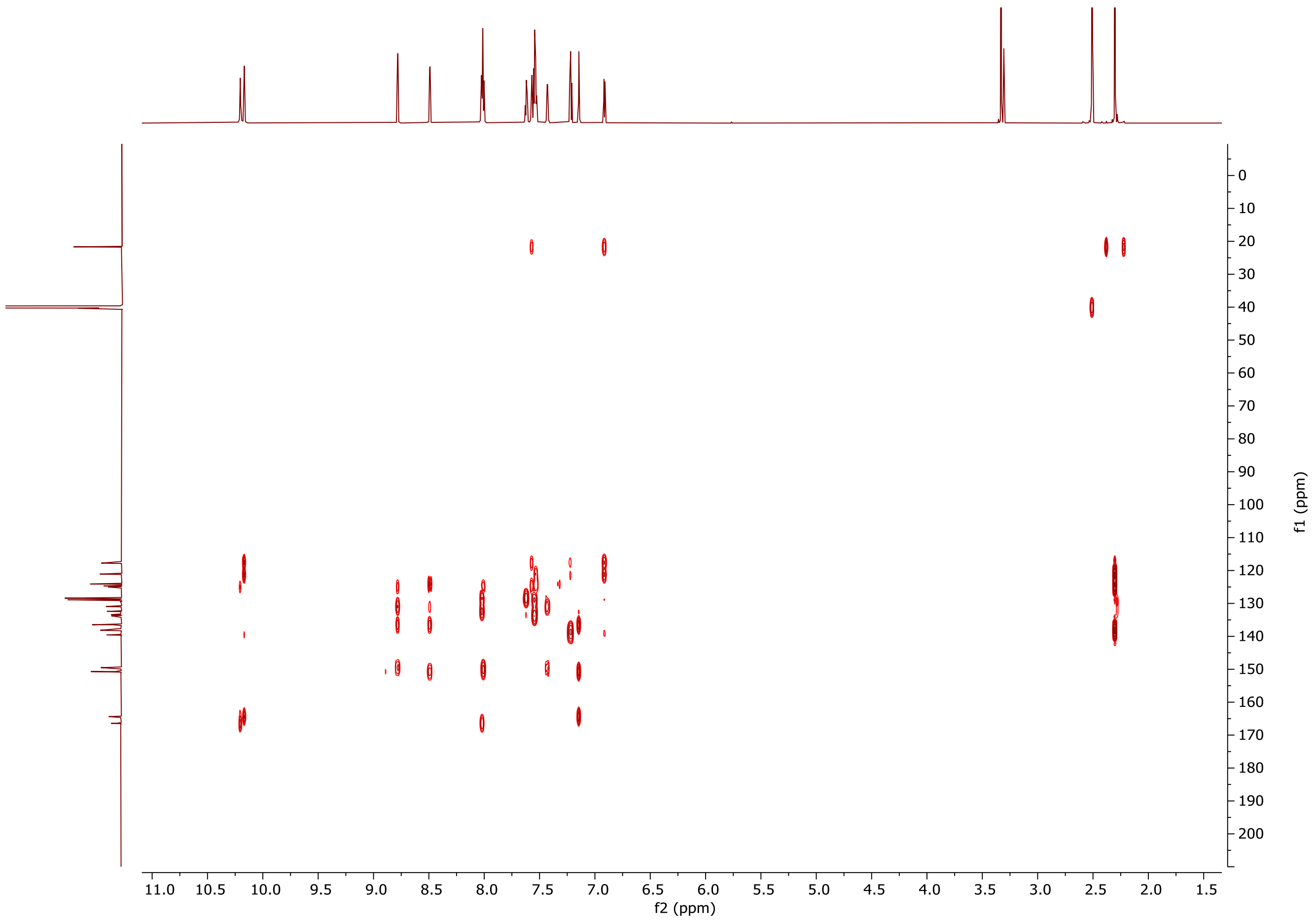
f2 (ppm)
f1 (ppm)

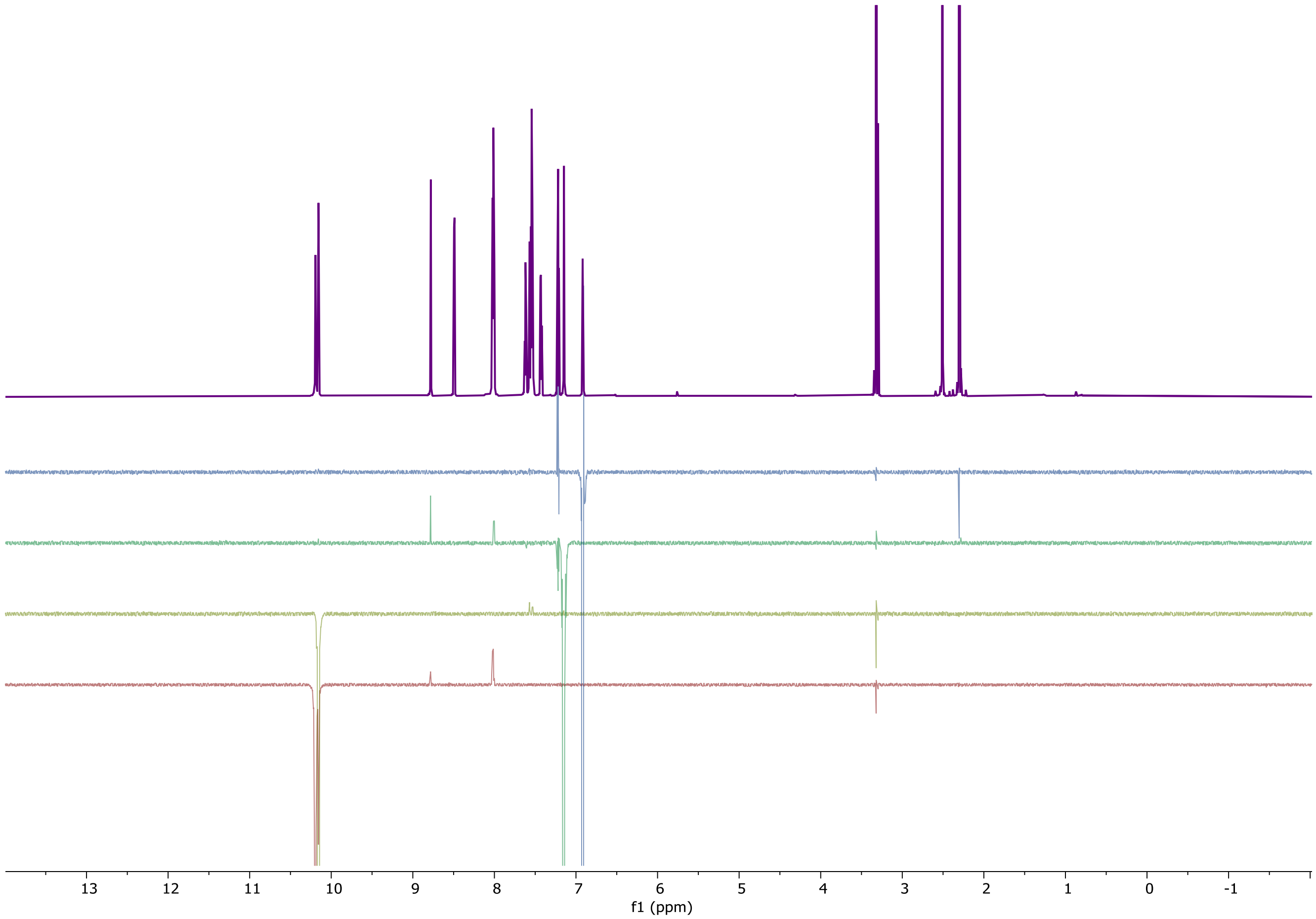
13 12 11 10 9 8 7 6 5 4 3 2 1 0 -1
f1 (ppm)

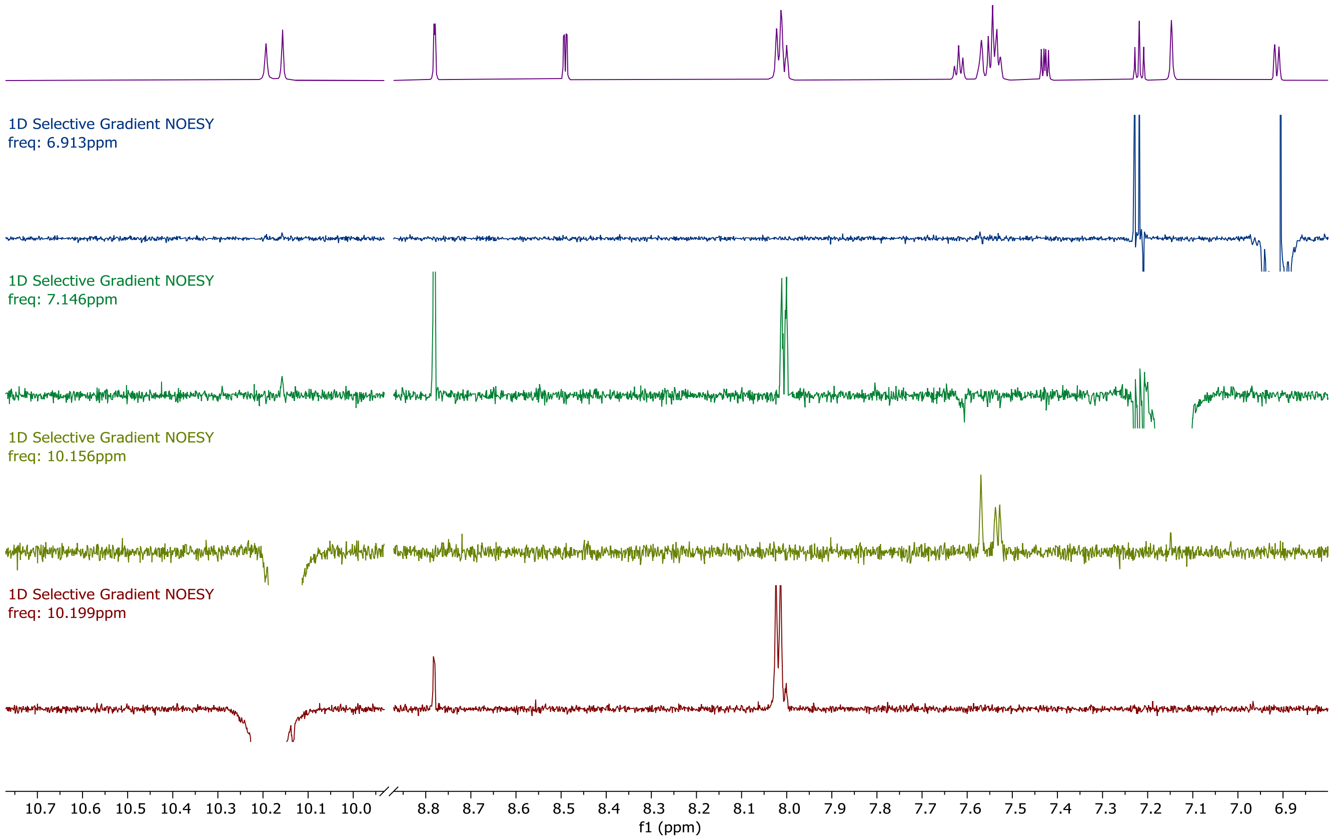
1D Selective Gradient NOESY
freq: 6.913ppm
1D Selective Gradient NOESY
freq: 7.146ppm
1D Selective Gradient NOESY
freq: 10.156ppm
1D Selective Gradient NOESY
freq: 10.199ppm
10.7 10.6 10.5 10.4 10.3 10.2 10.1 10.0 8.8 8.7 8.6 8.5 8.4 8.3 8.2 8.1 8.0 7.9 7.8 7.7 7.6 7.5 7.4 7.3 7.2 7.1 7.0 6.9
f1 (ppm)
